# Diagnostic accuracy of nucleic acid amplification tests for human intestinal nematode infections: A systematic review and meta-analysis

**DOI:** 10.1371/journal.pntd.0013974

**Published:** 2026-02-11

**Authors:** Nalini Jayakody, Catherine A. Gordon, Nuwan Wickramasinghe, Anjana Silva, Susiji Wickramasinghe, Kosala Weerakoon

**Affiliations:** 1 Department of Parasitology, Faculty of Medicine, Wayamba University of Sri Lanka, Kuliyapitiya, Sri Lanka; 2 Department of Parasitology, Faculty of Medicine and Allied Sciences, Rajarata University of Sri Lanka, Saliyapura, Sri Lanka; 3 QIMR Berghofer Medical Research Institute, Infection and Inflammation Program, Applied Tropical and Molecular Parasitology Laboratory, Brisbane, Queensland, Australia; 4 Faculty of Medicine, The University of Queensland, Brisbane, Queensland, Australia; 5 Center for Tropical Health and Emerging Diseases, QIMR Berghofer Medical Research Institute, Brisbane, Queensland, Australia; 6 Department of Community Medicine, Faculty of Medicine and Allied Sciences, Rajarata University of Sri Lanka, Saliyapura, Sri Lanka; 7 Department of Parasitology, Faculty of Medicine, University of Peradeniya, Kandy, Sri Lanka; Babol University of Medical Science, IRAN, ISLAMIC REPUBLIC OF

## Abstract

**Introduction:**

The use of diverse diagnostic methods in the absence of a definitive gold standard makes it challenging to determine the most appropriate test for diagnosing human intestinal nematode infections (HINIs), particularly across various clinical settings with varying endemicity. The ideal diagnostic method should be feasible, cost-effective, and accurate. This review evaluates the diagnostic accuracy of nucleic acid amplification tests (NAATs), comparing them to the Kato-Katz (KK) and flotation methods for the detection of ascariasis, trichuriasis, and hookworm infection, the Baermann technique (BT) for strongyloidiasis, the Scotch tape test for enterobiasis, and a composite reference standard (CRS).

**Methods:**

We systematically searched PubMed, CINAHL, Scopus, Trip, Web of Science, Cochrane Library, and the academic search engine Google Scholar for studies published within the 12 years preceding September 2024. After the title, abstract and full-text screening, the selected studies were assessed for their methodological quality using Quality Assessment of Diagnostic Accuracy Studies - Version 2 (QUADAS-2). Data were extracted into 2x2 contingency tables, and sensitivity and specificity were pooled using the Reitsma bivariate random-effects model. Forest plots and summary ROC curves were used to explore heterogeneity.

**Principal findings:**

Of the 3,239 articles screened, 35 met the inclusion criteria. Overall, NAATs showed higher pooled sensitivity for HINIs. For *Ascaris lumbricoides*, NAATs showed markedly higher sensitivities of 96–98% against the CRSs, compared with KK and flotation methods (57–67%). For *Trichuris trichiura*, NAAT sensitivity ranged from 74 to 87% across CRSs, whereas KK and flotation exhibited slightly lower but comparable sensitivities (70–83%). For hookworm, NAATs achieved sensitivities of 88–95% against CRS, substantially exceeding those of KK (43%) and flotation (59%) against CRS, with specificities above 87%. In detecting *Strongyloides stercoralis*, NAATs showed 80% sensitivity versus the BT, increasing to 93% against CRS, while the BT showed a sensitivity of 59%. When all soil-transmitted helminths were analysed collectively, pooled sensitivities of NAATs (75–84%) exceeded those of KK (64%), with consistently high specificity across all diagnostic methods. For hookworm, NAATs detected approximately two to threefold more infections than KK and flotation methods, when evaluated against a CRS, highlighting the substantial under-detection by conventional microscopy.

**Conclusion:**

NAATs provide markedly higher sensitivity than copro-microscopy, especially for low-intensity or post-MDA infections. Combining routine microscopy with targeted NAAT deployment and emerging low-cost molecular approaches can optimise diagnostic accuracy and surveillance feasibility, strengthening control programmes and accelerating progress toward the WHO 2030 deworming and elimination goals.

## Author summary

Human intestinal nematode infections continue to affect millions of people, particularly in low- and middle-income countries. Accurate diagnosis is critical for treatment, control, and elimination programs. Traditionally, infections are detected through microscopic examination of stool samples using methods like wet smear, sedimentation methods, flotation methods, Kato-Katz, and the Baermann technique. However, these methods may miss infections, particularly when the infection intensity is low. In recent years, molecular tests such as nucleic acid amplification tests (NAATs) have emerged as a promising alternative, offering higher sensitivity. In this systematic review, we compared NAATs with conventional microscopy for detecting *Ascaris lumbricoides*, *Trichuris trichiura*, hookworm, and *Strongyloides stercoralis*. NAATs consistently showed higher sensitivity, particularly against composite reference standards and for low-intensity infections. Evidence for *Enterobius vermicularis* remained limited, with no studies comparing NAATs to the Scotch tape test. Our findings support integrating NAATs into surveillance, especially in settings approaching elimination, to improve case detection and strengthen control efforts. Using microscopy for routine diagnosis, together with targeted use of NAATs with emerging low-cost molecular tools, provides a feasible approach to strengthen diagnostic capacity and support progress toward the WHO 2030 goals for soil-transmitted helminthiases control programmes.

## Introduction

Human intestinal nematode infections (HINIs) are a common public health problem in many parts of the world, particularly in low- and middle-income countries [1]. It is estimated that over 1.5 billion people are infected globally, accounting for 5.2 million disability-adjusted life years [2]. These infections are caused by *Ascaris lumbricoides*, *Trichuris trichiura, Necator americanus*, *Ancylostoma* species, *Strongyloides stercoralis* and *Enterobius vermicularis* [3,4]. Transmission occurs through contaminated soil, food and water or by direct skin penetration of infective larvae, due to poor sanitation and hygienic practices [1,5]. The morbidity associated with HINIs is particularly severe among children, who are more likely to suffer from heavy worm burdens [6], resulting in impaired cognitive development and growth, malnutrition, anaemia and decreased productivity [7]. The socioeconomic impact of HINIs is profound, perpetuating cycles of poverty and hindering economic development in endemic regions [8].

Detecting HINIs involves several diagnostic methods, each with its advantages and limitations. Microscopy-based techniques, such as direct wet smears (DWS), the Kato-Katz (KK) method, the formalin-ether concentration technique (FECT), flotation methods and culture methods are simple and widely used [9]. The World Health Organization (WHO) recommends the KK method as the gold standard for detecting soil-transmitted helminth (STH) infections in community surveys [10,11]. Its simplicity, affordability, and capacity to estimate infection intensity make it valuable for monitoring and guiding mass drug administration (MDA) programmes [10,11]. Despite these advantages, the method has notable limitations, including low sensitivity for detecting light-intensity infections, particularly hookworm, due to rapid egg degeneration and its inability to provide species-level identification. It is also unsuitable for detecting *Strongyloides* infections, as the method targets eggs rather than larvae, which constitute the diagnostic stage of this parasite [9].

Flotation methods use high-specific-gravity solutions to concentrate parasite eggs, with common techniques including the McMaster, FLOTAC, and Mini-FLOTAC methods [12]. These approaches generally offer higher sensitivity by using concentrated preparations and larger stool volumes, while allowing quantification of STH infection intensity. The FLOTAC technique, in particular, enables rapid processing of large sample numbers and was designed to combine high sensitivity with relatively low cost [13,14]. Nevertheless, hookworm eggs often distort in high-density solutions, and reported sensitivities vary across studies [15]. They are also ineffective for diagnosing *Strongyloides* infections, since the parasite primarily sheds larvae rather than eggs, which are not recovered by flotation techniques [15].

The Baermann technique (BT) detects the larval stages of *S. stercoralis* and hookworms [16]. It is sensitive for identifying live, motile larvae and requires simple lab equipment, making it accessible in low-resource settings [17]. However, it is time-consuming, labour-intensive and requires fresh samples. Methods like agar plate culture (APC) and the Harada Mori technique (HMT) are also useful for detecting larvae of specific HINIs, but are similarly time-consuming, taking 2–10 days [9]. Further, poor standardisation between techniques makes comparison between studies difficult.

The Graham’s Scotch tape method is a simple, non-invasive microscopic technique for detecting *E. vermicularis* eggs. It is cost-effective and has high sensitivity when performed on consecutive days. However, its effectiveness depends on proper timing and technique, as the eggs are present in the early morning. This method is specific to *Enterobius* and is not useful for detecting other HINIs.

Despite the availability of various diagnostic methods, several challenges persist in the accurate diagnosis of HINIs. They often exhibit focal distribution within communities, making it difficult to obtain representative samples for surveillance and diagnosis. In areas with ongoing MDA programs, the intensity of infections tends to be low, reducing the sensitivity of traditional microscopic methods [18]. The decision to implement MDA relies on surveillance results. Reliance on less sensitive diagnostic methods poses significant challenges for STH control programs. By missing light-intensity infections, these tools can underestimate true prevalence and create a false impression that transmission has fallen below critical thresholds [19]. This may lead to premature cessation of MDA, leaving infected individuals untreated and capable of sustaining transmission and long-term morbidity [20]. Evidence from post-MDA surveillance for lymphatic filariasis indicates that STH infections can resurge following the cessation of albendazole administration [21], highlighting how less-sensitive diagnostics can compromise both individual health and elimination goals. Similarly, the re-emergence of schistosomiasis in Sichuan, China, despite years of control, demonstrates how environmental changes, population movement, and inadequate long-term surveillance can allow helminth transmission to rebound once control efforts wane, emphasising the need for sustained and sensitive monitoring tools [22]. Co-infections with multiple human intestinal nematodes and other parasitic diseases complicate diagnosis and require methods capable of detecting and differentiating between species [18,20]. In many endemic regions, limited access to diagnostic facilities, trained personnel, and financial constraints hampers effective diagnosis and control efforts [20]. The choice of diagnostic method depends on available resources, the required sensitivity and specificity, and the context, such as clinical diagnosis or epidemiological surveys, with a combination of methods often enhancing diagnostic accuracy.

In this context, nucleic acid amplification tests (NAATs) have emerged as promising alternatives to conventional microscopy-based methods. These molecular methods detect and amplify parasite-specific nucleic acid sequences, and include polymerase chain reaction (PCR)-based assays, such as conventional PCR (cPCR), nested PCR, real-time quantitative PCR (qPCR), digital PCR (dPCR) [23–25], and other modified PCR formats, as well as isothermal amplification techniques, including loop-mediated isothermal amplification (LAMP), recombinase polymerase amplification (RPA), nucleic acid sequence–based amplification (NASBA), strand displacement amplification (SDA), strand invasion–based amplification (SIBA), and multiple displacement amplification (MLDA) [26]. Each of these isothermal methods leverages different enzymatic processes to achieve amplification using a single temperature, enhancing their specificity, sensitivity, and adaptability for various diagnostic applications [27,28]. By eliminating the need for microscopic identification, NAATs offer the potential for improved sensitivity, species discrimination, and applicability in low-intensity infection settings.

Although numerous studies have evaluated the diagnostic test accuracy (DTA) of individual microscopic methods, no comprehensive review has systematically assessed the accuracy of NAATs for detecting HINIs relative to WHO-recommended reference standards. This review aimed 1) To evaluate existing evidence on the accuracy of NAATs, including PCR and isothermal amplification techniques, providing pooled accuracy estimates, 2) To compare them to microscopy across different transmission settings, and 3) To inform optimal diagnostic strategies for control and elimination programs. This provides useful information for clinicians, public health officials, and policymakers on their utility while identifying knowledge gaps and research needs. By evaluating the diagnostic accuracy of NAATs across populations, the review provides insights into their effectiveness, particularly in low-intensity infection areas where conventional microscopy is less sensitive and fails to accurately detect infections. In addition, understanding the performance of NAATs supports the development of integrated disease management strategies that combine accurate diagnosis with effective treatment and prevention measures.

## Methods

The methodological approach to evidence searching and synthesis followed the Joanna Briggs Institute (JBI) guidelines on systematic reviews of diagnostic test accuracy [29]. In reporting the findings of this review, standards of the Preferred Reporting Items for Systematic Reviews and Meta-Analyses (PRISMA) were adhered to [30]. A protocol describing the detailed methods of this systematic review has been published [31], and the review is registered in PROSPERO (CRD42022315730).

### Eligibility criteria

#### Inclusion criteria.

**Diagnosis of interest:** Detection of HINIs (ascariasis, trichuriasis, hookworm infection, strongyloidiasis, and enterobiasis) using human stool samples.

**Population:** Studies conducted across diverse healthcare settings were included, from community-based to primary, secondary, and tertiary care facilities, as well as studies involving populations from various geographical regions and socioeconomic backgrounds. Asymptomatic and symptomatic individuals were included without any disease severity restrictions. Additionally, there were no limitations regarding sex, age group, ethnicity, or country of origin. Participants with varying immunity statuses, including both immunocompetent and immunocompromised individuals, were also included in the analysis. Only studies conducted on human subjects were included.

**The index tests:** NAAT: PCR, including cPCR, nested PCR, qPCR, dPCR and isothermal amplification assays, including LAMP, NASBA, SDA, RPA, SIBA and MLDA.

**The reference tests:** The KK and flotation methods were used separately as reference tests for *A. lumbricoides, T. trichiura*, and hookworm infections [9,11]. Graham’s Scotch tape test was employed for *E. vermicularis*, while the BT was used as the reference test for identifying *S. stercoralis* infections [9,11].

A composite reference standard (CRS) was employed, combining NAATs with the respective reference tests for each infection.

**Outcomes:** The sensitivity, specificity, positive predictive value (PPV), negative predictive value (NPV) and accuracy of the index tests were the major outcomes.

#### Exclusion criteria.

Studies excluded from this review are case studies, commentaries, and expert opinions, as these formats lack the robust design necessary for assessing diagnostic accuracy. Additionally, studies that did not directly evaluate the diagnostic performance of the index tests were omitted, as were studies that lacked sufficient data to construct a standard two-by-two table required for calculating accuracy metrics.

### Search strategy

A comprehensive literature search was performed in online databases, including PubMed, CINAHL, Scopus, Trip, Web of Science, Cochrane Library, and the academic search engine Google Scholar, for studies published from January 2013 to September 2024, using the following search terms. The search terms and the string used in the PubMed search was (((“ascaris”[All Fields] OR “roundworm”[All Fields] OR “necator”[All Fields] OR “ancylostoma”[All Fields] OR “hookworm”[All Fields] OR “strongyloides”[All Fields] OR “threadworm”[All Fields] OR “trichuris”[All Fields] OR “whipworm”[All Fields] OR “enterobius”[All Fields] OR “pinworm”[All Fields] OR “soil transmitted helminth”[All Fields] OR “geohelminth”[All Fields] OR “intestinal nematode”[All Fields]) AND (“diagnos*”[All Fields] OR “diagnosis”[All Fields] OR “detect”[All Fields] OR “screen”[All Fields] OR “investigat*”[All Fields] OR “investigation”[All Fields] OR “polymerase chain reaction”[All Fields] OR “PCR”[All Fields] OR “molecular”[All Fields] OR “nucleic acid amplification”[All Fields] OR “NAAT”[All Fields] OR “isothermal amplification”[All Fields] OR “loop mediated isothermal amplification”[All Fields] OR “LAMP”[All Fields] OR “microscopy”[All Fields] OR “microscop*”[All Fields] OR “kato katz”[All Fields] OR “baermann technique”[All Fields] OR “scotch tape”[All Fields] OR “flotation”[All Fields] OR “flotac”[All Fields] OR “miniflotac”[All Fields] OR “mcmasters”[All Fields] OR “flot*”[All Fields]))) Filters: Humans. Search terms used in other databases are provided in Table A in the S1 Appendix.

### Study selection

All selected articles were imported into Rayyan for screening (https://www.rayyan.ai) [32]. Duplicates were removed using the platform. Two reviewers (NJ and KW) independently screened the remaining articles based on titles and abstracts, assessing them against the established eligibility criteria. Any discrepancies at this stage led to the inclusion of those articles for further full-text review. The same reviewers independently conducted the full-text screening, and any discrepancies were resolved through the mediation of a third reviewer (CG). The reasons for excluding any full-text articles were documented (Table B in S1 Appendix), and the process is illustrated in the PRISMA flowchart (S1 File).

### Assessment of methodological quality

Two reviewers (NJ and KW) independently evaluated the risk of bias in each included article and reported it according to the Quality Assessment of Diagnostic Accuracy Studies (QUADAS-2) tool [33]. Discrepancies that occurred during the process were resolved by the opinion of a third reviewer (CG). QUADAS-2 includes the risk of bias assessments over four key areas: the patient selection, the index test, the reference standard, and assessment flow and timing. The results of the quality assessment are shown in S1 Table, Figs A and B in S2 Appendix. All the studies reported their design. The majority of them were cross-sectional studies. Most studies used random sampling, while others included all individuals in the defined study population. Nine studies did not provide sufficient details about their exclusion criteria (S1 Data). Three studies included only subjects who tested positive on the reference test, together with a similar number of negative participants. While most studies described the threshold or criteria used to define positivity for the index test, a large number did not report whether examiners were blinded to the results of the index and reference tests. Other aspects of the index and reference tests were sufficiently explained across the studies (S1 Data). None of the studies demonstrated flow and timing issues or applicability concerns.

### Data extraction

A data extraction form was developed using the JBI data extraction instrument as guidance, with modifications relevant to the review (S1 Data). Two reviewers (NJ and KW) retrieved information individually using the customised data extraction form. To ensure consistency, the data extraction protocol was tested on the first ten articles.

Extracted data fell into the following domains (S1 Data):

**Study identification details**: Authors, year of publication, country
**Study methodological details:** Sample size, study design, clinical setting, diagnostic methods assessed, parasite species studied, assessment of co-infection, and any other relevant details like interventions carried out, if any.
**Population characteristics:** Socio-demographic variables (ethnicity, sex, age, religion, marital status, education, employment, income, migration history, household details), history of deworming.
**Index test (NAATs) characteristics:** The type of test, target selection, procedure of sample storage and DNA extraction, time duration between processing and analysis, quality control measures applied, output variables produced and the cost of the test.
**The reference test:** The procedure of the KK test and flotation methods, i.e., number of stool samples taken from a subject, duration between each stool sample collection, number of smears examined from each stool sample, modifications that were done to the standard protocol, details of the modifications, and quality control measures that were applied during the procedure, were extracted from the selected primary studies. BT was used as the reference test for strongyloidiasis, and Graham’s Scotch tape test for enterobiasis. For both methods, data extraction focused on the procedural details, quality control measures, and the number of samples and smears examined.
**Outcome measures:** True positives (TP), false positives (FP), true negatives (TN), and false negatives (FN), sensitivity, specificity, NPV, PPV and accuracy.

### Statistical analyses and results synthesis

Each index test was compared against a reference test. For each test, TP, TN, FP, and FN were retrieved. The sensitivity, specificity, PPV, NPV and accuracy of each study were presented with 95% confidence intervals (CI) and are shown in forest plots. Review manager software (RevMan web and RevMan 5) [34], and R (version 4.4.2) were used to perform this meta-analysis.

The diagnostic accuracy of NAATs for detecting HINIs and individual species was assessed using the KK test, flotation methods, BT and Scotch tape test as the reference standard. Additionally, the diagnostic accuracy of KK, flotation methods, BT and NAATs was evaluated against a CRS. Summary receiver operating characteristic (sROC) curves and forest plots were used to descriptively compare diagnostic performance across studies. These were generated for illustrative purposes and were not based on fitting a specific bivariate meta-analytic model; therefore, specific goodness-of-fit statistics were not calculated. The consistency of summary estimates with individual study results was assessed qualitatively through visual inspection and heterogeneity measures.

The CRS was determined by combining the index and reference test results. Any sample positive on either test was considered positive, and the total positives across both tests were reported as the composite positive rate. Heterogeneity was assessed visually by examining the overlap of confidence intervals and the distribution of study estimates in forest plots, as well as the scatter of points around the summary line in sROC plots. Statistical heterogeneity was quantified using the I^2^ statistic and Cochran’s Q test, which measures the proportion of total variation due to heterogeneity rather than chance. When interventions such as deworming were conducted between two sample collections, diagnostic accuracy was assessed based on the first sample analysis. Studies that evaluated four smears from a single stool sample, as well as those that analysed two smears from two separate samples, were both considered as using quadruplicate KK.

## Results

Following a comprehensive systematic search, 3,239 unique studies were retained for title and abstract screening. Of these, 3,169 were excluded as they did not address the review question. The full texts of the remaining 73 studies were assessed, and 35 studies met the eligibility criteria and were included in the review (S1 File).

### Characteristics of review studies

Of the 38 studies that were excluded after full-text screening, 26 (68.4%) failed to report all relevant outcomes, seven (18.4%) did not provide adequate data for standard outcome analysis, and five (13.1%) were based on a dataset already included in another study considered in this analysis (S1 File, and Table B in S1 Appendix). The included studies were conducted across diverse regions, including South Asia (Bangladesh and India) and Southeast Asia (Lao People’s Democratic Republic, Myanmar, Philippines, Indonesia, Cambodia, Thailand and Timor-Leste), Africa (Ethiopia, Côte d’Ivoire, Kenya, Tanzania, Mozambique, Ghana, and Angola), South America (Argentina and Peru), North America (United States of America) and Oceania (Fiji), involving a total of 20,053 participants (Table 1). Of these studies, 25 (71.4%) used random sampling methods, six (17.1%) included all participants, one (2.8%) used convenience sampling, and three (8.6%) did not specify the method of participant enrollment. All studies were conducted in community settings, except for two that were conducted in a hospital setting. The majority of the studies (n = 34, 97.1%) used PCR as the index test, while one study (2.8%) employed LAMP as the index test. The study participants included either children (n = 15, 42.8%), adults (n = 1, 2.8%), or both children and adults (n = 17, 48.6%). One study (2.8%) specifically focused on pregnant women, and another did not specify the age group of the participants. Of the included studies, 23 (65.7%) evaluated the diagnostic accuracy for *A. lumbricoides*, 22 (62.8%) for *T. trichiura*, 28 (80%) for hookworms, and 12 (34.3%) for *S. stercoralis*.

**Table 1:** Summary of the included studies.

Reference	Country	Setting	Study design	Sample size*	Sampling technique	Population	Species tested	Index test	Reference standard
Schär et al. (2013) [35]	Cambodia	CS	CSS	218	R	Children	Na, Ad, Ss	qPCR	KK and BT
Mens et al. (2013) [36]	Ghana	CS	DAS nested within CSS	195	NR (all included)	Children	Na, Ad	qPCR	KK
Inpankaew et al. (2014) [37]	Cambodia	CS	CSS	205	R	Children and adults	Na, Ad	cPCR	KK and SNF
Knopp et al. (2014) [38]	Tanzania	CS	CSS	215#	NR (all included)	Children and adults	Al, Na, Ss	qPCR	KK, FLOTAC, and BT
Becker et al. (2015) [39]	Côte d’Ivoire	CS	CSS	256	R	Children and adults	Na, Ad, Ss	qPCR	KK and BT
Easton et al. (2016) [40]	Kenya,	CS	CSS	796	R	Children and adults	Al, Tt, Na, Ad, Ss	qPCR	KK
Amor et al. (2016) [41]	Ethiopia	CS	CSS	396	R	Children	Al, Tt, Hw, Ss	qPCR	BT
Llewellyn et al. (2016) [42]	Timor-Leste and Cambodia	CS	Controlled clinical trial	680	R	Children and adults	Al, Tt, Hw	qPCR	SNF
Meurs et al. (2017) [43]	Mozambique	CS	CSS	303	R	Children and adults	Al, Tt, Na, Ad, Ss	qPCR	KK and BT
Mationg et al. (2017) [44]	Philippines	CS	CSS	263	R	Children	Al, Tt, Na, Ad, Ac, Ss	qPCR	KK
Barda et al. (2018) [45]	Laos People’s Democratic Republic	CS	RCT	95	R	Children and adults	Ss	qPCR	BT
Kristanti et al. (2018) [46]	Indonesia	CS	DAS nested within CSS	80	Not stated	Not stated	Ss	cPCR	BT
Clarke et al. (2018) [47]	Timor-Leste	CS	CSS	865	R	Children	As, Tt, Hw	qPCR	SNF
Vlaminck et al. (2019) [48]	Ethiopia, Laos People’s Democratic Republic, and Tanzania	CS	Drug efficacy trial	645	R	Children	Al, Tt, Hw	qPCR	KK and Mini-FLOTAC
Ngari et al. (2020) [49]	Kenya	CS	Diagnostic test validation	137	Not stated	Children	Al, Tt, Hw	LAMP	KK
Dunn et al. (2020) [50]	Myanmar	CS	CSS	648	R	Children and adults	Al, Tt, Na, Ad, Ac	qPCR	KK
Chung et al. (2020) [51]	Bangladesh	CS	CSS	2799	R	Children	Al, Tt, Na, Ad, Ss	qPCR	KK
Chankongsin et al. (2020) [52]	Laos People’s Democratic Republic	HS	CSS	104	NR (consecutive)	Adults	Ss	qPCR	BT
Adisakwattana et al. (2020) [53]	Thailand	CS	CSS	567	NR (all included)	Children and adults	Al, Tt, Na, Ad, Ss	qPCR	KK
Keller et al. (2020) [54]	Pemba Island, Tanzania	CS	CSS within RCT	1636	R	Children and adults	Al, Tt, Na, Ad	qPCR	KK
Amor et al. (2020) [55]	Ethiopia	CS	CSS	792	R	Children and adults	Al, Tt, Hw, Ss	qPCR	BT
Fleitas et al. (2021) [56]	Argentina	CS	CSS	151	Not stated	Children and adults	Na, Ad, Ss	cPCR	KK, McMasters and BT
Azzopardi et al. (2021) [57]	Fiji	CS	RCT	40	R	Children and adults	Al, Tt, Na, Ad, Ac, Ss	qPCR	KK
Pujol et al. (2021) [58]	Mozambique	CS	Regular sampling design	792	R	Children and adults	Al, Tt, Na, Ad, Ac, Ss	qPCR	KK
Bradbury et al. (2021) [59]	USA	HS	CSS	625	Convenience sampling	Children and adults	Al, Tt, Na, Ad, Ss	qPCR	SNF
Bartlett et al. (2021) [60]	Timor-Leste	CS	CSS	478	R	Children	Al, Tt, Na, Ad, Ac, Ss	qPCR	Mini-FLOTAC
Hailu et al. (2022) [61]	Ethiopia	CS	CSS	844	R	Children	Ss	qPCR	BT
Aung et al. (2022) [62]	Myanmar	CS	CSS	264	R	Children	Al, Tt, Na, Ad, Ac	qPCR	KK
Servián et al. (2022) [63]	Argentina	CS	CSS	140	R	Children and adults	Na, Ad	qPCR	SNF
Noor et al. (2023) [64]	Bangladesh	CS	CSS	386	NR (All included)	Children	Al, Tt, Na, Ad	qPCR	KK
Poole et al. (2023) [65]	USA	CS	CSS	625	R	Children	Al. Tt, Na, Ad, Ss,Ev	qPCR and dPCR	Mini-FLOTAC
Soultani et al. (2024) [66]	Angola	CS	CSS	2974	R	Children	Al, Tt, Na, Ad	qPCR	KK
Gaidhane et al. (2024) [67]	India	CS	CSS	534	R	Pregnant mothers	Al, Tt, Na, Ad	qPCR	KK
Malaga et al. (2024) [68]	Peru	CS	CSS	300	NR (All included)	Children and adults	Ss	qPCR	BT
Mugo et al. (2024) [69]	Kenya	CS	CSS	390	R	Children	Al, Tt, Hw, Ss	qPCR	KK, Mini-FLOTAC and BT

CS, community setting; HS, hospital setting; CSS, cross-sectional study; DAS, diagnostic accuracy study; RCT, randomized control trials; R, randomized; NR, non-randomized; Al, *Ascaris lumbricoides*; Tt, *Trichuris trihiura*; Na, *Necator americanus*; Ad, *Ancylostoma duodenale*; Ac, *Ancylostoma ceylanicum*; Ss, *Strongyloides stercoralis*; Hw, Hookworm; qPCR, real-time polymerase chain reaction; cPCR, conventional polymerase chain reaction; dPCR, digital polymerase chain reaction; LAMP, loop mediated isothermal amplificatiom; KK, Kato-Katz; SNF, sodium nitrate flotation; BT, Baermann test.

* Sample size represents the number of participants who underwent both the index and reference tests.

# In this study, the sample sizes analysed were 215 for the comparison between PCR and KK, 193 for PCR and the BT, and 213 for PCR and FLOTAC.

Across all test comparisons, moderate to high heterogeneity was observed for sensitivity estimates (54.8% to 97.9%) and specificity (0–99%), indicating considerable variability in diagnostic performance among studies. Individual I² values for each analysis are presented in the respective figures and supplementary forest plots. Given the limited number of studies within each comparison, further subgroup analyses were not conducted, as they would lack statistical power and validity to reliably assess variability in results.

### Detection of ascariasis

A total of 15 studies have assessed ascariasis prevalence using both NAATs and KK (Fig C in S2 Appendix). Of those, the majority of the studies (n = 7, 46.7%) compared the accuracy of qPCR with duplicate KK tests, while others compared qPCR with single (n = 3, 20%), triplicate (n = 3, 20%), and quadruplicate (n = 2, 13.3%) KK tests. One study [51] was excluded from the meta-analysis, as its authors suspected that KK-positive PCR-negative results were due to the misidentification of pollen or other artefacts as *Ascaris* eggs. Nine studies (60%) incorporated a mechanical breakdown step during DNA extraction (Table 2). Except for one study [54] that did not specify the gene target, all others used the ITS1 gene for PCR amplification. Variations were noted in the number of replicates, sample storage methods, DNA extraction kits, cycling conditions and cutoff values (Table 2).

**Table 2:** Index test (NAAT) characteristics of included studies.

Reference	Diagnostic method		Sample No.	Replicate No*	Storage method	DNA extraction details		Gene targets	Blinding	Threshold level	QC methods applied
						Chemical extraction	Mechanical breakdown				
Chankongsin et al. (2020) [52]	qPCR	SP	1	1	90% ethanol at room temperature	QIAamp DNA Stool Mini Kit	Not used	28S rRNA gene of *Strongyloides* spp.	Not stated	One plasmid copy/μl DNA	Yes
Hailu et al. (2022) [61]	qPCR	SP	1	Not stated	No storage done(fresh stool used)	QIAamp DNA Stool Mini Kit	Not used	18S rRNA small subunits of *S. stercoralis*	DB	Positivity based on amplification, and a correct melting curve profile	Yes
Becker et al. (2015) [39]	qPCR	DP and SP	1	1	70% ethanol at room temperature	QIAamp DNA Stool Mini Kit	Not used	18S rRNA small subunits of *S. stercoralis* ITS-2 region of the ribosomal DNA for hookworm species	Not stated	No predefined cutoff; positivity based on amplification signal with Ct recorded	Yes
Fleitas et al. (2021) [56]	cPCR	MP	1	1	No storage done (fresh stool used)	FastPrep Spin Kit for Soil	Not used	18S rRNA small subunits of *S. stercoralis* ITS-2 region of the ribosomal DNA for hookworm species	SB	Correct-size band on agarose gel; sequencing confirmed in subset	Yes
Azzopardi et al. (2021) [57]	qPCR	MP	1	2	2.5% (w/v) potassium dichromate at room temperature	Isolate II Fecal DNA Kit	Bead beating	18S rRNA gene for *S. stercoralis* and *T. trichiura* ITS 1 for *A. lumbricoides*, *A. duodenale*, and *A. ceylanicum*, ITS 2 for *N. americanus*	Not stated	A sample was considered positive if either qPCR reaction had a Cq < 37	Yes
Ngari et al. (2020) [49]	LAMP	SP	1	Not stated	Fresh stool used	QIAamp DNA Stool Mini Kit	Not used	ITS-2 ribosomal DNA of *T. trichiura*	Not stated	Amplicon positivity was judged by gel (band presence) or by colour change with SYBR green	Yes
Knopp et al. (2014) [38]	qPCR	MP	1	Not stated	−80 °C without preservative	QIAamp Tissue Kit	Not used	ITS1 for *A. lumbricoides* ITS2 for *N. americanus* 18S rRNA gene for *S. stercoralis*	Not stated	Ct ≤ 40 was considered positive	Yes
Dunn et al. (2020) [50]	qPCR	MP	1	2	−80 °C without preservative	MP Bio Fast DNA Spin Kit for Soil	Bead beating	ITS1 for *A. lumbricoides* and *T. trichiura*, *Ancylostoma* spp. ITS 2 for *N. americanus*	Not stated	Amplification before the cycle threshold and the internal control was considered positive	Yes
Chung et al. (2020) [51]	qPCR	MP	1	2	100% ethanol and stored at -80 °C	Fast DNA Spin Kit for Soil	Bead beating	ITS1 for *A. lumbricoides* and *T. trichiura*, *Ancylostoma* spp. ITS 2 for *N. americanus*	DB	Positive if amplification occurred in both replicate reactions with a Cq < 40	Not stated
Meurs et al. (2017) [43]	qPCR	MP	1	Not stated	96% ethanol and stored at -20 °C	QIAamp DNA Stool Mini Kit	Not used	ITS1 for *A. lumbricoides*, *T. trichiura*, and *Ancylostoma* spp. ITS 2 for *N. americanus* 18S rRNA gene for *S. stercoralis*	Not stated	Amplification before the cycle threshold of 50 was considered positive	Yes
Mationg et al. (2017) [44]	qPCR	MP and SP	1	Not stated	80v/v ethanol and stored at 4 °C	Maxwell 16 LEV Plant DNA Kit	Bead beating and thermal disruption	ITS1 for *A. lumbricoides* and *T. trichiura* ITS 2 for Ancylostoma spp. and *N. americanus* 18S rRNA gene of *S. stercoralis*	Not stated	Normal melt curve and comparative quantification analysis were considered positive	Yes
Barda et al. (2016) [45]	qPCR	SP	2	Not stated	Preserved in ethanol	QIAamp DNA Stool Mini Kit	Not used	18S rRNA gene of *S. stercoralis*	Not stated	A Ct value ≤ 40 was considered positive	Yes
Schär et al. (2013) [35]	qPCR	SP	1	Not stated	−20 °C without preservative	QIAmp DNA Stool Mini Kit	Not used	ITS 1 for *Ancylostoma* spp. ITS 2 for *N. americanus* 18S rRNA gene of *S. stercoralis*	Not stated	Not stated	Not stated
Mens et al. (2013) [36]	qPCR	MP	1	3	−20 °C without preservative	QIAamp DNA Stool Mini Kit	Not stated	ITS 1 for *Ancylostoma* spp. ITS 2 for *N. americanus*	Not stated	Not stated	Not stated
Inpankaew et al. (2014) [37]	cPCR	SP	2	Not stated	Potassium dichromate	PowerSoil DNA Kit	Bead beating	ITS-1, 5.8S and ITS-2 region of hookworms	Not stated	Correct band visualised on 1% agarose gels stained with SYBR Safe was considered positive	Yes
Easton et al. (2016) [40]	qPCR	SP with NGS	1	4	−15 °C without preservative	PowerSoil and PowerMag Kits	Bead beating	ITS1 for *A. lumbricoides* and *T. trichiura* ITS 2 for *Ancylostoma* spp. and *N. americanus*	DB	Ct < 40 was considered positive	Yes
Pujol et al. (2021) [58]	qPCR	MP	2	Not stated	−20 °C without preservative	Power Faecal Pro DNA Kit	Not used	ITS 1 for *A. lumbricoides, T. trichiura* and *Ancylostoma* spp. ITS 2 for *N. americanus* 18S rRNA gene of *S. stercoralis*	DB	No Ct cut-off; amplification detected within 50 cycles was considered positive	Yes
Adisakwattana et al. (2020) [53]	qPCR	MP	1	2	80% v/v ethanol	QIAamp Fast DNA Stool Mini Kit	Not used	ITS1 for *A. lumbricoides* and *T. trichiura* ITS 2 for *Ancylostoma* spp. and *N. americanus* 18S rRNA gene of *S. stercoralis*	Not stated	Cq < 37 was considered positive	Yes
Soultani et al. (2024) [66]	qPCR	MP	1	Not stated	96% ethanol	Maxwell RSC PureFood GMO and authentication Kit	Bead beating and thermal disruption	ITS1 for *A. lumbricoides* and *T. trichiura* ITS 2 for *Ancylostoma* spp. and *N. americanus*	Not stated	Ct values converted to eggs per gram using experimentally derived formulae	Yes
Keller et al. (2020) [54]	qPCR	MP	2	3	80% ethanol and preserved at 4 °C	QIAamp DNA Stool Mini Kit	Not used	Not stated	Not stated	Copy numbers above zero were considered positive	Yes
Gaidhane et al. (2024) [67]	qPCR	MP	3	Not stated	80% ethanol	QIAamp DNA Stool Mini Kit	Not used	ITS1 for *A. lumbricoides* and *T. trichiura* ITS 2 for *Ancylostoma* spp. and *N. americanus*	Not stated	Not stated	Yes
Malaga et al. (2024) [68]	qPCR	SP	1	2	70% ethanol	E.Z.N.A.Stool DNA Kit	Beat beating and thermal disruption	*S. stercoralis* 18s rRNA gene	DB	If both reactions showed similar amplification curves, the products had the same melting point (±1 °C) as the positive control, and the non-template control showed no amplification	Yes
Kristanti et al. (2018) [46]	cPCR	SP	1	Not stated	96% ethanol and -20°C	FavorPrep Stool DNA Isolation Mini Kit	Bead beating	*S. stercoralis* ITS2 rDNA	Not stated	Presence of a 114 base pair band on agarose gel was considered positive	Yes
Amor et al. (2016) [41]	qPCR	SP	1	2	4°C without preservative	QIAamp DNA Stool Mini Kit	Not used	*S. stercoralis* 18S ribosomal subunit	Not stated	An exponential amplification curve with a Ct ≤ 38 and a characteristic melting temperature peak	Yes
Llewellyn et al. (2016) [42]	qpCR	MP	1	Not stated	5% w/v potassium dichromate solution at room temperature	Powersoil DNA Isolation Kit	Bead beating	ITS1 for *A. lumbricoides*, *T. trichiura* and *Ancylostoma* spp. ITS 2 for and *N. americanus*	Not stated	Ct ≤ 31 for *Ascaris* spp. And Ct ≤ 35 for other species were considered positive	Yes
Clarke et al. (2018) [47]	qPCR	MP	1	2	5% w/v potassium dichromate and stored at 4°C	PowerSoil DNA Isolation Kit	Bead beating	ITS1 for *A. lumbricoides*, *T. trichiura* and *Ancylostoma* spp. ITS 2 for and *N. americanus*	DB	Ct ≤ 31 for *Ascaris* spp. And Ct ≤ 35 for other species were considered positive	Yes
Vlaminck et al. (2019) [48]	qPCR	MP	2	2	Absolute ethanol	QIAsymphony DSP Virus/Pathogen Midi Kit	Bead beating	ITS1 for *A. lumbricoides* and *T. trichiura* ITS2 for *N. americanus*, and *Ancylostoma* spp.	Not stated	Cq ≤ 35 in duplicate runs	Yes
Amor et al. (2020) [55]	qPCR	SP	1	2	Fresh stool sample used	QIAamp DNA Stool Mini Kit	Not used	*S. stercoralis* 18S ribosomal subunit	DB	Ct < 40 with correct melting temperature	Yes
Bradbury et al. (2021) [59]	qPCR	MP	1	1 (repeat test in positive)	-80 °C	SurePrep Soil DNA Isolation Kit	Bead beating	ITS1 for *A. lumbricoides* and *T. trichiura* ITS2 for *A. duodenale* and *N. americanus* 18S rRNA for *S. stercoralis*	Not stated	Ct < 35 considered positive	Yes
Bartlett et al. (2021) [60]	qPCR	MP	1	2	5% (w/v) potassium dichromate and stored at 4°C	PowerSoil DNA Isolation Kit	Bead beating	ITS1 for *A. lumbricoides*, *T. trichiura* and *A. duodenale* ITS2 for *N. americanus*	Not stated	Ct ≤ 38 considered positive	Yes
Aung et al. (2022) [62]	qPCR	MP	1	3	80% v/v ethanol	Maxwell 16 LEV Plant DNA Kit	Bead beating and thermal disruption	ITS1 for *A. lumbricoides* and *T. trichiura* ITS2 for *N. americanus*, and *Ancylostoma* spp.	Not stated	Ct < 35 is considered positive. Replicate criteria: Positive samples required at least 2 of 3 replicates with SD < 1	Yes
Servián et al. (2022) [63]	cPCR	SP with SS	3 - 5	1	70% ethanol	ZR Fecal DNA MiniPrep Kit	Thermal disruption	Cytochrome b (cob) gene for *N. americanus* ITS1 rRNA region for *A. duodenale*	Not stated	Presence of a species-specific amplicon of the expected size on gel electrophoresis	Yes
Noor et al. (2023) [64]	qPCR	MP	2	Not stated	Not stated	QIAamp Fast DNA Stool Mini Kit	Bead beating and thermal disruption	ITS1 for *A. lumbricoides* 18s rRNA for *T. trichiura* ITS2 for *N. americanus*, and *Ancylostoma* spp.	DB	Cq ≤ 36 were considered positive	Yes
Poole et al. (2023) [65]	qPCR and dPCR	MP	1	1	Zn-PVA preserved	QIAamp 96 Virus QIAcube HT Kit and DNeasy PowerSoil Pro Kit	Bead beating	ITS1 for A. *lumbricoides* and *T. trichiura* ITS2 for *A. duodenale* and *N. americanus* 5S rRNA region for *E vermicularis* 18S rRNA for *S. stercoralis*	DB	qPCR - Cq ≤ 35 were considered positive, and dPCR (QIAcuity): < 3 positive partitions were classified as negative	Yes
Mugo et al. (2024) [69]	qPCR	MP	1	2	Fresh stool sample used	DNeasy 96 PowerSoil Pro QIAcube HT Kit	Bead beating	ITS1 for *A. lumbricoides* and *T. trichiura* ITS2 for *A. duodenale* and *N. americanus* 18S rRNA for *S. stercoralis*	DB	Cq ≤ 35 in duplicate runs	Yes

qPCR; real-time polymerase chain reaction, cPCR; conventional polymerase chain reaction, dPCR; digital polymerase chain reaction, LAMP; loop mediated isothermal amplificatiom SP; single/monoplex, DP; duplex, MP; multiplex, QC; quality control, ITS; Internal transcribed spacer, rRNA; ribosomal ribonucleic acid, rDNA; ribosomal deoxyribonucleic acid, w/v; weight to volume, v/v; volume to volume, Cq; quantification cycle, Ct; cycle threshold, SS; Sanger sequncing, NGS; next generation sequencing, DB; Double blinding.

*In all the studies, the index test was carried out by experienced microscopists.

#### Comparison of NAATs with KK for the detection of ascariasis.

**Fig 1:**
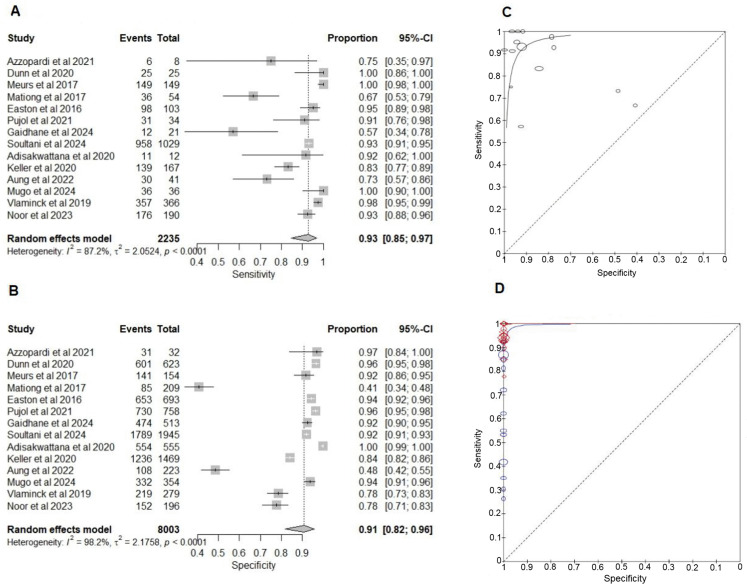
Forest plots and summary receiver operating curves for the diagnostic accuracy meta-analysis for *Ascaris lumbricoides.* **(A)** Forest plot of the sensitivity of nucleic acid amplification tests (NAATs) compared to Kato-Katz (KK). **(B)** Forest plot of the specificity of NAATs compared to KK. **(C)** sROC of NAATs and KK. **(D)** NAATs and composite reference standard (CRS) and KK and CRS. CRS comprises NAAT and KK results. The plots are generated using the Reitsma bivariate model in the mada package in R. Each study’s point estimate and 95% confidence interval are represented by squares and horizontal lines, respectively. The curves are generated using the Review Manager RevMan 5.4.1. The x-axis represents Specificity (False positive rate), and the y-axis represents Sensitivity (True positive rate).

NAATs sensitivity varied from 57% (95% CI: 34%–78%) to 100% (95% CI: 86%–100%), with a pooled estimate of 93% (95% CI: 85%–97%) (Fig 1). The two studies reporting the lowest sensitivities (57% and 67%) showed that qPCR detected more positives than KK, with a lower proportion of samples being positive by both tests, thus reducing the number of TPs in the 2x2 table [44,67]. Specificity ranged from 41% (95% CI: 34%–48%) to 100% (95% CI: 99%–100%), with a pooled estimate of 91% (95% CI: 82%–96%) (Table 3). The two studies with specificities of 41% and 48% similarly reported more PCR positives than KK, reflecting higher FP rates and reduced apparent specificity [44,62]. All studies were community-based and used qPCR assays targeting the ITS1 region, except for one that did not specify the gene target. Variability in sensitivity and specificity likely reflected differences in sample size (40–2,974), preservation methods (70–100% ethanol, potassium dichromate, or storage at –80 °C to 4 °C), DNA extraction kits, and use of a mechanical breakdown step during extraction (Table 2). Overall, qPCR demonstrated high pooled sensitivity (93%, 95% CI: 85%–97%) and specificity (91%, 95% CI: 82%–96%), supporting its reliability for diagnosing ascariasis (Fig 1)

**Table 3:** Summary of diagnostic test accuracy of NAATs, KK and BT for detecting HINIs.

Species	Index test	Reference test	Sensitivity	Specificity
*Ascaris lumbricoides*	NAAT	KK	93% (85%-97%)	91% (82%-96%)
	NAAT	Composite (NAAT+KK)	96% (93%-97%)	100%
	KK	Composite (NAAT+KK)	67% (53%-79%)	100%
	NAAT	Flotation	96% (88%-98%)	97% (79%-100%)
	NAAT	Composite (NAAT+flotation)	98% (92%-99%)	100%
	Flotation	Composite (NAAT+flotation)	57% (34%-77%)	100%
*Trichuris trichiura*	NAAT	KK	74% (47%-90%)	89% (74%-96%)
	NAAT	Composite (NAAT+KK)	85% (67%-94%)	100%
	KK	Composite (NAAT+KK)	70% (53%-80%)	100%
	NAAT	Flotation	82% (47%-96%)	99% (90%-100%)
	NAAT	Composite (NAAT+flotation)	87% (60%-97%)	100%
	Flotation	Composite (NAAT+flotation)	83% (60%-97%)	100%
Hookworm	NAAT	KK	88% (77%-94%)	87% (82%-92%)
	NAAT	Composite (NAAT+KK)	95% (91%-98%)	100%
	KK	Composite (NAAT+KK)	43% (27%-62%)	100%
	NAAT	Flotation	90% (77%-96%)	94% (94%-95%)
	NAAT	Composite (NAAT+flotation)	95% (86%-98%)	100%
	Flotation	Composite (NAAT+flotation)	59% (41%-75%)	100%
*Strongyloides stercoralis*	NAAT	BT	80% (54%-93%)	76% (64%-92%)
	NAAT	Composite (NAAT+BT)	93% (77%-98%)	100%
	BT	Composite (NAAT+BT)	59% (48%-69%)	100%
Soil-transmitted helminths (*Ascaris*, *Trichuris* and hookworm)	NAAT	KK	75% (52%-89%)	71% (38%-90%)
	NAAT	Composite (NAAT+KK)	84% (65%-93%)	100%
	KK	Composite (NAAT+KK)	64% (40%-83%)	100%

NAAT, nucleic acid amplification test; KK, Kato-Katz; BT, Baerman test.

#### Comparison of NAATs and KK with the composite reference for the detection of ascariasis.

Forest plots indicate that sensitivity of NAATs were generally high across all the studies, ranging from 78% (95% CI: 40%–97%) to 100% (95% CI: 98%–100%) with a pooled sensitivity of 96% (95% CI: 93%–97%), indicating a high degree of accuracy (Fig D in S2 Appendix). The sensitivity of KK varies considerably across studies, ranging from 26% (95% CI: 20%–34%) to 92% (95% CI: 64%–100%). The pooled sensitivity was 67% (95% CI: 53%–79%) (Fig D in S2 Appendix). The four studies reporting the lowest sensitivities, 26%, 30%, 35% and 42% [44,54,62,67], used triplicate and quadruplicate KK. In contrast, some studies that utilised a single KK [43] smear reported higher sensitivities, reaching up to 95%. One study with low sensitivity reported that the majority of infected participants (92.3%) had low or moderate infection intensity [67]. The other studies did not provide information on the infection intensity. As a CRS was used, in which any positive result is considered a true infection, both KK and NAATs show 100% specificity (Tables C and D in S1 Appendix).

The sROC curves lie above the diagonal line, indicating that the NAATs and KK tests are superior to the random effect. Comparison between NAATs and composite and KK and composite shows that both tests have an area under the curve (AUC) of 0.9, showing higher overall diagnostic accuracy (Fig 1).

NAATs provide greater sensitivity than KK while maintaining equivalent specificity in identifying ascariasis, irrespective of the infection intensities.

### Detection of trichuriasis

Thirteen studies assessed the prevalence of trichuriasis using both NAAT and the KK method (Fig C in S2 Appendix). qPCR was used as the index test in twelve studies (92.3%), while one study (7.7%) employed the LAMP test. Most studies (n = 6, 46.1%) compared the diagnostic accuracy of qPCR with the duplicate KK test, whereas others compared qPCR with KK performed in single (n = 2, 15.4%), triplicate (n = 3, 23.1%), or quadruplicate (n = 2, 15.4%) replicates.

Most studies targeted the ITS1 gene, whereas two studies used the 18S rRNA gene, and one study targeted the ITS2 gene (Table 2). Sample storage methods varied, with preservation in 70–100% ethanol or potassium dichromate and storage temperatures ranging from − 80 °C to 4 °C, with or without preservatives. Eight studies incorporated a mechanical disruption step during DNA extraction (Table 2). Additional methodological variations were noted in the number of replicates, PCR cycling conditions, and positivity cutoff values (Tables 1 and 2).

#### Comparison of NAATs with KK for the detection of trichuriasis.

**Fig 2:**
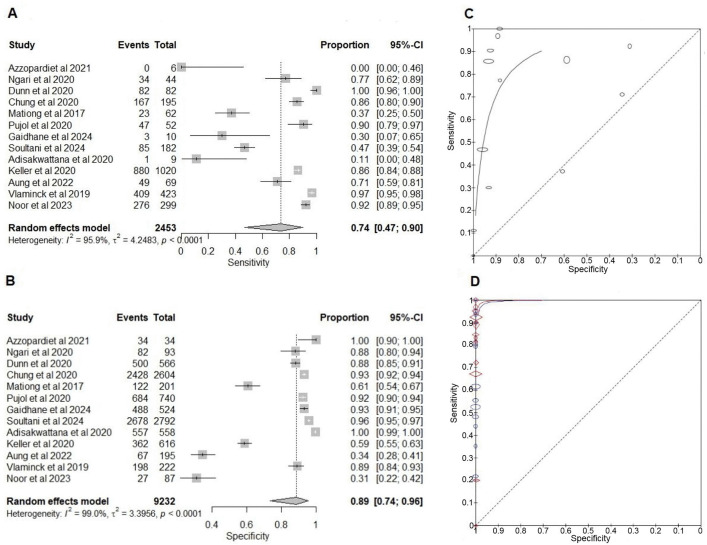
Forest plots and summary receiver operating curves for the diagnostic accuracy meta-analysis for *Trichuris trichiura.* **(A)** Forest plot of the sensitivity of nucleic acid amplification tests (NAATs) compared to Kato-Katz (KK). **(B)** Forest plot of the specificity of NAATs compared to KK. **(C)** sROC of NAATs and KK. **(D)** NAATs and composite reference standard (CRS) and KK and CRS. CRS comprises NAAT and KK results. The plots are generated using the Reitsma bivariate model in the mada package in R. Each study’s point estimate and 95% confidence interval are represented by squares and horizontal lines, respectively. The curves are generated using the Review Manager RevMan 5.4.1. The x-axis represents Specificity (False positive rate), and the y-axis represents Sensitivity (True positive rate).

The sensitivity of NAATs varied widely across studies, ranging from 0% (95% CI: 0%–46%) to 100% (95% CI: 96%–100%). The pooled sensitivity was 74% (95% CI: 47%–90%). Five studies reported sensitivities below 50% (0%, 11%, 30%, 37% and 47%) (Fig 2). The three studies with sensitivities 30%, 37% and 47% showed that PCR detected more positive cases compared to KK [44,66,67]. However, the proportion of samples positive by both tests was low, resulting in a reduced number of TPs. The study that reported 0% sensitivity (Azzopardi et al., 2021) used the 18S rRNA gene target, potassium dichromate as the preservative, Isolate II Fecal DNA Kit, and incorporated a mechanical breakdown step, with a relatively small sample size of 40 [57]. In contrast, the study reporting 11% sensitivity (Adisakwattana et al., 2020) used the ITS1 gene target, 80% ethanol as the preservative, and the QIAamp Fast DNA Stool Mini Kit for DNA extraction without a mechanical breakdown step, and included a larger sample size of 567 [53]. Both studies compared qPCR with duplicate KK. Notably, both studies were conducted in low-endemic settings. All the other studies, except one, used the ITS1 gene target. The majority used ethanol-based preservation with different DNA extraction kits.

Specificity for NAAT also varied widely, ranging from 31% (95% CI: 22%–42%) to 100% (95% CI: 90%–100%). The pooled specificity was 89% (95% CI: 74%–96%). Two studies reported a specificity below 50% (31% and 34%) [62,64]. In both cases, PCR detected a higher number of positives compared to the KK method, with a lower proportion of samples being positive by both tests, thus increasing the number of FPs in the 2x2 table and consequently reducing specificity.

Overall, most studies demonstrated that PCR-based diagnostic tests have high accuracy, with a pooled sensitivity of 74% and specificity of 89%, indicating that NAATs are reliable tools for diagnosing *T. trichiura* infection (Fig 2).

#### Comparison of NAATs and KK with the composite reference for the detection of trichuriasis.

Except for the two studies mentioned above (Azzopardi et al., 2021 and Adisakwattana et al., 2020), all other studies demonstrated relatively high NAAT sensitivity, ranging from 67% (95% CI: 62%–73%) to 100% (95% CI: 98%–100%), with a mean sensitivity of 85% (95% CI: 67%–94%) (Fig E in S2 Appendix) [44,67]. All except one study [49] employed qPCR, predominantly targeting the ITS1 gene. The included studies exhibited substantial variability in infection intensity among study populations, variations in sample preservation methods and storage durations, as well as the DNA extraction protocols employed.

The sensitivity of KK varies significantly across studies, ranging from 22% (95% CI: 11%–36%) to 100% (95% CI: 54%–100%), reflecting the limited ability of KK to detect all TPs. The pooled sensitivity for the KK is 70% (95% CI: 53%–83%), indicating an average sensitivity with a notable range (Fig E in S2 Appendix). The pooled specificity is consistently high at 100%, showing excellent specificity with minimal variability across the studies. sROCs for both KK and NAATs lie above the diagonal line. NAATs demonstrate higher overall diagnostic accuracy. While both tests are useful, NAATs are the more reliable option for accurately detecting TP cases (Fig 2).

In contrast to KK, NAATs show a higher pooled sensitivity, indicating that NAATs are more effective in identifying TP cases of *Trichuris*. Similar to KK, the specificity for NAATs remains consistently high at 100% in all studies. Overall, these findings indicate that NAATs provide greater sensitivity than KK while maintaining equivalent specificity for detecting trichuriasis (Tables E and F in S1 Appendix).

### Detection of hookworm infection

Of the included studies, 18 compared hookworm prevalence using NAATs and KK. All the studies assessed the diagnostic accuracy of NAATs for *N. americanus,* and 17 evaluated *Ancylostoma* spp. Nine studies (n = 9, 50%) assessed the accuracy of qPCR compared to duplicate KK. The remaining studies compared qPCR with single (n = 3, 16.7%), triplicate (n = 3, 16.7%), and quadruplicate (n = 3, 16.7%) KK tests.

The studies used a range of DNA extraction kits and sample storage methods, with the majority (n = 10, 55.6%) including a mechanical breakdown step during DNA extraction (Table 2). All studies used the ITS1 region as the gene target for *N. americanus*, whereas both ITS1 and ITS2 regions were targeted for *Ancylostoma* spp. Variations were noted in the primer sequence, cycling conditions and in cutoff values (Table 2).

#### Comparison of NAATs with the KK for the detection of hookworm infection.

The sensitivities of NAATs varied, ranging from 67% (95% CI: 30%–93%) to 100% (95% CI: 92%–100%) except for two studies [53,64]. Across the studies, NAATs demonstrated a pooled sensitivity of 88% (95% CI: 77%–94%) and a pooled specificity of 87% (95% CI: 82%–92%) (Table 3).

The two studies with the lowest sensitivities (Noor et al., 16% and Adisakwattana et al., 52%) showed that KK detected more hookworm eggs than detected by the qPCR. Both studies used the ITS2 gene target, the QIAamp Fast DNA Stool Mini Kit, and incorporated a mechanical breakdown step, with sample sizes of 386 and 567 [53,64]. In one study [64], stool preservation was not specified, whereas the other used 80% ethanol.

**Fig 3:**
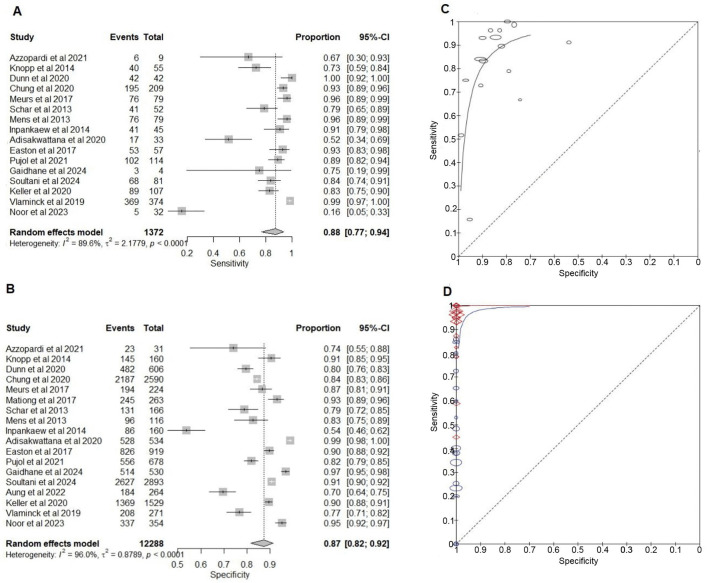
Forest plots and summary receiver operating curves for the diagnostic accuracy meta-analysis for hookworms. **(A)** Forest plot of the sensitivity of nucleic acid amplification tests (NAATs) compared to Kato-Katz (KK). **(B)** Forest plot of the specificity of NAATs compared to KK. **(C)** sROC of NAATs and KK. **(D)** NAATs and composite reference standard (CRS) and KK and CRS. CRS comprises NAAT and KK results. The plots are generated using the Reitsma bivariate model in the mada package in R. Each study’s point estimate and 95% confidence interval are represented by squares and horizontal lines, respectively. The curves are generated using the Review Manager RevMan 5.4.1. The x-axis represents Specificity (False positive rate), and the y-axis represents Sensitivity (True positive rate).

All included studies except one employed qPCR [37], targeting the ITS2 region for *N. americanus* and the ITS1 and ITS2 regions for *Ancylostoma* spp (Table 2). Most studies also used ethanol-based preservation (Fig 3 and Table 2).

Except for one study, specificities of the studies varied between 70% (95% CI: 64%–75%) and 99% (95% CI: 98%–100%) [37]. In this study, PCR detected hookworm infection more than KK, with a lower proportion of samples being positive by both tests, thus increasing the number of FP in the 2x2 table, reducing the specificity.

#### Comparison of NAATs and KK with the composite reference for the detection of hookworm infection.

Except for two studies discussed above (Noor et al., Adisakwattana et al.), NAATs exhibited a higher sensitivity, ranging from 79% (95% CI: 67%–87%) to 100% (95% CI: 98%–100%), with a mean sensitivity of 95% (95% CI: 91%–98%) [53,64], making them more effective at detecting TPs. NAATs also maintain a consistently high specificity of 100% across all studies, with a mean specificity of 100% (Fig F in S2 Appendix, and Table G in S1 Appendix).

The sensitivity of KK varied across studies, ranging from 0% (95% CI: 0%–19%) to 86% (95% CI: 82%–89%), with a mean sensitivity of 43% (95% CI: 27%–62%) (Table 3). Its specificity remains consistently high at 100% with a mean specificity of 100%. Some of the studies with triplicate and quadruplicate KK had lower sensitivity (Mationg et al., triplicate KK, sensitivity 0%), while single and duplicate KK had higher sensitivity (Adisakwattana et al., duplicate KK, sensitivity 100%) compared to the CRS [44,53] (Fig F in S2 Appendix, and Table H in S1 Appendix). sROCs for both KK and NAATs curves lie above the diagonal line with an AUC of 0.99, indicating that both methods have a very good diagnostic value for detecting hookworm infections (Fig 3). However, NAATs show higher overall accuracy, particularly in terms of sensitivity, suggesting NAATs can detect more TP cases than KK. Overall, NAAT demonstrate greater sensitivity than KK for detecting hookworm infections while maintaining equal specificity (Tables G and H in S1 Appendix).

### Detection of strongyloidiasis

A total of 12 studies assessed *Strongyloides* infection in 3,747 participants (Fig C in S2 Appendix). The majority (n = 10, 83.3%%) of the studies compared qPCR with BT, while two (16.7%) studies compared cPCR with BT (Table 1). Ten studies (83.3%) targeted the 18S rRNA gene, while two studies used alternative nuclear markers (28S rRNA and ITS2 rDNA). Half of the studies (50%) analysed stool preserved in ethanol, and the remainder used fresh stool or material stored at temperatures ranging from −20°C to 4°C. Most studies (n = 9, 75%) used the QIAamp DNA Stool Mini Kit for DNA extraction, whereas the others employed different extraction methods (Table 2).

#### Comparison of NAATs and the BT for the detection of strongyloidiasis.

**Fig 4:**
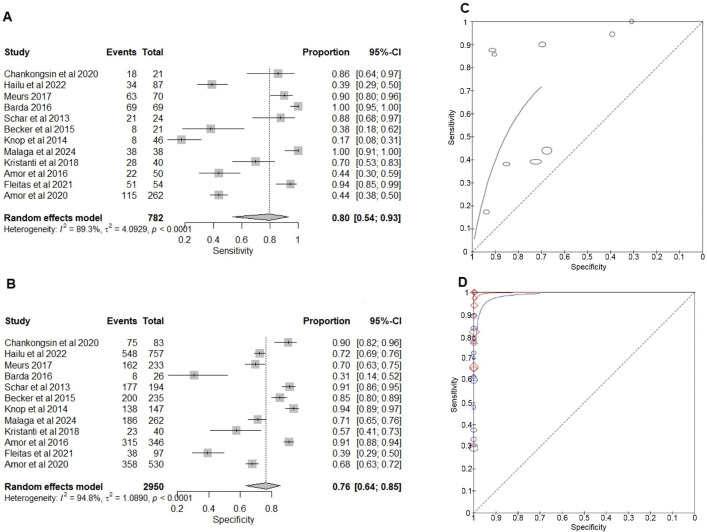
Forest plots and summary receiver operating curves for the diagnostic accuracy meta-analysis for *Strongyloides stercoralis.* **(A)** Forest plot of the sensitivity of nucleic acid amplification tests (NAATs) compared to the Baermann Test (BT). **(B)** Forest plot of the specificity of NAATs compared to the BT. **(C)** sROCs of NAATs and the BT. **(D)** NAATs and Composite and BT and Composite. Composite comprises the NAAT and BT results. The plots are generated using the Reitsma bivariate model in the mada package in R. Each study’s point estimate and 95% confidence interval are represented by squares and horizontal lines, respectively. The curves are generated using the Review Manager RevMan 5.4.1. The x-axis represents Specificity (False positive rate), and the y-axis represents Sensitivity (True positive rate).

The sensitivity of NAATs varied widely across studies from 17% (95% CI: 8%–31%) to 100% (95% CI: 95%–100%) with a pooled sensitivity of 80% (95% CI: 54%–93%). Five studies showed a sensitivity less than 50% (17%, 38%, 39%, 44%, 44%) for NAATs (Fig 4). All except one study showed that PCR detected more positives than the BT, although the agreement between BT and qPCR was relatively low [38]. All five of them employed qPCR targeting the 18S rRNA gene and used the QIAamp Stool Mini Kit, but differed in stool storage methods. Studies reporting relatively higher sensitivities largely used the same gene target and extraction kit. Therefore, the observed variability is likely attributable to differences in sample size, infection intensity, PCR cycling conditions, and threshold cutoff levels. Except for two studies [45,56], all the other studies show a relatively good specificity, ranging from 57% (95% CI: 41%–73%) to 94% (95% CI: 89%–97%). The pooled specificity was high, 76% (95%CI: 64%–85%). A substantial variation in diagnostic accuracy across studies suggests possible differences in study populations, methodologies, or testing conditions (Fig 4).

#### Comparison of NAATs and BT with composite reference for the detection of strongyloidiasis.

NAATs demonstrate relatively stable sensitivity across most studies, ranging from 65% (95% CI: 54%–76%) to 100% (95% CI: 96%–100%), with the exception of one study [38], which reported a lower sensitivity of 31% (95% CI: 19%–45%). The pooled sensitivity of NAATs was 93% (95% CI: 77%–98%) (Fig G in S2 Appendix, and Table I in S1 Appendix). The study that reported the lowest sensitivity employed qPCR targeting the 18S rRNA gene and used the QIAamp Stool Mini Kit, with stool samples stored at −20 °C [38]. Similarly, the studies that demonstrated relatively higher sensitivities also targeted the same gene and used the same extraction kit. Therefore, the observed low sensitivity may be attributed to differences in population characteristics, low infection intensity, cyclical conditions, or variations in positivity threshold values.

The sensitivity of BT demonstrates substantial variation across studies, ranging from 29% (95% CI: 24%–35%) to 84% (95% CI: 71%–92%), while specificity remains consistently high at 100% with minimal fluctuation. The pooled sensitivity for BT is relatively low at 59% (95% CI: 48%–69%), showing its limited ability to detect TPs (Fig G in S2 Appendix and Table J in S1 Appendix). All studies used the standard BT method, though variations were observed in sample storage conditions and the time between sample collection and processing. The majority of studies performed a single replicate. Interestingly, some studies that conducted single replicates [35,45] reported higher sensitivity than those performing triplicates [41,55], indicating no clear association between the number of replicates and test sensitivity. Overall, NAATs demonstrated higher sensitivity than BT, while both methods maintained similarly high specificity (Tables I and J in S1 Appendix).

### Detection of enterobiasis

There was one study that compared the diagnostic test accuracy of the Graham Scotch tape and the NAAT in detecting *E. vermicularis* infection [70]. However, because the study reported only pooled positivity across all microscopic methods, the number of individuals identified specifically by the Scotch tape test could not be extracted; therefore, this method was excluded from the analysis.

### Detection of STHs

Out of 35 studies evaluating different HINIs, 11 assessed both NAATs and KK accuracy for the detection of the STHs: *Ascaris*, *Trichuris*, and hookworm (Table 1). One study [51] was excluded from the meta-analysis, as its authors suspected that KK-positive cases with PCR-negative results might have been caused by the misidentification of pollen or other artefacts as *Ascaris* eggs. Four studies did not provide sufficient data on cumulative positive STH data and were therefore excluded from the analysis. Six studies were ultimately included. In all studies, qPCR was used as the primary diagnostic method. Of these, three studies (50%) assessed the accuracy of qPCR in comparison with duplicate KK tests, while the remaining studies (50%) compared qPCR with triplicate KK tests.

#### Comparison of NAATs with KK for the detection of STHs.

The sensitivity of NAATs varied across studies, ranging from 69% (95% CI: 51%–83%) to 94% (95% CI: 93%–96%), except for the two studies discussed above (Azzopardi et al. and Adisakawattana et al.) [44,67]. Both studies reported that NAATs have relatively low sensitivities for *T. trichiura*, while Adisakawattana et al. also demonstrated low sensitivity of NAATs for hookworm detection. The specificity of NAATs varied widely, ranging from 24% (95% CI: 18%–31%) to 98% (95% CI: 96%–99%). In the two studies that reported the lowest specificities, NAATs detected a higher number of positives compared to KK, which likely increased the number of FPs and consequently reduced the specificity (Fig H in S2 Appendix).

#### Comparison of NAATs and KK with composite reference for the detection of STHs.

The sensitivity of the KK test when compared with composite varied widely ranging from 29% (95% CI: 21%–38%) to 94% (95% CI: 90%–97%) with a pooled sensitivity of 64% (95% CI: 40%–83%) The sensitivity of NAATs varied less ranging from 40% (95% CI: 33%–46%) to 96% (95% CI: 94%–97%)with a pooled sensitivity of 84% (95% CI: 94%–97%). NAATs have a higher sensitivity for detecting STH infections compared to KK while maintaining a similarly high specificity (Fig I in S2 Appendix). sROC of the NAAT lies closer to the top-left corner of the plot with an AUC of 0.98, indicating superior overall accuracy, compared to the KK with an AUC of 0.96 (Fig I in S2 Appendix). Both methods perform well above the line of no discrimination, confirming their effectiveness, though NAAT shows a more favourable sensitivity and specificity for detecting STH (Tables K and L in S1 Appendix).

### Comparison of NAATs and flotation methods for the detection of STHs

A total of seven, five, and nine studies assessed *Ascaris*, *Trichuris*, and hookworm infections, using both NAATs and flotation methods (Table 1 and Fig C in S2 Appendix), involving 3,907, 2,837, and 4,075 participants, respectively. In all analyses, two studies [59,65] were excluded from the sensitivity assessments, as neither method detected any positive cases for the three helminth species. All the studies used the ITS1 gene as the target for *Ascaris* and *Trichuris*. For *N. americanus*, both the ITS2 and cytochrome b genes were targeted, while for *Ancylostoma* spp., the ITS1 and ITS2 gene regions were used. DNA storage conditions varied widely, such as storage at −20 °C without preservatives, use of potassium dichromate at 4 °C or room temperature, preservation in 70% or absolute ethanol, use of Zn-PVA, or analysis of fresh stool samples (Table 2). Additional sources of variation included differences in cycling conditions, positivity threshold levels used for qPCR, and variability in the reference tests employed, such as the use of different flotation techniques.

#### Comparison of NAATs and flotation methods in detecting ascariasis.

**Fig 5:**
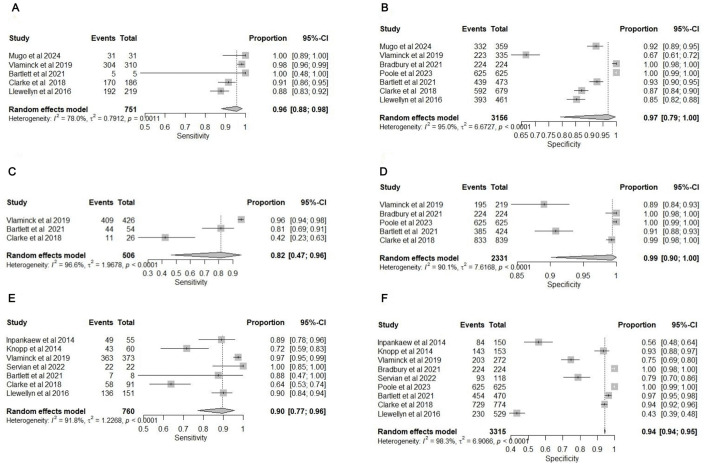
Forest plots for the diagnostic accuracy meta-analysis for *Ascaris*, *Trichuris* and hookworms. **(A)** Forest plot of the sensitivity of nucleic acid amplification tests (NAATs) compared to flotation methods for *Ascaris.* **(B)** Forest plot of the specificity of NAATs compared to flotation methods for *Trichuris.* **(C)** Forest plot of the sensitivity of NAATs compared to flotation methods for hookworms. **(D)** Forest plot of the specificity of NAATs compared to flotation methods for *Ascaris.* **(E)** Forest plot of the sensitivity of NAATs compared to flotation methods for *Trichuris.* **(F)** Forest plot of the specificity of NAATs compared to flotation methods for hookworms. The plots are generated using the Reitsma bivariate model in the mada package in R. Each study’s point estimate and 95% confidence interval are represented by squares and horizontal lines, respectively.

Of the seven included studies (Fig C in S2 Appendix), four compared qPCR with sodium nitrate flotation (SNF), while three compared it with Mini-FLOTAC [48,65,69] (Table 1). NAATs demonstrated high sensitivity for detecting *Ascaris* infection, with a pooled sensitivity of 96% (95% CI: 88%–98%) compared with flotation methods (Fig 5). When compared with the CRS, the pooled sensitivity increased to 98% (95% CI: 92%–99%). In contrast, the sensitivity of flotation methods compared with the CRS was relatively low at 57% (95% CI: 34%–77%), suggesting reduced accuracy in detecting true-positive infections. The specificity of NAATs compared to flotation methods was also high, with a pooled specificity of 97% (95% CI: 79%–100%) (Table 3). Since any sample positive by either method was considered positive in the CRS, both tests showed 100% specificity when compared to the CRS (Fig 5). Overall, these findings indicate that NAATs provide superior accuracy in detecting *Ascaris* infections compared to the flotation method and CRS (Figs J and M in S2 Appendix).

#### Comparison of NAATs and flotation methods in detecting trichuriasis.

For *Trichuris*, two studies [48,65] compared qPCR with Mini-FLOTAC, while three studies compared qPCR with SNF (Table 1). NAATs demonstrated high sensitivity for detecting *Trichuris* infection, with a pooled sensitivity of 82% (95% CI: 47%–96%) when compared with flotation methods. When compared with the CRS, the pooled sensitivity increased to 87% (95% CI: 60%–97%) (Fig 5). Flotation methods also showed similarly high sensitivity of 83% (95% CI: 59%–94%) in detecting *Trichuris* infection compared with the CRS, suggesting its high accuracy in detecting TP infections (Table 3). The specificity of NAATs compared to flotation methods was also high, with a pooled specificity of 99% (95% CI: 90%–100%). Overall, these findings indicate that both NAATs and flotation methods provide high accuracy in detecting *Trichuris* infections (Figs K and M in S2 Appendix).

#### Comparison of NAATs and flotation methods in detecting hookworm infections.

Nine studies compared the diagnostic accuracy of NAATs and flotation methods for detecting hookworm infections (Fig C in S2 Appendix). Of these, four studies compared qPCR with SNF, four with Mini-FLOTAC, and one compared cPCR with SNF. NAATs demonstrated high sensitivity for detecting hookworm infection, with a pooled sensitivity of 90% (95% CI: 77%–96%) compared with flotation methods (Fig 5). When compared with the CRS, the pooled sensitivity increased to 95% (95% CI: 86%–98%). In contrast, the sensitivity of flotation methods compared with the CRS was relatively low at 59% (95% CI: 41%–75%), suggesting reduced accuracy in detecting true-positive infections. The specificity of NAATs compared to flotation methods was also high, with a pooled specificity of 94% (95% CI: 94%–95%). Overall, these findings indicate that NAATs provide superior accuracy in detecting hookworm infections compared to flotation methods (Figs L and M in S2 Appendix).

#### Comparison of NAATs and flotation methods in detecting STHs.

Five studies compared the diagnostic accuracy of NAATs and flotation methods for detecting all three STH infections (Table 1). However, two studies [59,65] reported no positives with either method, and three did not provide sufficient data to calculate cumulative STH outcomes. Consequently, the cumulative diagnostic accuracy of NAATs and flotation methods for STH could not be assessed.

## Discussion

Accurate detection of HINIs is fundamental to individual patient management and population-level control and elimination strategies, particularly in the context of WHO 2030 targets for STH elimination and post–MDA surveillance [18]. This systematic review provides the first comprehensive synthesis to date of diagnostic test accuracy evidence for NAATs for HINIs across diverse clinical and epidemiological settings. The overarching finding is that NAATs consistently demonstrate higher sensitivity than conventional copro-microscopic methods, including KK, flotation techniques, and the BT, especially in low-intensity and post-MDA settings where microscopy performance declines. These findings highlight fundamental limitations of microscopy-based diagnostics for contemporary surveillance needs and support a more prominent, strategic role for NAATs in control and elimination-oriented programmes.

When interpreted within a CRS, pooled sensitivity estimates reflect the relative ability of NAATs and microscopy to detect infections rather than their absolute diagnostic accuracy. Within this framework, NAATs demonstrated high sensitivity for STHs, with pooled estimates of 96% for *A. lumbricoides*, 85% for *T. trichiura*, 88% for hookworm, and 93% for *S. stercoralis* (Table 3). In contrast, KK showed substantially lower and more variable sensitivities across species, with pooled estimates generally ranging from approximately 53% to 70%. Additional KK slides provided inconsistent improvements, and several studies reported minimal or no gain, even with [44,54,62,67] triplicate and quadruplicate KK. Flotation methods also exhibited lower sensitivity than NAATs for *A. lumbricoides* and hookworm, although comparatively better performance was observed for *T. trichiura*. For strongyloidiasis, NAATs markedly outperformed BT, highlighting the persistent diagnostic challenges in using conventional methods.

The sensitivity gap between NAATs and microscopy has important epidemiological and programmatic implications. The observed pooled sensitivity of approximately 43% for KK in detecting hookworm infection implies that more than half of true infections would be missed, whereas for ascariasis and trichuriasis, nearly one-third of infections may be missed in settings relying solely on KK, particularly in post–MDA or low-prevalence contexts. This can lead to substantial underestimation of prevalence, premature assumptions of programmatic success, and inappropriate modification or cessation of control interventions. For strongyloidiasis, the limited sensitivity of the BT is particularly concerning, given the potential for chronic infection, ongoing transmission, and severe disease in immunocompromised individuals. Collectively, these findings indicate that reliance on conventional microscopy alone is increasingly insufficient for accurate surveillance and decision making in control and elimination settings, and that NAATs offer a more sensitive alternative for detecting residual and low-intensity infections.

The superior performance of NAATs reflects fundamental differences in diagnostic principles. Microscopy depends on the visual identification of intact eggs or larvae and is therefore strongly influenced by stool volume, infection intensity, and the structural integrity of parasitic stages. In contrast, NAATs detect parasite DNA, including parasite-derived cell-free DNA (cfDNA), enabling reliable detection even when egg output is low, irregular, degraded, or absent [26]. These limitations of microscopy are particularly evident in *S. stercoralis* and hookworm infections. In strongyloidiasis, microscopic diagnosis relies on the recovery of viable larvae from fresh faecal specimens, and commonly used methods such as Baermann concentration and agar plate culture are highly susceptible to processing delays, suboptimal storage conditions, and loss of larval viability [71]. For hookworm infections, the inherent fragility and rapid degradation of eggs further reduce the sensitivity of microscopy-based methods, thereby amplifying the relative diagnostic advantage of DNA-based approaches [12].

Despite overall superior sensitivity, NAAT performance varied substantially across studies, reflecting considerable methodological heterogeneity. Differences in DNA extraction protocols, mechanical disruption steps, gene targets, PCR platforms, cycling conditions, and positivity thresholds all contributed to variability in sensitivity estimates. Incorporation of bead-beating or other mechanical homogenisation steps was consistently associated with improved DNA yield, particularly for *A. lumbricoides*, although similar benefits were less evident for *T. trichiura* and hookworm [57,72]. Gene target selection also influenced performance; multicopy targets such as ribosomal RNA or internal transcribed spacer regions generally offer improved sensitivity compared with single-copy genes, though trade-offs between sensitivity and specificity may arise depending on assay design [73–75]. Smaller studies reported lower sensitivity, consistent with the influence of sample size, prevalence, and infection intensity on the precision of diagnostic accuracy estimates [23,76,77]. Geographical and population-level factors further contributed to heterogeneity. Included studies spanned diverse endemic regions across South Asia, Southeast Asia, Africa, South America, North America and Oceania, including varied transmission intensities, co-endemic infections, and demographic profiles. Most studies were conducted in community-based settings involving predominantly asymptomatic individuals with low or unknown infection intensities, representing the most diagnostically challenging scenario [36,61,66]. While NAATs performed well under these conditions, their diagnostic performance in symptomatic clinical populations, where parasite burdens and pre-test probabilities are typically higher, cannot be directly inferred from the available evidence and requires further evaluation.

Evidence for NAATs in enterobiasis, compared to scotch tape, remains limited. Only one study evaluated PCR for *E. vermicularis*, reporting high sensitivity and specificity compared with microscopy [70]. Given the inherent limitations of the Scotch tape technique, including the need for repeated sampling due to intermittent egg deposition, NAATs may represent a promising alternative for enterobiasis diagnosis [78–80]. However, the paucity of comparative studies hinders firm conclusions and highlights the need for further research.

Several limitations of this review should be considered when interpreting the findings. The use of a CRS was necessary in the absence of a universally accepted gold standard for several HINIs, but this approach prioritises relative detection performance and limits independent estimation of specificity. By defining infection as detection by either the index or comparator test, CRS-based analyses may inherently inflate specificity estimates and may overestimate pooled sensitivity, particularly when index and reference tests are conditionally dependent. CRS performance is also influenced by the accuracy of component tests and the conditional dependence between the index test and the CRS, all of which may bias summary accuracy measures. Accordingly, pooled estimates derived from CRS comparisons should be interpreted cautiously as average effects across heterogeneous diagnostic contexts, rather than precise measures of absolute accuracy [81,82]. Substantial methodological and clinical heterogeneity was observed across studies, with I² values frequently exceeding 90%. In line with Cochrane guidance for diagnostic test accuracy reviews, specific subgroup or meta-regression analyses were not undertaken due to insufficient numbers of studies within relevant categories and the risk of unstable estimates [83–85]. Instead, heterogeneity was explored through systematic qualitative assessment of key study characteristics, including stool preservation and storage conditions, DNA extraction methods (including the use of mechanical homogenisation), gene targets, PCR thresholds, reference standards, and endemicity settings. The wide variability and limited overlap across these factors precluded meaningful stratification, and pooled estimates should therefore be interpreted as broad summaries across diverse protocols rather than benchmarks for any single diagnostic approach. Furthermore, nearly all studies were conducted in community-based settings, with only two studies [52,59] performed in clinical contexts, limiting the assessment of test accuracy across different healthcare environments. The evidence base was dominated by studies evaluating quantitative PCR–based assays, meaning that the findings largely reflect the diagnostic performance of qPCR rather than NAATs as a broader category. Evidence for alternative amplification platforms, such as LAMP, remains limited, restricting the generalisability of these technologies. Diagnostic performance in symptomatic patients, where infection intensity and pre-test probability differ substantially, remains underexplored. Finally, most studies focused predominantly on diagnostic accuracy outcomes and did not report downstream or operationally relevant measures, such as the impact on treatment decisions, cost-effectiveness, feasibility of laboratories, or turnaround time. This constrains the assessment of the real-world implications of adopting NAATs within routine control and elimination programmes. From a programmatic perspective, diagnostic choice should be guided by epidemiological context, resource availability, and surveillance objectives. In high-prevalence, resource-limited settings requiring rapid results, the KK and flotation methods remain pragmatic options, as their sensitivity is higher in moderate-to-high intensity infections and their implementation costs are low. However, as prevalence declines following repeated rounds of MDA, the limitations of microscopy become increasingly consequential. Cost considerations are particularly important in elimination settings, where low sensitivity markedly increases the cost per true case detected. Although KK has low direct material costs (approximately US$1.7–2.1 per smear), its declining sensitivity in low-prevalence contexts necessitates testing large numbers of individuals to identify a single infection, driving the cost per positive case (exceeding US$100 in post-treatment surveys) [86,87]. NAATs, while more expensive per test (typically ranging from approximately US$10 to US$35), offer substantially improved diagnostic yield and benefit from economies of scale through batch processing and automation [88,89]. When labour costs, repeated sampling, and the programmatic consequences of misclassification and underestimation of prevalence are considered, the higher upfront costs of NAATs may be offset [86,90–92]. However, direct cost-per-case-detected comparisons for STH surveillance remain limited, highlighting the need for dedicated economic evaluations.

Taken together, these findings support a strategic, species-specific integration of NAATs into WHO deworming surveillance rather than their universal replacement of microscopy. For hookworm infection, where KK sensitivity was particularly low, NAATs should be prioritised in sentinel surveillance sites, especially in post-MDA and low-prevalence settings where accurate prevalence estimation is critical. For *S. stercoralis*, given the marked superiority of NAATs over BT, molecular diagnostics should be adopted as the preferred method in both surveillance and clinical contexts, particularly among high-risk populations. For ascariasis and trichuriasis, where the sensitivity gap between NAATs and KK was less pronounced, NAATs may be most appropriately deployed in periodic validation or confirmatory surveys to verify low prevalence estimates and assess transmission interruption, while microscopy may remain acceptable for routine monitoring. A tiered diagnostic strategy, retaining microscopy as a cost-efficient frontline tool while selectively integrating NAATs for high-impact species, sentinel surveillance, and elimination verification, offers a balanced approach that maximises diagnostic accuracy, maintaining feasibility. Emerging innovations, including isothermal amplification methods, sample pooling, and high-throughput workflows, may further enhance the accessibility and cost-effectiveness of molecular diagnostics in endemic settings [93].

## Conclusion

Selecting the appropriate diagnostic tools is critical for the effective control and elimination of HINIs. This systematic review highlights that NAATs consistently outperform conventional copro-microscopy methods, particularly in detecting low-intensity or intermittent infections. By identifying parasite DNA even when eggs are absent, degraded, or intermittently shed, NAATs provide more reliable detection across transmission settings and parasite burdens. While copro-microscopy remains cost-effective and operationally suitable in high-transmission settings, its limited sensitivity in low-prevalence settings risks underestimating infection burden and compromising elimination decision-making. A combined diagnostic approach, maintaining routine microscopy while strategically integrating NAATs for sentinel surveillance, validation surveys, and priority species such as hookworm and *S. stercoralis*, can optimise both accuracy and feasibility. Such integrated strategies will strengthen surveillance systems, improve programmatic decision-making, and accelerate progress toward the WHO 2030 goals for the control and elimination of HINIs.

## Supporting information

S1 File PRISMA 2020 flow diagram for new systematic reviews, which included searches of databases and registers. Source: Page MJ, et al. BMJ 2021;372:n71. https://doi.org/10.1136/bmj.n71. This work is licensed under CC BY 4.0. To view a copy of this license, visit https://creativecommons.org/licenses/by/4.0/.
(PDF)

S1 Table Risk of bias and applicability concerns for included studies based on the Quality Assessment of Diagnostic Accuracy Studies 2 (QUADAS 2) tool. (DOCX)

S1 Checklist PRISMA 2020 checklist. Preferred reporting items for systematic review and meta-analysis 2020 checklist. Source: Page MJ, et al. BMJ 2021;372:n71. https://doi.org/10.1136/bmj.n71. This work is licensed under CC BY 4.0. To view a copy of this license, visit https://creativecommons.org/licenses/by/4.0/.
(DOCX)

S1 Appendix Include supplementary methods and supplementary result tables. (DOCX)

S2 Appendix Include all the supplementary figures. (DOCX)

S1 Data Complete dataset. (XLSX)
