# Supplementary material for: Diagnostic accuracy of nucleic acid amplification tests for human intestinal nematode infections: A systematic review and meta-analysis
Source: PLoS Negl Trop Dis. 2026 Feb 11;20(2):e0013974. doi: 10.1371/journal.pntd.0013974 (PMC12916058; doi:10.1371/journal.pntd.0013974)
Supplement: S1 File — Source: Page MJ, et al. BMJ 2021;372:n71. https://doi.org/10.1136/bmj.n71. This work is licensed under CC BY 4.0. To view a copy of this license, visit https://creativecommons.org/licenses/by/4.0/. (PDF) [file pntd.0013974.s001.pdf]

S1 File. PRISMA 2020 flow diagram for new systematic reviews which included searches of databases and registers only (1)**.**

Source: Page MJ, et al. BMJ 2021;372:n71. doi: 10.1136/bmj.n71. This work is licensed under CC BY 4.0. To view a copy of this license, visit https://creativecommons.org/licenses/by/4.0/

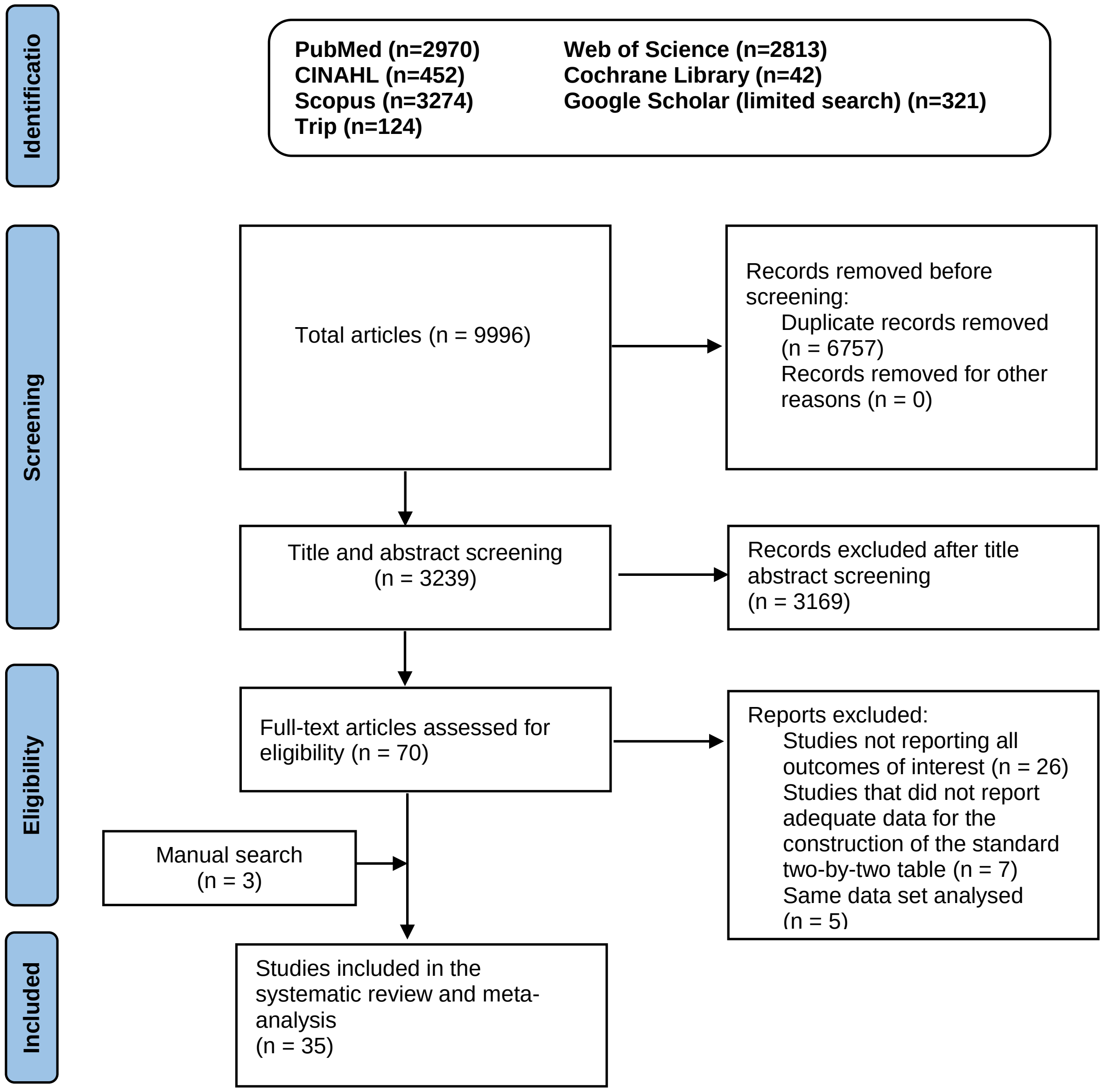

Reference

1. Page MJ, McKenzie JE, Bossuyt PM, Boutron I, Hoffmann TC, Mulrow CD, et al. The PRISMA 2020 statement: an updated guideline for reporting systematic reviews. BMJ. 2021 Mar;372:n71. doi: 10.1136/bmj.n71.
