# Supplementary material for: Diagnostic accuracy of nucleic acid amplification tests for human intestinal nematode infections: A systematic review and meta-analysis
Source: PLoS Negl Trop Dis. 2026 Feb 11;20(2):e0013974. doi: 10.1371/journal.pntd.0013974 (PMC12916058; doi:10.1371/journal.pntd.0013974)
Supplement: S1 Table — (DOCX) [file pntd.0013974.s002.docx]

**S1 Table:** Risk of bias assessment of individual studies using the QUADAS-2 tool

| Study identification details | | Quality assessment (QUADAS - 2) | | | | | | | | | | | | | | | | | |
| --- | --- | --- | --- | --- | --- | --- | --- | --- | --- | --- | --- | --- | --- | --- | --- | --- | --- | --- | --- |
|  |  | Risk of bias | | | | | | | | | | | | | | | Applicability concerns | | |
|  |  | Patient selection | | | | Index test | | | Reference standard | | | Flow and timing | | | | | Patient selection | Index test | Reference standard |
| Author | Year of publication | Was a consecutive or random sample of patients enrolled? Yes/No/Unclear | Was a case-control design avoided? Yes/No/Unclear | Did the study avoid inappropriate exclusions? RISK: Yes/No/Unclear | Risk of bias in patient selection. Low/High/Unclear | Were the index test results interpreted without knowledge of the results of the reference standard? Yes/No/Unclear | Thresholds used were pre-specified. Yes/No/Unclear | Could the conduct or interpretation of the index test have introduced bias? RISK: Low/High/Unclear | Is the reference standard likely to correctly classify the target condition? Yes/No/Unclear | Were the reference standard results interpreted without knowledge of the results of the index test? Yes/No/Unclear | Could the reference standard, its conduct, or its interpretation have introduced bias? RISK: Low/High/Unclear | Was there an appropriate interval between index test(s) and reference standard? Yes/No/Unclear | Did all patients receive a reference standard? Yes/No/Unclear | Did patients receive the same reference standard? Yes/No/Unclear | Were all patients included in the analysis? Yes/No/Unclear | Could the flow and timing, or its interpretation, have introduced bias? RISK: Low/High/Unclear | Is there concern that the included patients do not match the review question? CONCERN: Low/High/Unclear | Is there concern that the index test, its conduct, or interpretation differs from the review question? CONCERN: Low/High/Unclear | Is there concern that the target condition, as defined by the reference standard, does not match the review question? CONCERN: Low/High/Unclear |
| Chankongsin S (1) | 2020 | Yes | Yes | Yes | Low | Unclear | Yes | Unclear | Yes | Unclear | Unclear | Yes | Yes | Yes | Yes | Low | Low | Low | Low |
| Hailu T (2) | 2022 | Yes | Yes | Yes | Low | Yes | Yes | Low | Yes | Yes | Low | Yes | Yes | Yes | Yes | Low | Low | low | Low |
| Becker SL (3) | 2015 | Yes | Yes | Yes | Low | Unclear | Unclear | Unclear | Yes | Unclear | Unclear | Yes | Yes | Yes | Yes | Low | Low | Low | Low |
| Azzopardi KI (4) | 2021 | Yes | Yes | Yes | low | Yes | Yes | Low | Yes | Yes | Low | Yes | Yes | Yes | Yes | Low | Low | Low | Low |
| Ngari MG (5) | 2020 | Unclear | Yes | Unclear | Unclear | Unclear | Yes | Unclear | Yes | Unclear | Unclear | Yes | Yes | Yes | Yes | Low | Low | Low | Low |
| Knopp S (6) | 2014 | Yes | Yes | Yes | Low | Unclear | Yes | Unclear | Yes | Unclear | Unclear | Yes | Yes | Yes | Yes | Low | Low | Low | Low |
| Dunn JC (7) | 2020 | Yes | Yes | Unclear | Unclear | Yes | Yes | Low | Yes | Yes | Low | Yes | Yes | Yes | Yes | Low | Low | Low | Low |
| Chung JB (8) | 2020 | Yes | Yes | Yes | Low | Yes | Yes | Low | Yes | Yes | Low | Yes | Yes | Yes | Yes | Low | Low | Low | Low |
| Meurs L (9) | 2017 | Yes | Yes | Yes | Low | Yes | Yes | Low | Yes | Yes | Low | Yes | Yes | Yes | Yes | Low | Low | Low | Low |
| Mationg MLS (10) | 2017 | Yes | Yes | Unclear | Unclear | Unclear | Yes | Unclear | Yes | Unclear | Unclear | Yes | Yes | Yes | Yes | Low | Low | Low | Low |
| Barda B (11) | 2018 | Yes | Yes | Unclear | Unclear | Unclear | Yes | Unclear | Yes | Unclear | Unclear | Yes | Yes | Yes | Yes | Low | Low | Low | Low |
| Schär F (12) | 2013 | Yes | Yes | Unclear | Unclear | Unclear | Unclear | Unclear | Yes | Unclear | Unclear | Yes | Yes | Yes | Yes | Low | Low | Low | Low |
| van Mens SP (13) | 2013 | Unclear | Yes | Unclear | Unclear | Unclear | Unclear | Unclear | Yes | Unclear | Unclear | Yes | Yes | Yes | Yes | Low | Low | Low | Low |
| Inpankaew T (14) | 2014 | Yes | Yes | Yes | Low | Unclear | Yes | Unclear | Yes | Unclear | Unclear | Yes | Yes | Yes | Yes | Low | Low | Low | Low |
| Easton AV (15) | 2016 | Yes | Yes | Yes | Low | Unclear | Yes | Unclear | Yes | Unclear | Unclear | Yes | Yes | Yes | Yes | Low | Low | Low | Low |
| Pujol BG (16) | 2021 | Yes | Yes | Yes | Low | Yes | Yes | Low | Yes | Yes | Low | Yes | Yes | Yes | Yes | Low | Low | Low | Low |
| [Adisakwattana](https://pubmed.ncbi.nlm.nih.gov/?term=Rotejanaprasert%20C%5BAuthor%5D) P (17) | 2020 | Yes | Yes | Yes | Low | Yes | Yes | Low | Yes | Yes | Low | Yes | Yes | Yes | Yes | Low | Low | Low | Low |
| Soultani M (18) | 2024 | Yes | Yes | Yes | Low | Unclear | Yes | Unclear | Yes | Unclear | Unclear | Yes | Yes | Yes | Yes | Low | Low | Low | Low |
| Keller L (19) | 2020 | Yes | Yes | Yes | Low | Unclear | Yes | Unclear | Yes | Unclear | Unclear | Yes | Yes | Yes | Yes | Low | Low | Low | Low |
| Shilpa G (20) | 2024 | Yes | Yes | Yes | Low | Unclear | Unclear | Unclear | Yes | Unclear | Unclear | Yes | Yes | Yes | Yes | Low | Low | Low | Low |
| Malaga JL (21) | 2024 | Yes | Yes | Yes | Low | Unclear | Yes | Unclear | Yes | Unclear | Unclear | Yes | Yes | Yes | Yes | Low | Low | Low | Low |
| Kristanti H (22) | 2018 | Unclear | Yes | Unclear | Unclear | Yes | Yes | Low | Yes | Yes | Low | Yes | Yes | Yes | Yes | Low | Low | Low | Low |
| Amor A (23) | 2016 | Yes | Yes | Yes | Low | Unclear | Yes | Unclear | Yes | Unclear | Unclear | Yes | Yes | Yes | Yes | Low | Low | Low | Low |
| Aung E (24) | 2022 | Yes | Yes | Yes | Low | Unclear | Yes | Unclear | Yes | Unclear | Unclear | Yes | Yes | Yes | Yes | Low | Low | Low | Low |
| Fleitas PE (25) | 2021 | Yes | Yes | Yes | Low | Yes | Yes | Low | Yes | Yes | Low | Yes | Yes | Yes | Yes | Low | Low | Low | Low |
| Amor A (26) | 2020 | Yes | Yes | Yes | Low | Yes | Yes | Low | Yes | Yes | Low | Yes | Yes | Yes | Yes | Low | Low | Low | Low |
| Mugo RM (27) | 2024 | Yes | Yes | Yes | Low | Unclear | Yes | Unclear | Yes | Unclear | Unclear | Yes | Yes | Yes | Yes | Low | Low | Low | Low |
| Vlaminck J (28) | 2019 | Yes | Yes | Yes | Low | Yes | Yes | Low | Yes | Yes | Low | Yes | Yes | Yes | Yes | Low | Low | Low | Low |
| Noor Z (29) | 2023 | Yes | Yes | Yes | Low | Unclear | Yes | Unclear | Yes | Unclear | Unclear | Yes | Yes | Yes | Yes | Low | Low | Low | Low |
| Bradbury RS (30) | 2021 | No | Yes | Yes | High | Unclear | Yes | Unclear | Yes | Unclear | Unclear | Yes | Yes | Yes | Yes | Low | Low | Low | Low |
| Servián A (31) | 2022 | Yes | Yes | Yes | Low | Unclear | Yes | Unclear | Yes | Unclear | Unclear | Yes | Yes | Yes | Yes | Low | Low | Low | Low |
| Poole C (32) | 2023 | Yes | Yes | Yes | Low | Unclear | Yes | Unclear | Yes | Unclear | Unclear | Yes | Yes | Yes | Yes | Low | Low | Low | Low |
| Bartlett AW (33) | 2021 | Yes | Yes | Yes | Low | Yes | Yes | Low | Yes | Yes | Low | Yes | Yes | Yes | Yes | Low | Low | Low | Low |
| Clarke NE (34) | 2018 | Yes | Yes | Yes | Low | Yes | Yes | Low | Yes | Yes | Low | Yes | Yes | Yes | Yes | Low | Low | Low | Low |
| Llewellyn S (35) | 2016 | Yes | Yes | Yes | Low | Yes | Yes | Low | Yes | Yes | Low | Yes | Yes | Yes | Yes | Low | Low | Low | Low |

**References**

1. Chankongsin S, Wampfler R, Ruf MT, Odermatt P, Marti H, Nickel B, Keoluangkhot V, Neumayr A. *Strongyloides stercoralis* prevalence and diagnostics in Vientiane, Lao People's Democratic Republic. Infect Dis Poverty. 2020 Sep 21;9(1):133. doi: 10.1186/s40249-020-00750-y.

2. Hailu, T., Amor, A., Nibret, E. et al. Evaluation of five diagnostic methods for *Strongyloides stercoralis* infection in Amhara National Regional State, northwest Ethiopia. BMC Infect Dis 22, 297 (2022). doi: 10.1186/s12879-022-07299-1

3. Becker SL, Piraisoody N, Kramme S, Marti H, Silué KD, Panning M, Nickel B, Kern WV, Herrmann M, Hatz CF, N'Goran EK, Utzinger J, von Müller L. Real-time PCR for detection of *Strongyloides stercoralis* in human stool samples from Côte d'Ivoire: diagnostic accuracy, inter-laboratory comparison and patterns of hookworm co-infection. Acta Trop. 2015 Oct;150:210-7. doi: 10.1016/j.actatropica.2015.07.019.

4. Azzopardi KI, Hardy M, Baker C, Bonnici R, Llewellyn S, McCarthy JS, Traub RJ, Steer AC. Detection of six soil-transmitted helminths in human stool by qPCR- a systematic workflow. PLoS One. 2021 Sep 30;16(9):e0258039. doi: 10.1371/journal.pone.0258039.

5. Ngari MG, Mwangi IN, Njoroge MP, Kinyua J, Osuna FA, Kimeu BM, et al. Development and evaluation of a loop-mediated isothermal amplification (LAMP) diagnostic test for detection of whipworm, Trichuris trichiura, in faecal samples. J Helminthol. 2020 Apr 2;94:e142. doi: 10.1017/S0022149X2000022X.

6. Knopp S, Salim N, Schindler T, Voules DAK, Rothen J, Lweno O, et al. Diagnostic accuracy of Kato-Katz, FLOTAC, Baermann, and PCR methods for the detection of light-intensity hookworm and Strongyloides stercoralis infections in Tanzania. Am J Trop Med Hyg. 2014 Mar;90(3):535-545. doi: 10.4269/ajtmh.13-0268.

7. Dunn JC, Papaiakovou M, Han KT, Chooneea D, Bettis AA, Wyine NY, et al. The increased sensitivity of qPCR in comparison to Kato-Katz is required for the accurate assessment of the prevalence of soil-transmitted helminth infection in settings that have received multiple rounds of mass drug administration. Parasites Vectors 13, 324 (2020). doi: org/10.1186/s13071-020-04197-w

8. Chung JB, Pilotte N, Ercumen A, Grant JR, Maasch JRMA, Gonzalez AM, Ester AC, Arnold BF, Rahman M, Haque R, Hubbard AE, Luby SP, Williams SA, Colford JM Jr. Comparison of multi-parallel qPCR and double-slide Kato-Katz for detection of soil-transmitted helminth infection among children in rural Bangladesh. PLoS Negl Trop Dis. 2020 Apr 24;14(4):e0008087. doi: 10.1371/journal.pntd.0008087.

9. Meurs L, Polderman AM, Vinkeles Melchers NV, Brienen EA, Verweij JJ, Groosjohan B, Mendes F, Mechendura M, Hepp DH, Langenberg MC, Edelenbosch R, Polman K, van Lieshout L. Diagnosing Polyparasitism in a High-Prevalence Setting in Beira, Mozambique: Detection of intestinal parasites in fecal samples by microscopy and real-time PCR. PLoS Negl Trop Dis. 2017 Jan 23;11(1):e0005310. doi: 10.1371/journal.pntd.0005310.

10. Mationg MLS, Gordon CA, Tallo VL, Olveda RM, Alday PP, Reñosa MDC, et al. Status of soil-transmitted helminth infections in schoolchildren in Laguna Province, the Philippines: Determined by parasitological and molecular diagnostic techniques. PLoS Negl Trop Dis. 2017 Nov 6;11(11):e0006022. doi: 10.1371/journal.pntd.0006022.

11. Barda B, Wampfler R, Sayasone S, Phongluxa K, Xayavong S, Keoduangsy K, Schindler C, Keiser J. Evaluation of Two DNA Extraction Methods for Detection of *Strongyloides stercoralis* Infection. J Clin Microbiol. 2018 Mar 26;56(4):e01941-17. doi: 10.1128/JCM.01941-17.

12. Schär F, Odermatt P, Khieu V, Panning M, Duong S, Muth S, Marti H, Kramme S. Evaluation of real-time PCR for *Strongyloides stercoralis* and hookworm as diagnostic tool in asymptomatic schoolchildren in Cambodia. Acta Trop. 2013 May;126(2):89-92. doi: 10.1016/j.actatropica.2012.12.012.

13. van Mens SP, Aryeetey Y, Yazdanbakhsh M, van Lieshout L, Boakye D, Verweij JJ. Comparison of real-time PCR and Kato smear microscopy for the detection of hookworm infections in three consecutive faecal samples from schoolchildren in Ghana. Trans R Soc Trop Med Hyg. 2013 Apr;107(4):269-71. doi: 10.1093/trstmh/trs094.

14. Inpankaew T, Schär F, Khieu V, Muth S, Dalsgaard A, Marti H, Traub RJ, Odermatt P. Simple fecal flotation is a superior alternative to quadruple Kato-Katz smear examination for the detection of hookworm eggs in human stool. PLoS Negl Trop Dis. 2014 Dec 18;8(12):e3313. doi: 10.1371/journal.pntd.0003313.

15. Easton A V., Oliveira RG, O’Connell EM, Kepha S, Mwandawiro CS, Njenga SM, et al. Multi-parallel qPCR provides increased sensitivity and diagnostic breadth for gastrointestinal parasites of humans: field-based inferences on the impact of mass deworming. Parasit Vectors. 2016 Jan 27;9:38. doi: 10.1186/s13071-016-1314-y.

16. Grau-Pujol B, Martí-Soler H, Escola V, Demontis M, Jamine JC, Gandasegui J, et al. Towards soil-transmitted helminths transmission interruption: The impact of diagnostic tools on infection prediction in a low intensity setting in Southern Mozambique. PLoS Negl Trop Dis. 2021 Oct 25;15(10):e0009803. doi: 10.1371/journal.pntd.0009803.

17. Adisakwattana P, Yoonuan T, Phuphisut O, Poodeepiyasawat A, Homsuwan N, Gordon CA, et al. Clinical helminthiases in Thailand border regions show elevated prevalence levels using qPCR diagnostics combined with traditional microscopic methods. Parasit Vectors. 2020 Aug 12;13(1):416. doi: 10.1186/s13071-020-04290-0.

18. Soultani M, Bartlett AW, Mendes EP, Hii SF, Traub R, Palmeirim MS, et al. Estimating prevalence and infection intensity of soil-transmitted helminths using quantitative polymerase chain reaction and Kato-Katz in school-age children in Angola. Am J Trop Med Hyg. 2024 Apr 30;110(6):1145-1151. doi: 10.4269/ajtmh.23-0821.

19. Keller L, Patel C, Welsche S, Schindler T, Hürlimann E, Keiser J. Performance of the Kato-Katz method and real time polymerase chain reaction for the diagnosis of soil-transmitted helminthiasis in the framework of a randomised controlled trial: treatment efficacy and day-to-day variation. Parasit Vectors. 2020 Oct 15;13(1):517. doi: 10.1186/s13071-020-04401-x.

20. Gaidhane S, Gaidhane A, Khatib MN, Telrandhe S, Patil M, Saxena D, et al. Estimation of the parasitic burden of soil-transmitted helminths among pregnant women in the Maharashtra state of India using qPCR: A community-based study. Indian J Community Med. 2024 Jan-Feb;49(1):157-164. doi: 10.4103/ijcm.ijcm_249_23.

21. Malaga JL, Fernandez-Baca M V, Castellanos-Gonzalez A, Tanabe MB, Tift C, Morales ML, et al. The Recombinase polymerase amplification test for *Strongyloides stercoralis* is more sensitive than microscopy and real-time PCR in high-risk communities of Cusco, Peru. Pathogens. 2024 Oct 3;13(10):869. doi: 10.3390/pathogens13100869.

22. Kristanti H, Meyanti F, Wijayanti MA, Mahendradhata Y, Polman K, Chappuis F, et al. Diagnostic comparison of Baermann funnel, Koga agar plate culture and polymerase chain reaction for detection of human Strongyloides stercoralis infection in Maluku, Indonesia. Parasitol Res. 2018 Oct;117(10):3229-3235. doi: 10.1007/s00436-018-6021-5.

23. Amor A, Rodriguez E, Saugar JM, Arroyo A, López-Quintana B, Abera B, et al. High prevalence of *Strongyloides stercoralis* in school-aged children in a rural highland of north-western Ethiopia: the role of intensive diagnostic work-up. Parasit Vectors. 2016 Dec 1;9(1):617. doi: 10.1186/s13071-016-1912-8.

24. Aung E, Han KT, Gordon CA, Hlaing NN, Aye MM, Htun MW, et al. High prevalence of soil-transmitted helminth infections in Myanmar schoolchildren. Infect Dis Poverty. 2022 Mar 10;11(1):28. doi: 10.1186/s40249-022-00952-6.

25. Fleitas PE, Vargas PA, Caro N, Almazan MC, Echazú A, Juárez M, et al. Scope and limitations of a multiplex conventional PCR for the diagnosis of *S. stercoralis* and hookworms. Braz J Infect Dis. 2021 Nov-Dec;25(6):101649. doi: 10.1016/j.bjid.2021.101649.

26. Aramendia AA, Anegagrie M, Zewdie D, Dacal E, Saugar JM, Herrador Z, et al. Epidemiology of intestinal helminthiases in a rural community of Ethiopia: Is it time to expand control programs to include *Strongyloides stercoralis* and the entire community? PLoS Negl Trop Dis. 2020 Jun 4;14(6):e0008315. doi: 10.1371/journal.pntd.0008315.

27. Mugo RM, Rausch S, Musimbi ZD, Strube C, Raulf M-K, Landt O, et al. Evaluation of copromicroscopy, multiplex-qPCR and antibody serology for monitoring of human ascariasis in endemic settings. PLoS Negl Trop Dis. 2024 Jun 18;18(6):e0012279. doi: 10.1371/journal.pntd.0012279.

28. Vlaminck J, Cools P, Albonico M, Ame S, Ayana M, et al. (2019) Therapeutic efficacy of albendazole against soil-transmitted helminthiasis in children measured by five diagnostic methods. PLOS Neglected Tropical Diseases 13(8): e0007471. doi: 10.1371/journal.pntd.0007471

29. Noor Z, Hossain B, Khan SS, Kabir M, Bhuiyan ATMRH, Alam MS, et al. Prevalence of soil-transmitted helminths at baseline and after albendazole treatment in the school-age children of forcibly displaced Myanmar nationals in Bangladesh. Am J Trop Med Hyg. 2023 Aug 7;109(3):656-666. doi: 10.4269/ajtmh.23-0260.

30. Bradbury RS, Lane M, Arguello I, Handali S, Cooley G, Pilotte N, et al. Parasitic disease surveillance, Mississippi, USA. Emerg Infect Dis. 2021 Aug;27(8):2201-2204. doi: 10.3201/eid2708.204318.

31. Servián A, Repetto SA, Lorena Zonta M, Navone GT. Human hookworms from Argentina: Differential diagnosis of *Necator americanus* and *Ancylostoma duodenale* in endemic populations from Buenos Aires and Misiones. Rev Argent Microbiol. 2022 Oct-Dec;54(4):268-281. doi: 10.1016/j.ram.2022.05.005.

32. Poole C, Barker T, Bradbury R, Capone D, Chatham AH, Handali S, et al. Cross-sectional study of soil-transmitted helminthiases in black belt region of Alabama, USA. Emerg Infect Dis. 2023 Dec;29(12):2461-2470. doi: 10.3201/eid2912.230751.

33. Bartlett AW, Traub R, Amaral S, Hii SF, Clarke NE, Matthews A, et al. Comparison between quantitative polymerase chain reaction and sodium nitrate flotation microscopy in diagnosing soil-transmitted helminth infections. T Am J Trop Med Hyg. 2021;105(5):1210-1213. doi:10.4269/ajtmh.21-0227

34. Clarke NE, Llewellyn S, Traub RJ, McCarthy J, Richardson A, Nery SV. Quantitative polymerase chain reaction for diagnosis of soil-transmitted helminth infections: A comparison with a flotation-based technique and an investigation of variability in DNA detection. Am J Trop Med Hyg. 2018 Oct;99(4):1033-1040. doi: 10.4269/ajtmh.18-0356.

35. Llewellyn S, Inpankaew T, Nery SV, Gray DJ, Verweij JJ, Clements ACA, et al. Application of a Multiplex Quantitative PCR to Assess Prevalence and Intensity Of Intestinal Parasite Infections in a Controlled Clinical Trial. PLoS Negl Trop Dis. 2016 Jan 28;10(1):e0004380. doi: 10.1371/journal.pntd.0004380.
