# Supplementary material for: Diagnostic accuracy of nucleic acid amplification tests for human intestinal nematode infections: A systematic review and meta-analysis
Source: PLoS Negl Trop Dis. 2026 Feb 11;20(2):e0013974. doi: 10.1371/journal.pntd.0013974 (PMC12916058; doi:10.1371/journal.pntd.0013974)
Supplement: S1 Appendix — (DOCX) [file pntd.0013974.s004.docx]

# Table A. Search strategies used in each database

| Database | Search Strategy | Date |
| --- | --- | --- |
| PubMed | ((("ascaris"[All Fields] OR "roundworm"[All Fields] OR "necator"[All Fields] OR "ancylostoma"[All Fields] OR "hookworm"[All Fields] OR "strongyloides"[All Fields] OR "threadworm"[All Fields] OR "trichuris"[All Fields] OR "whipworm"[All Fields] OR "enterobius"[All Fields] OR "pinworm"[All Fields] OR "soil transmitted helminth"[All Fields] OR "geohelminth"[All Fields] OR "intestinal nematode"[All Fields]) AND ("diagnos*"[All Fields] OR "diagnosis"[All Fields] OR "detect"[All Fields] OR "screen"[All Fields] OR "investigat*"[All Fields] OR "investigation"[All Fields] OR "polymerase chain reaction"[All Fields] OR "PCR"[All Fields] OR "molecular"[All Fields] OR "nucleic acid amplification"[All Fields] OR "NAAT"[All Fields] OR "isothermal amplification"[All Fields] OR "loop mediated isothermal amplification"[All Fields] OR "LAMP"[All Fields] OR "microscopy"[All Fields] OR "microscop*"[All Fields] OR "kato katz"[All Fields] OR "baermann technique"[All Fields] OR "scotch tape"[All Fields] OR "flotation"[All Fields] OR "flotac"[All Fields] OR "miniflotac"[All Fields] OR "mcmasters"[All Fields] OR "flot*"[All Fields]))) Filters: in the last 10 years, Humans | 26^th^ September 2024 |
| CINAHL (EBSCOhost) | (TX ascaris OR TX roundworm OR TX necator OR TX ancylostoma OR TX hookworm OR TX strongyloides OR TX threadworm OR TX trichuris OR TX whipworm OR TX enterobius OR TX pinworm OR TX "soil transmitted helminth" OR TX geohelminth OR TX "intestinal nematode") AND (TX diagnos* OR TX detect OR TX screen OR TX investigat* OR TX "polymerase chain reaction" OR TX PCR OR TX molecular OR TX "nucleic acid amplification" OR TX NAAT OR TX "isothermal amplification" OR TX "loop mediated isothermal amplification" OR TX LAMP OR TX microscopy OR TX microscop* OR TX "kato katz" OR TX "baermann technique" OR TX "scotch tape" OR TX flotation OR TX flotac OR TX miniflotac OR TX mcmasters OR TX flot*) | 26^th^ September 2024 |
| Scopus | TITLE-ABS-KEY(ascaris OR roundworm OR necator OR ancylostoma OR hookworm OR strongyloides OR threadworm OR trichuris OR whipworm OR enterobius OR pinworm OR "soil transmitted helminth" OR geohelminth OR "intestinal nematode") AND TITLE-ABS-KEY(diagnos* OR detect OR screen OR investigat* OR "polymerase chain reaction" OR PCR OR molecular OR "nucleic acid amplification" OR NAAT OR "isothermal amplification" OR "loop mediated isothermal amplification" OR LAMP OR microscopy OR microscop* OR "kato katz" OR "baermann technique" OR "scotch tape" OR flotation OR flotac OR miniflotac OR mcmasters OR flot*) | 26^th^ September 2024 |
| TRIP Database | ascaris OR roundworm OR necator OR ancylostoma OR hookworm OR strongyloides OR threadworm OR trichuris OR whipworm OR enterobius OR pinworm OR "soil transmitted helminth" OR geohelminth OR "intestinal nematode" AND diagnos* OR detect OR screen OR investigat* OR "polymerase chain reaction" OR PCR OR molecular OR "nucleic acid amplification" OR NAAT OR "isothermal amplification" OR "loop mediated isothermal amplification" OR LAMP OR microscopy OR microscop* OR "kato katz" OR "baermann technique" OR "scotch tape" OR flotation OR flotac OR miniflotac OR mcmasters OR flot* | 26^th^ September 2024 |
| Web of Science | TS=(ascaris OR roundworm OR necator OR ancylostoma OR hookworm OR strongyloides OR threadworm OR trichuris OR whipworm OR enterobius OR pinworm OR "soil transmitted helminth" OR geohelminth OR "intestinal nematode") AND TS=(diagnos* OR detect OR screen OR investigat* OR "polymerase chain reaction" OR PCR OR molecular OR "nucleic acid amplification" OR NAAT OR "isothermal amplification" OR "loop mediated isothermal amplification" OR LAMP OR microscopy OR microscop* OR "kato katz" OR "baermann technique" OR "scotch tape" OR flotation OR flotac OR miniflotac OR mcmasters OR flot*) | 26^th^ September 2024 |
| Cochrane Library | ([ascaris OR roundworm OR necator OR ancylostoma OR hookworm OR strongyloides OR threadworm OR trichuris OR whipworm OR enterobius OR pinworm OR "soil transmitted helminth" OR geohelminth OR "intestinal nematode"]) AND ([diagnos* OR detect OR screen OR investigat* OR "polymerase chain reaction" OR PCR OR molecular OR "nucleic acid amplification" OR NAAT OR "isothermal amplification" OR "loop mediated isothermal amplification" OR LAMP OR microscopy OR microscop* OR "kato katz" OR "baermann technique" OR "scotch tape" OR flotation OR flotac OR miniflotac OR mcmasters OR flot*]) | 26^th^ September 2024 |
| Google Scholar | "ascaris" OR "roundworm" OR "necator" OR "ancylostoma" OR "hookworm" OR "strongyloides" OR "threadworm" OR "trichuris" OR "whipworm" OR "enterobius" OR "pinworm" OR "soil transmitted helminth" OR "geohelminth" OR "intestinal nematode" AND ("diagnosis" OR diagnos* OR detect OR screen OR investigat* OR "polymerase chain reaction" OR PCR OR molecular OR "nucleic acid amplification" OR NAAT OR "isothermal amplification" OR "loop mediated isothermal amplification" OR LAMP OR microscopy OR microscop* OR "kato katz" OR "baermann technique" OR "scotch tape" OR flotation OR flotac OR miniflotac OR mcmasters OR flot*) | 26^th^ September 2024 |

**Table B. Details of the full-text screening**

| **Author** | **Year of publication** | **DOI** | **Include/**  **Exclude** | **Reason for exclusion** |
| --- | --- | --- | --- | --- |
| Chankongsin S | 2020 | 10.1186/s40249-020-00750-y. | Include | - |
| Hailu T | 2022 | 10.1186/s12879-022-07299-1 | Include | - |
| Becker SL | 2015 | 10.1016/j.actatropica.2015.07.019 | Include | - |
| Autier B | 2021 | [10.1051/parasite/2021034](https://doi.org/10.1051/parasite/2021034) | Exclude | Inadequate data to construct relevant 2×2 tables |
| Fleitas PE | 2021 | [10.1016/j.bjid.2021.101649](https://doi.org/10.1016/j.bjid.2021.101649) | Include | - |
| Azzopardi KI | 2021 | [10.1371/journal.pone.0258039](https://doi.org/10.1371/journal.pone.0258039) | Include | - |
| Ngari MG | 2020 | [10.1017/S0022149X2000022X](https://doi.org/10.1017/S0022149X2000022X) | Include | - |
| Knopp S | 2014 | 10.4269/ajtmh.13-0268 | Include | - |
| Dunn JC | 2020 | [10.1186/s13071-020-04197-w](https://doi.org/10.1186/s13071-020-04197-w) | Include | - |
| Chung JB | 2020 | [10.1371/journal.pntd.0008087](https://doi.org/10.1371/journal.pntd.0008087) | Include | - |
| Meurs L | 2017 | 10.1371/journal.pntd.0005310 | Include | - |
| Mationg MLS | 2017 | 10.1371/journal.pntd.0006022 | Include | - |
| Barda B | 2018 | 10.1128/JCM.01941-17 | Include | - |
| Schär F | 2013 | 10.1016/j.actatropica.2012.12.012 | Include | - |
| Sitta RB | 2014 | [10.1017/S0031182013002035](https://doi.org/10.1017/S0031182013002035) | Exclude | The Baermann test was not conducted |
| Mens SP | 2013 | [10.1093/trstmh/trs094](https://doi.org/10.1093/trstmh/trs094) | Include | - |
| Inpankaew T | 2014 | [10.1371/journal.pntd.0003313](https://doi.org/10.1371/journal.pntd.0003313) | Include | - |
| Easton AV | 2016 | 10.1186/s13071-016-1314-y | Include | - |
| Arndt MB | 2013 | [10.1371/journal.pone.0081915](https://doi.org/10.1371/journal.pone.0081915) | Exclude | Inadequate data to construct relevant 2×2 tables |
| Mejia R | 2013 | 10.4269/ajtmh.12-0726 | Exclude | Inadequate data to construct relevant 2×2 tables |
| Easton AV | 2017 | 10.1186/s13071-017-2164-y | Exclude | Same data set analysed (Easton et al, 2016) |
| Becker SL | 2015 | 10.1016/j.cmi.2015.02.016 | Exclude | Microscopic method not specified |
| Pujol BG | 2021 | [10.1371/journal.pntd.0009803](https://doi.org/10.1371/journal.pntd.0009803) | Include | - |
| Adisakwattana P | 2020 | [10.1186/s13071-020-04290-0](https://doi.org/10.1186/s13071-020-04290-0) | Include | - |
| Soultani M | 2024 | 10.4269/ajtmh.23-0821 | Include | - |
| Ulaganeethi R | 2023 | [10.1016/j.ijmmb.2023.100427](https://doi.org/10.1016/j.ijmmb.2023.100427Get%20rights%20and%20content) | Exclude | The Kato-Katz test or the flotation methods were not conducted |
| Keller L | 2020 | [10.1186/s13071-020-04401-x](https://doi.org/10.1186/s13071-020-04401-x) | Include | - |
| Barda B | 2020 | 10.1186/s12866-020-01963-9 | Exclude | Inadequate data to construct relevant 2×2 tables |
| Gaidhane S | 2024 | [10.4103/ijcm.ijcm_249_23](https://journals.lww.com/ijcm/fulltext/2024/49010/estimation_of_the_parasitic_burden_of.26.aspx) | Include | - |
| Hii SF | 2018 | 10.4269/ajtmh.18-0276 | Exclude | Microscopic method not specified |
| Bartlett AW | 2021 | [10.4269/ajtmh.21-0227](https://doi.org/10.4269/ajtmh.21-0227) | Include | - |
| Rotejanaprasert C | 2023 | [10.1098/rstb.2022.0281](https://doi.org/10.1098/rstb.2022.0281) | Exclude | Same data set analysed (Adisakwattana et al, 2020) |
| [Clarke NE](https://pubmed.ncbi.nlm.nih.gov/?term=Clarke+NE&cauthor_id=30062984) | 2018 | 10.4269/ajtmh.18-0356 | Include | - |
| Cools P | 2019 | [10.1371/journal.pntd.0007446](https://doi.org/10.1371/journal.pntd.0007446) | Exclude | Inadequate data to construct relevant 2×2 tables |
| Llewellyn S | 2016 | 10.1371/journal.pntd.0004380 | Include | - |
| Verweij JJ | 2014 | 10.1017/S0031182014000419 | Exclude | The Kato-Katz test or the flotation methods were not conducted |
| Ulaganeeth R | 2023 | 10.1016/j.ijmmb.2023.100427 | Exclude | The Kato-Katz test or the flotation methods were not conducted |
| Nils Pilotte | 2016 | 10.1371/journal.pntd.0004578 | Exclude | No prevalence |
| Le B | 2022 | 10.1371/journal.pntd.0010350 | Exclude | The Kato-Katz test or the flotation methods were not conducted |
| [Kaisar MM](https://pubmed.ncbi.nlm.nih.gov/?term=%22KAISAR%20MMM%22%5BAuthor%5D) | 2017 | 10.1017/S0031182017000129 | Exclude | The Kato-Katz test or the flotation methods were not conducted |
| [Gordon CA](https://pubmed.ncbi.nlm.nih.gov/?term=Gordon+CA&cauthor_id=25858090) | 2015 | 10.1016/j.ijpara.2015.02.011 | Exclude | The Kato-Katz test or the flotation methods were not conducted |
| [Hughes A](https://parasitesandvectors.biomedcentral.com/articles/10.1186/s13071-023-05809-x#auth-Angus-Hughes-Aff1) | 2023 | 10.1186/s13071-023-05809-x | Exclude | The Kato-Katz test or the flotation methods were not conducted |
| Autier B | 2021 | 10.1051/parasite/2021034. | Exclude | Microscopic method not specified |
| Zendejas-Heredia PA | 2021 | 10.1371/journal.pntd.0009395 | Exclude | Inadequate data to construct relevant 2×2 tables |
| Mationg MLS | 2021 | 10.1371/journal.pntd.0010008 | Exclude | PCR was not conducted |
| Aung E | 2022 | 10.1186/s40249-022-00952-6 | Include |  |
| Bärenbold O | 2017 | 10.1371/journal.pntd.0005953 | Exclude | PCR was not conducted |
| Hawkins KR | 2016 | 10.1371/journal.pntd.0004985 | Exclude | No prevalence |
| Assefa LM | 2014 | 10.1371/journal.pntd.0002843 | Exclude | PCR was not conducted |
| Pujol BG | 2022 | 10.4269/ajtmh.21-0948 | Exclude | Same data set analysed Part with in another study (Pujol et al, 2021) |
| Manuel M | 2024 | 10.1371/journal.pntd.0012416 | Exclude | For flotation, only soil samples were used, but for PCR, both stool and soil samples were used |
| Levecke B | 2020 | 10.1371/journal.pntd.0008296 | Exclude | Same data set analysed as Vlaminck et al, 2019 |
| Kamdem CN | 2022 | 10.1038/s41598-022-18285-7. | Exclude | PCR was not conducted |
| Noor Z | 2023 | 10.4269/ajtmh.23-0260 | Include | - |
| Poole C | 2023 | 10.3201/eid2912.230751 | Include | - |
| Servián A | 2022 | 10.1016/j.ram.2022.05.005 | Include | - |
| Gandasegui J | 2021 | 10.1186/s13071-021-04941-w | Exclude | Microscopic examination was not conducted |
| Vlaminck J | 2019 | 10.1371/journal.pntd.0007471 | Include | - |
| Mugo RM | 2024 | 10.1371/journal.pntd.0012279 | Include | - |
| Lim-Leroy A | 2020 | 10.1371/journal.pone.0239680 | Exclude | The Kato-Katz test or the flotation methods were not conducted |
| Oyegue-Liabagui SL | 2020 | 10.1186/s12879-020-05071-x | Exclude | Microscopic examination was not conducted |
| Ström G | 2018 | 10.1016/j.scitotenv.2017.11.254 | Exclude | PCR done for zoonotic *Ascaris* and *Trichuris* species only |
| Squire SA | 2018 | 10.1007/s00436-018-6017-1 | Exclude | The Kato-Katz test or the flotation methods were not conducted |
| Amor A | 2020 | 10.1371/journal.pntd.0008315 | Include | - |
| Hailu T | 2022 | 10.1371/journal.pntd.0010299 | Exclude | Same data set analysed as Hailu et al, 2022 |
| Korzeniewski K | 2023 | 10.5603/imh.97560 | Exclude | PCR was not conducted for helminths, but only for protozoa |
| Tchakounté BN | 2018 | 10.4314/ejhs.v28i1.8 | Exclude | PCR was not conducted |
| Bradbury RS | 2021 | 10.3201/eid2708.204318 | Include | - |
| Papaiakovou M | 2019 | 10.1016/j.pt.2019.04.006 | Exclude | No prevalence |
| Wong WK | 2019 | 10.1007/s00436-019-06406-7 | Exclude | Inadequate data to construct relevant 2×2 tables |
| Amor A | 2016 | 10.1186/s13071-016-1912-8 | Include | - |
| Kristanti H | 2018 | 10.1007/s00436-018-6021-5 | Include | - |
| Malaga JL | 2024 | 10.3390/pathogens13100869 | Include | - |

**Table C. Diagnostic accuracy of nucleic acid amplification tests compared with a composite reference standard for detecting ascariasis**

| Study details | Index test (NAAT) | | Reference test (composite of NAAT and KK) | | | Sensitivity %  (95%CI) | Specificity % (95%CI) | Positive predictive value % (95%CI) | Negative predictive value % (95%CI) | Accuracy % (95%CI) |
| --- | --- | --- | --- | --- | --- | --- | --- | --- | --- | --- |
|  |  |  | + n | - n | Type |  |  |  |  |  |
| Azzopardi et al. 2021 (1) | qPCR | + | 7 | 0 | qPCR +  D-KK | 78 (40 – 97) | 100 (89 – 100) | 100 (59 – 100) | 94 (82 – 98) | 95 (83 – 99) |
|  |  | - | 2 | 31 |  |  |  |  |  |  |
| Dunn et al. 2020 (2) | qPCR | + | 47 | 0 | qPCR +  S-KK | 100 (92 – 100 ) | 100 (99 – 100) | 100 (92 – 100) | 100(99 – 100) | 100 (99 – 100) |
|  |  | - | 0 | 601 |  |  |  |  |  |  |
| Meurs et al. 2017 (3) | qPCR | + | 162 | 0 | qPCR +  S-KK | 100 (98 – 100) | 100 (97 – 100) | 100 (98 – 100) | 100 (97 – 100) | 100 (99 – 100) |
|  |  | - | 0 | 141 |  |  |  |  |  |  |
| Mationg et al. 2017 (4) | qPCR | + | 160 | 0 | qPCR +  T-KK | 90 (84 – 94) | 100 (96 – 100) | 68 (62 – 73) | 100 (98 – 100) | 83 (75 – 88) |
|  |  | - | 18 | 85 |  |  |  |  |  |  |
| Easton et al. 2016 (5) | qPCR | + | 138 | 0 | qPCR +  D-KK | 97 (92 – 99) | 100 (99 – 100) | 100 (97 – 100) | 99 (98 – 100) | 99 (98 – 100) |
|  |  | - | 5 | 653 |  |  |  |  |  |  |
| Pujol et al. 2021 (6) | qPCR | + | 59 | 0 | qPCR +  Q- KK | 95 (87 – 99) | 100 (99 – 100) | 100 (94 – 100) | 100 (99 – 100) | 100 (99 – 100) |
|  |  | - | 3 | 730 |  |  |  |  |  |  |
| Adisakwattana et al. 2020 (7) | qPCR | + | 12 | 0 | qPCR +  D-KK | 92 (64 – 100) | 100 (99 – 100) | 100 (74 – 100) | 100 (99 – 100) | 100 (99 – 100) |
|  |  | - | 1 | 554 |  |  |  |  |  |  |
| Soultani et al. 2024 (8) | qPCR | + | 1114 | 0 | qPCR +  D-KK | 94 (93 – 95) | 100 (100 – 100) | 100 (100 – 100) | 96 (95 – 97) | 98 (97 – 98) |
|  |  | - | 71 | 1789 |  |  |  |  |  |  |
| Keller et al. 2020 (9) | qPCR | + | 372 | 0 | qPCR +  Q-KK | 93 (90 – 95) | 100 (100 – 100) | 100 (99 – 100) | 98 (97 – a98) | 98 (98 – 99) |
|  |  | - | 28 | 1236 |  |  |  |  |  |  |
| Gaidhane et al. 2024 (10) | qPCR | + | 51 | 0 | qPCR +  D-KK | 85 (73 – 93) | 100 (99 – 100) | 100 (93 – 100) | 98 (97 – 99) | 98 (97 – 99) |
|  |  | - | 9 | 474 |  |  |  |  |  |  |
| Aung et al. 2022 (11) | qPCR | + | 145 | 0 | qPCR +  T-KK | 93 (88 – 96) | 100 (97 – 100) | 100 (97 – 100) | 91 (85 – 95) | 96 (93 – 98) |
|  |  | - | 11 | 108 |  |  |  |  |  |  |
| Mugo et al. 2024 (12) | qPCR | + | 58 | 0 | qPCR +  D-KK | 100 (94 – 100) | 100 (99 – 100) | 100 (94 – 100) | 100 (99 – 100) | 100 (99 – 100) |
|  |  | - | 0 | 332 |  |  |  |  |  |  |
| Vlaminck et al. 2019 (13) | qPCR | + | 417 | 0 | qPCR +  D-KK | 98 (96 – 99) | 100 (98 – 100) | 100 (99 – 100) | 96 (93 – 98) | 99 (98 – 99) |
|  |  | - | 9 | 219 |  |  |  |  |  |  |
| Noor et al. 2023(14) | qPCR | + | 220 | 0 | qPCR +  S-KK | 94( 90 – 97) | 100 (98 – 100) | 100 (98 – 100) | 92 (87 – 95) | 96 (94 – 98) |
|  |  | - | 14 | 152 |  |  |  |  |  |  |

qPCR; real-time polymerase chain reaction, , KK; Kato-Katz, CI; confidence interval, S; single; D; duplicate, T; triplicate, Q; quadruplicate

**Table D. Diagnostic accuracy of Kato-Katz compared with a composite reference standard for detecting ascariasis**

| Study details | Index test (KK) | | Reference test (composite of NAAT and KK) | | | Sensitivity % (95%CI) | Specificity % (95%CI) | Positive predictive value % (95%CI) | Negative predictive value % (95%CI) | Accuracy % (95%CI) |
| --- | --- | --- | --- | --- | --- | --- | --- | --- | --- | --- |
|  |  |  | + n | - n | Type |  |  |  |  |  |
| Azzopardi et al. 2021 (1) | D-KK | + | 8 | 0 | qPCR +  D-KK | 89 (52 – 100) | 100 (89 – 100) | 100 (63 – 100) | 97 (83 – 99) | 98 (87 – 100) |
|  |  | - | 1 | 31 |  |  |  |  |  |  |
| Dunn et al. 2020 (2) | S-KK | + | 25 | 0 | qPCR +  S- KK | 53 (38 – 68) | 100 (99 – 100) | 100 (86 – 100) | 97 (95 – 97) | 97 (95 – 98) |
|  |  | - | 22 | 601 |  |  |  |  |  |  |
| Meurs et al. 2017 (3) | S-KK | + | 149 | 0 | qPCR +  S- KK | 92 (87 – 96) | 100 (97 – 100) | 100 (98 – 100) | 92 (87 – 95) | 96 (93 – 98) |
|  |  | - | 13 | 141 |  |  |  |  |  |  |
| Mationg et al. 2014 (4) | T-KK | + | 54 | 0 | qPCR +  T- KK | 30 (24– 38) | 100 (96 – 100) | 100 (93 – 100) | 41 (38 – 43) | 53 (47 – 59) |
|  |  | - | 124 | 85 |  |  |  |  |  |  |
| Easton et al. 2016 (5) | D-KK | + | 103 | 0 | qPCR +  D-KK | 72 (64 – 79) | 100 (99 – 100) | 100 (96 – 100) | 94 (93 – 96) | 95 (93 – 96) |
|  |  | - | 40 | 653 |  |  |  |  |  |  |
| Pujol et al, 2021 (6) | Q-KK | + | 34 | 0 | qPCR +  Q-KK | 55 (42 – 68) | 100 (99 – 100) | 100 (90 – 100) | 96 (95 – 97) | 96 (95 – 98) |
|  |  | - | 28 | 730 |  |  |  |  |  |  |
| Adisakwattana et al. 2020 (7) | D-KK | + | 12 | 0 | qPCR +  D-KK | 92 (64 – 100) | 100 (99 – 100) | 100 (74 – 100) | 100 (99 – 100) | 100 (99 – 100) |
|  |  | - | 1 | 554 |  |  |  |  |  |  |
| Keller et al. 2020 (9) | Q-KK | + | 167 | 0 | qPCR +  Q-KK | 42 (37 – 47) | 100 (100 – 100) | 100 (98 – 100) | 84 (83 – 85) | 86 (84 – 87) |
|  |  | - | 233 | 1236 |  |  |  |  |  |  |
| Soultani et al. 2024 (8) | D-KK | + | 1029 | 0 | qPCR +  D-KK | 87 (85 – 89) | 100 (100 – 100) | 100 (100 – 100) | 92 (91 – 93) | 95 (94 – 96) |
|  |  | - | 156 | 1789 |  |  |  |  |  |  |
| Gaidhane et al. 2024 (10) | D-KK | + | 21 | 0 | qPCR +  D-KK | 35 (23 – 48) | 100 (99 – 100) | 100 (84 – 100) | 92 (91 – 94) | 93 (90 – 95) |
|  |  | - | 39 | 474 |  |  |  |  |  |  |
| Aung et al. 2022 (11) | T-KK | + | 41 | 0 | qPCR +  T-KK | 26 (20 – 34) | 100 (97 – 100) | 100 (91 – 100) | 48 ( 46 – 51) | 56 (50 – 62) |
|  |  | - | 115 | 108 |  |  |  |  |  |  |
| Mugo et al. 2024 (12) | D-KK | + | 36 | 0 | qPCR +  D-KK | 62 (48 – 74) | 100 (99 – 100) | 100 (90 – 100) | 94 (92 – 95) | 94 (92 – 96) |
|  |  | - | 22 | 332 |  |  |  |  |  |  |
| Vlaminck et al. 2019 (13) | D-KK | + | 366 | 0 | qPCR +  D-KK | 86 (82 – 89) | 100 (98 – 100) | 100 (99 – 100) | 78 (73 – 82) | 91 ( 88 – 92) |
|  |  | - | 60 | 209 |  |  |  |  |  |  |
| Noor et al. 2023(14) | S-KK | + | 190 | 0 | qPCR +  S-KK | 81 (76 – 86) | 100 (98 – 100) | 100 (98 – 100) | 76 (73 – 82) | 89 (85 – 92) |
|  |  | - | 44 | 152 |  |  |  |  |  |  |

qPCR; real-time polymerase chain reaction, , KK; Kato-Katz, CI; confidence interval, S; single; D; duplicate, T; triplicate, Q; quadruplicate

**Table E. Diagnostic performance of nucleic acid amplification test compared to the composite reference for detecting trichuriasis**

| Study details | Index test (NAAT) | | Reference test (KK) | | | Sensitivity %  (95%CI) | Specificity % (95%CI) | Positive predictive value % (95%CI) | Negative predictive value % (95%CI) | Accuracy % (95%CI) |
| --- | --- | --- | --- | --- | --- | --- | --- | --- | --- | --- |
|  |  |  | + n | - n | Type |  |  |  |  |  |
| Azzopardi et al. 2021 (1) | qPCR | + | 0 | 0 | qPCR +  D-KK | 0 (0 – 46) | 100 (90 – 100) | – | 85 | 85 (70 –94) |
|  |  | - | 6 | 34 |  |  |  |  |  |  |
| Dunn et al. 2020 (2) | qPCR | + | 148 | 0 | qPCR +  S-KK | 100 (98 – 100) | 100 (99 – 100) | 100 (98 – 100) | 100 (99 – 100) | 100 (99 – 100) |
|  |  | - | 0 | 500 |  |  |  |  |  |  |
| Chung et al. 2020 (15) | qPCR | + | 343 | 0 | qPCR +  D-KK | 92 (89 – 95) | 100 (100 – 100) | 100 (100 – 100) | 99 (98 – 99) | 99 (98 – 99) |
|  |  | - | 28 | 2428 |  |  |  |  |  |  |
| Mationg et al. 2014 (4) | qPCR | + | 102 | 0 | qPCR +  T-KK | 72 (64 – 80) | 100 (97 – 100) | 100 (96 – 100) | 76 (70 – 80) | 85 (80 – 89) |
|  |  | - | 39 | 122 |  |  |  |  |  |  |
| Pujol et al. 2021 (6) | qPCR | + | 103 | 0 | qPCR +  Q-KK | 95 (90 – 98) | 100 (99 – 100) | 100 (96 – 100) | 99 (98 – 100) | 99 (99 – 100) |
|  |  | - | 5 | 684 |  |  |  |  |  |  |
| Adisakwattana et al. 2020 (7) | qPCR | + | 2 | 0 | qPCR +  D-KK | 22 (3 – 56) | 100 (99 – 100) | 100 (16 – 100) | 99 (98 – 99) | 99 (97 – 99) |
|  |  | - | 8 | 557 |  |  |  |  |  |  |
| Soultani et al. 2024 (8) | qPCR | + | 199 | 0 | qPCR +  D-KK | 67 (62 – 73) | 100 (100 – 100) | 100 (98 – 100) | 96 (96 – 97) | 97 (96 – 97) |
|  |  | - | 97 | 2678 |  |  |  |  |  |  |
| Keller et al 2020 (9) | qPCR | + | 1134 | 0 | qPCR +  Q-KK | 89 (87 – 91) | 100 (99 – 100) | 100 (100 – 100) | 72 (69 – 75) | 91 (90 – 93) |
|  |  | - | 140 | 362 |  |  |  |  |  |  |
| Gaidhane et al. 2024 (10) | qPCR | + | 39 | 0 | qPCR +  T-KK | 85 (71 – 94) | 100 (99 – 100) | 100 (91 – 100) | 99 (97 – 99) | 99 (97 – 99) |
|  |  | - | 7 | 488 |  |  |  |  |  |  |
| Ngari et al. 2020 (16) | LAMP | + | 45 | 0 | LAMP +  D-KK | 82 (69 – 91) | 100 (96 – 100) | 100 (92 – 100) | 89 (82 – 94) | 92 (87 – 96) |
|  |  | - | 10 | 82 |  |  |  |  |  |  |
| Aung et al. 2022 (11) | qPCR | + | 177 | 0 | qPCR +  T-KK | 90 (85 – 94) | 100 (95 – 100) | 100 (98 – 100) | 77 (69 – 84) | 92 (89 – 95) |
|  |  | - | 20 | 67 |  |  |  |  |  |  |
| Vlaminck et al. 2019 (13) | qPCR | + | 433 | 0 | qPCR +  D-KK | 97 (95 – 98) | 100 (98 – 100) | 100 (99 – 100) | 93 (89 – 96) | 98 (96 – 98) |
|  |  | - | 14 | 198 |  |  |  |  |  |  |
| Noor et al. 2023(14) | qPCR | + | 299 | 0 | qPCR +  S-KK | 83 (79 – 87) | 100 (87 – 100) | 100 (99 – 100) | 31 (26 – 36) | 84 (80 – 88) |
|  |  | - | 60 | 27 |  |  |  |  |  |  |

qPCR; real-time polymerase chain reaction, KK; Kato-Katz, CI; confidence interval, LAMP; loop-mediated isothermal amplification, S; single; D; duplicate, T; triplicate, Q; quadruplicate

**Table F. Diagnostic performance of Kato-Katz compared to the composite reference for detecting trichuriasis**

| Study details | Index test KK | | Reference test (Composite of NAAT + KK) | | | Sensitivity % (95%CI) | Specificity % (95%CI) | Positive predictive value % (95%CI) | Negative predictive value % (95%CI) | Accuracy % (95%CI) |
| --- | --- | --- | --- | --- | --- | --- | --- | --- | --- | --- |
|  |  |  | + n | - n | Type |  |  |  |  |  |
| Azzopardi et al.2021 (1) | D-KK | + | 6 | 0 | qPCR +  D-KK | 100 (54 – 100) | 100 (90 – 100) | 100 (54 – 100) | 100 (90 – 100) | 100 (91 – 100) |
|  |  | - | 0 | 34 |  |  |  |  |  |  |
| Dunn et al. 2020 (2) | S-KK | + | 82 | 0 | qPCR +  S-KK | 55 (47 -64) | 100 (99 – 100) | 100 (96 -100) | 88 (86 -90) | 90 (87 – 92) |
|  |  | - | 66 | 500 |  |  |  |  |  |  |
| Chung et al. 2020, (15) | D- KK | + | 195 | 0 | qPCR +  D-KK | 53 (47 – 58) | 100 (100 – 100) | 100 (98 – 100) | 93 (93 – 94) | 94 (93 – 95) |
|  |  | - | 176 | 2428 |  |  |  |  |  |  |
| Mationg et al. 2014 (4) | T-KK | + | 62 | 0 | qPCR +  T-KK | 44 (36 –53) | 100 (97 – 100) | 100 (94 – 100) | 61 (57 – 64) | 70 (64 – 75) |
|  |  | - | 79 | 122 |  |  |  |  |  |  |
| Pujol et al. 2021 (6) | Q-KK | + | 52 | 0 | qPCR +  Q-KK | 48 (38 – 58) | 100 (99 – 100) | 100 (93 – 100) | 92 (91 – 94) | 92 (91 – 95) |
|  |  | - | 56 | 684 |  |  |  |  |  |  |
| Adisakwattana et al. 2020 (7) | D-KK | + | 9 | 0 | qPCR +  D-KK | 90 (55 –100) | 100 (99 – 100) | 100 (66 – 100) | 100 (99 – 100) | 100 (99 – 100) |
|  |  | - | 1 | 557 |  |  |  |  |  |  |
| Soultani et al.  2024 (8) | D-KK | + | 182 | 0 | qPCR +  D-KK | 61 (56 – 67) | 100 (100 – 100) | 100 (98 – 100) | 96 (95 – 96) | 96 (95 – 97) |
|  |  | - | 114 | 2678 |  |  |  |  |  |  |
| Keller et al.  2020 (9) | Q-KK | + | 1020 | 0 | qPCR +  Q-KK | 80 (78 – 82) | 100 (99 – 100) | 100 (100 – 100) | 59 (56 – 61) | 84 (83 – 86) |
|  |  | - | 254 | 362 |  |  |  |  |  |  |
| Gaidhane et al. 2024 (10) | T-KK | + | 10 | 0 | qPCR +  T-KK | 22 (11 – 36) | 100 (99 – 100) | 100 (69 –100 ) | 93 (92 – 94) | 93 (91 – 95) |
|  |  | - | 36 | 488 |  |  |  |  |  |  |
| Ngari et al.  2020 (16) | D-KK | + | 44 | 0 | LAMP +  D-KK | 80 (67 – 90) | 100 (96 – 100) | 100 (92 – 100) | 88 (81 – 93) | 92 (86 – 96) |
|  |  | - | 11 | 82 |  |  |  |  |  |  |
| Aung et al. 2022 (11) | T-KK | + | 69 | 0 | qPCR +  T-KK | 35 (28 – 42) | 100 (95 – 100) | 100 (95 – 100) | 34 (32 – 37) | 52 (45 – 57) |
|  |  | - | 128 | 67 |  |  |  |  |  |  |
| Vlaminck et al. 2019 (13) | D-KK | + | 423 | 0 | qPCR +  D-KK | 95 (92 – 97) | 100 (98 – 100) | 100 (99 – 100) | 89 (85 – 92) | 96 (95 – 98) |
|  |  | - | 24 | 198 |  |  |  |  |  |  |
| Noor et al. 2023 (14) | S-KK | + | 336 | 0 | qPCR +  S-KK | 94 (91 -96) | 100 (87 – 100) | 100 (99 – 100) | 54 (45 – 66) | 94 (91 – 96) |
|  |  | - | 23 | 27 |  |  |  |  |  |  |

qPCR; real-time polymerase chain reaction, KK; Kato-Katz, CI; confidence interval, LAMP; loop-mediated isothermal amplification, S; single; D; duplicate, T; triplicate, Q; quadruplicate

**Table G. Diagnostic performance of nucleic acid amplification test compared to the composite reference for detecting hookworm infection**

| Study details | Index test (NAAT) | | Reference test (NAAT + KK) | | | Sensitivity %  (95% CI) | Specificity % (95% CI) | Positive predictive value % (95% CI) | Negative predictive value % (95% CI) | Accuracy %  (95% CI) |
| --- | --- | --- | --- | --- | --- | --- | --- | --- | --- | --- |
|  |  |  | + n | - n | Type |  |  |  |  |  |
| Azzopardi et al. 2021 (1) | qPCR | + | 14 | 0 | qPCR + D-KK | 82 (57 – 96) | 100 (85 – 100) | 100 (77 – 100) | 88.5 (73 – 96) | 93 (80 – 98) |
|  |  | - | 3 | 23 |  |  |  |  |  |  |
| Dunn et al. 2020 (2) | qPCR | + | 166 | 0 | qPCR + S-KK | 98 (95 – 100) | 100 (99 – 100) | 100 (98 – 100) | 99 (98 – 100) | 100 (99 – 100) |
|  |  | - | 0 | 482 |  |  |  |  |  |  |
| Chung et al. 2020 (15) | qPCR | + | 598 | 0 | qPCR + D-KK | 98 (96 – 99) | 100 (100 – 100) | 100 (99 – 100) | 99 (99 – 100) | 100 (99 – 100) |
|  |  | - | 14 | 2187 |  |  |  |  |  |  |
| Mationg et al. 2014 (4) | qPCR | + | 18 | 0 | qPCR + T-KK | 100 (81 – 100) | 100 (99 – 100) | 100 (81 – 100) | 100 (98 – 100) | 100 (99 – 100) |
|  |  | - | 0 | 245 |  |  |  |  |  |  |
| Pujol et al. 2021 (6) | qPCR | + | 224 | 0 | qPCR + Q-KK | 95 (91 – 97) | 100 (99 – 100) | 100 (98 – 100) | 97 (96 – 99) | 98 (97 – 99) |
|  |  | - | 12 | 556 |  |  |  |  |  |  |
| Adisakwattana et al. 2020 (7) | qPCR | + | 23 | 0 | qPCR + D-KK | 59 (42 – 74) | 100 (99 – 100) | 100 (85 – 100) | 97 (96 – 98) | 97 (95 – 98) |
|  |  | - | 16 | 528 |  |  |  |  |  |  |
| Soultani et al.  2024 (8) | qPCR | + | 334 | 0 | qPCR + D-KK | 96 (94 – 98) | 100 (100 – 100) | 100 (99 – 100) | 100 (99 – 100) | 100 (99 – 100) |
|  |  | - | 13 | 2627 |  |  |  |  |  |  |
| Keller et al.  2020 (9) | qPCR | + | 249 | 0 | qPCR + Q-KK | 93 (90 – 96) | 100 (100 – 100) | 100 (99 – 100) | 99 (98 – 99) | 99 (98 – 99) |
|  |  | - | 18 | 1369 |  |  |  |  |  |  |
| Gaidhane et al. 2024 (10) | qPCR | + | 19 | 0 | qPCR + T-KK | 95 (75 – 100) | 100 (99 – 100) | 100 (82 – 100) | 100 (99 – 100) | 100 (99 – 100) |
|  |  | - | 1 | 514 |  |  |  |  |  |  |
| Knopp et al. 2014 (17) | qPCR | + | 55 | 0 | qPCR + D-KK | 78 (67 – 87) | 100 (97 – 100) | 100 (93 – 100) | 90 (86 – 94) | 93 (89– 96) |
|  |  | - | 15 | 145 |  |  |  |  |  |  |
| Meurs et al. 2017 (3) | qPCR | + | 106 | 0 | qPCR + S-KK | 97 (92 – 99) | 100 (98 – 100) | 100 (97 – 100) | 98 (95 – 100) | 99 (97 – 100) |
|  |  | - | 3 | 194 |  |  |  |  |  |  |
| Schär et al. 2013 (18) | qPCR | + | 76 | 0 | qPCR + D-KK | 87 (79 – 94) | 100 (97 -100) | 100 (95 – 100) | 92 (87 – 95) | 95 (91 – 98) |
|  |  | - | 11 | 131 |  |  |  |  |  |  |
| Mens et al. 2013 (19) | qPCR | + | 96 | 0 | qPCR + D-KK | 97 (91 – 100) | 100 (87 – 100) | 100 (96 – 100) | 90 (74 -96) | 98 (93 – 100) |
|  |  | - | 3 | 26 |  |  |  |  |  |  |
| Inpankaew et al. 2014 (20) | qPCR | + | 115 | 0 | qPCR + Q-KK | 100 (97.3 – 100) | 100 (94.9 – 100) | 100 (97.3 – 100) | 100 (94.9 – 100) | 100 (98.2 – 100) |
|  |  | - | 4 | 86 |  |  |  |  |  |  |
| Easton et al. 2016) (5) | qPCR | + | 146 | 0 | qPCR + Q-KK | 97 (93 – 99) | 100 (100 – 100) | 100 (98 – 100) | 100 (99 – 100) | 100 (99 – 100) |
|  |  | - | 4 | 826 |  |  |  |  |  |  |
| Aung et al. 2022 (11) | qPCR | + | 80 | 0 | qPCR + T-KK | 100 (95 – 100) | 100 (98 – 100) | 100 (95 – 100) | 100 (98 – 100) | 100 (99 – 100) |
|  |  | - | 0 | 184 |  |  |  |  |  |  |
| Vlaminck et al. 2019 (13) | qPCR | + | 432 | 0 | qPCR + D-KK | 99 (97 – 100) | 100 (98 – 100) | 100 (99 – 100) | 98 ( 95 – 99) | 99 (98 – 100) |
|  |  | - | 5 | 208 |  |  |  |  |  |  |
| Noor et al. 2023 (14) | qPCR | + | 115 | 0 | qPCR + S-KK | 97 (92 – 99) | 100 (96 – 100) | 100 (97 – 100) | 96 (89 – 98) | 98 (95 – 99) |
|  |  | - | 4 | 86 |  |  |  |  |  |  |

qPCR; real-time polymerase chain reaction, , KK; Kato-Katz, CI; confidence interval, S; single; D; duplicate, T; triplicate, Q; quadruplicate

**Table H. Diagnostic performance of Kato-Katz compared to the composite reference for detecting hookworm infection**

| Study details | Index test (KK) | | Reference test  (NAAT + KK) | | | Sensitivity % (95% CI) | Specificity % (95% CI) | Positive predictive value % (95% CI) | Negative predictive value % (95% CI) | Accuracy %  (95% CI) |
| --- | --- | --- | --- | --- | --- | --- | --- | --- | --- | --- |
|  |  |  | + n | - n | Type |  |  |  |  |  |
| Azzopardi et al. 2021 (1) | D-KK | + | 9 | 0 | qPCR + D-KK | 53 (28 – 77) | 100 (85 – 100) | 100 (66 -100) | 74 (64 – 83) | 80 (64 – 90) |
|  |  | - | 8 | 23 |  |  |  |  |  |  |
| Dunn et al. 2020 (2) | S-KK | + | 42 | 0 | qPCR + S-KK | 25 (19 – 33) | 100 (99 – 100) | 100 (92 – 100) | 80 (78 – 81) | 81 (77 – 84) |
|  |  | - | 124 | 482 |  |  |  |  |  |  |
| Chung et al. 2020 (15) | D-KK | + | 209 | 0 | qPCR + D-KK | 34 (30 – 38) | 100 (100 – 100) | 100 (98 – 100) | 85 (84 – 85) | 86 (84 – 87) |
|  |  | - | 403 | 2187 |  |  |  |  |  |  |
| Mationg et al. 2014 (4) | T-KK | + | 0 | 0 | qPCR + T-KK | 0 (0 –19) | 100 (99 – 100) | – | 93 (93 – 93) | 93 (89 – 96) |
|  |  | - | 18 | 245 |  |  |  |  |  |  |
| Pujol et al. 2021 (6) | Q-KK | + | 114 | 0 | qPCR + Q-KK | 48 (42 – 55) | 100 (99 – 100) | 100 (97 – 100) | 82 (80 – 84) | 85 (82 – 87) |
|  |  | - | 122 | 556 |  |  |  |  |  |  |
| Adisakwattana et al. 2020 (7) | D-KK | + | 33 | 0 | qPCR + D-KK | 85 (69 – 94) | 100 (99 – 100) | 100 (89 – 100) | 99 (98 – 100) | 100 (98 – 100) |
|  |  | - | 6 | 528 |  |  |  |  |  |  |
| Soultani et al. 2024 (8) | D-KK | + | 81 | 0 | qPCR + D-KK | 23 (19 – 28) | 100 (100 – 100) | 100 (96 – 100) | 91 (90 – 91) | 91 (90 – 92) |
|  |  | - | 266 | 2627 |  |  |  |  |  |  |
| Gaidhane et al. 2024 (10) | T-KK | + | 4 | 0 | qPCR + T-KK | 20 (6 – 44) | 100 (99 – 100) | 100 (40 – 100) | 97 (96 – 98) | 97 (95 – 98) |
|  |  | - | 16 | 514 |  |  |  |  |  |  |
| Knopp et al. 2014 (17) | D-KK | + | 55 | 0 | RT-PCR + D-KK | 79 (67 – 87) | 100 (98 – 100) | 100 (94 – 100) | 91 (86 – 94) | 93 (89 – 96) |
|  |  | - | 15 | 145 |  |  |  |  |  |  |
| Meurs et al. 2017 (3) | S-KK | + | 79 | 0 | qPCR + S-KK | 72 (63 – 81) | 100 (98 – 100) | 100 (95 – 100) | 87 (83 – 90) | 90 (86 – 93) |
|  |  | - | 30 | 194 |  |  |  |  |  |  |
| Schär et al. 2013 (18) | D-KK | + | 52 | 0 | qPCR + D-KK | 60 (50 – 70) | 100 (97 – 100) | 100 (93 – 100) | 78.9 (74 – 83) | 84 (78 – 89) |
|  |  | - | 35 | 131 |  |  |  |  |  |  |
| Mens et al. 2013 (19) | D-KK | + | 79 | 0 | qPCR + D-KK | 80 (71 – 87) | 100 (96 – 100) | 100 (95 – 100) | 83 (76 – 88) | 90 (85 – 92) |
|  |  | - | 20 | 96 |  |  |  |  |  |  |
| Inpankaew et al. 2014 (20) | Q-KK | + | 45 | 0 | qPCR + Q-KK | 38 (29 – 47) | 100 (96 – 100) | 100 (92 – 100) | 54 (50 – 57) | 64 (57 – 70) |
|  |  | - | 74 | 86 |  |  |  |  |  |  |
| Easton et al, (2016) (5) | Q-KK | + | 57 | 0 | qPCR + Q-KK | 38 (30 – 46) | 100 (100 – 100) | 100 (94 – 100) | 90 (89 – 91) | 90 (88 – 92) |
|  |  | - | 93 | 826 |  |  |  |  |  |  |
| Aung et al. 2022 (11) | T-KK | + | 0 | 0 | qPCR+ T-KK | 0 (0 – 5) | 100 (98 – 100) | 30 (25 – 36) | 70 (70 – 70) | 70 (64 – 75) |
|  |  | - | 80 | 184 |  |  |  |  |  |  |
| Keller et al. 2020 (9) | Q-KK | + | 107 | 0 | qPCR+ Q-KK | 40 (34 – 46) | 100 (100 – 100) | 100 (97 – 100) | 90 (88 – 90) | 90 (90 – 92) |
|  |  | - | 160 | 1369 |  |  |  |  |  |  |
| Vlaminck et al. 2019 (13) | D-KK | + | 372 | 0 | qPCR+ D-KK | 85 (82 – 89) | 100 (98 – 100) | 100 (99 – 100) | 77 (72 – 81) | 90 (88 – 92) |
|  |  | - | 63 | 208 |  |  |  |  |  |  |
| Noor et al. 2023 (14) | S-KK | + | 32 | 0 | qPCR+ S-KK | 65 (50 – 78) | 100 (99 – 100) | 100 (89 – 100) | 95 (93 – 97) | 96 (93 – 97) |
|  |  | - | 17 | 337 |  |  |  |  |  |  |

qPCR; real-time polymerase chain reaction, , KK; Kato-Katz, CI; confidence interval, S; single; D; duplicate, T; triplicate, Q; quadruplicate

**Strongyloidiasis**

**Table I. Diagnostic performance of the nucleic acid amplification test compared to the composite reference for the detection of strongyloidiasis**

| Study details | Index test (NAAT) | | Reference test (NAAT + BT) | | Sensitivity %  (95% CI) | Specificity % (95% CI) | Positive predictive value % (95% CI) | Negative predictive value % (95% CI) | Accuracy %  (95% CI) |
| --- | --- | --- | --- | --- | --- | --- | --- | --- | --- |
|  |  |  | + n | - n |  |  |  |  |  |
| Chankongsin et al. 2020 (21) | qPCR | + | 26 | 0 | 90 (73 – 98) | 100 (95 – 100) | 100 (87 – 100) | 96 (90 – 99) | 97 (92 – 99) |
|  |  | - | 3 | 75 |  |  |  |  |  |
| Hailu et al. 2022 (22) | qPCR | + | 243 | 0 | 82 (77 – 86) | 100 (99 – 100) | 100 (99 – 100) | 91 (89 – 93) | 94 (92 – 95) |
|  |  | - | 53 | 548 |  |  |  |  |  |
| Becker et al. 2015 (23) | qPCR | + | 43 | 0 | 77 (64 – 87) | 100 (98 – 100) | 100 (92 – 100) | 94 (91 – 96) | 95 (92 – 97) |
|  |  | - | 13 | 200 |  |  |  |  |  |
| Barda et al. 2018 (24) | qPCR | + | 87 | 0 | 100 (96 – 100) | 100 (63 – 100) | 100 (96 – 100) | 100 (63 – 100) | 100 (96 – 100) |
|  |  | - | 0 | 8 |  |  |  |  |  |
| Knopp et al. 2014 (17) | qPCR | + | 17 | 0 | 31 (19 – 45) | 100 (97 – 100) | 100 (81 – 100) | 78 (75 – 81) | 80 (74 – 86) |
|  |  | - | 38 | 138 |  |  |  |  |  |
| Meurs et al. 2017 (3) | qPCR | + | 134 | 0 | 95 (90 – 98) | 100 (98 – 100) | 100 (97 – 100) | 96.3 (92 – 98) | 98 (95 – 99) |
|  |  | - | 7 | 162 |  |  |  |  |  |
| Schär et al. 2013 (18) | qPCR | + | 88 | 0 | 100 (96 – 100) | 100 (98 – 100) | 100 (96 – 100) | 100 (98 – 100) | 100 (99 – 100) |
|  |  | - | 0 | 177 |  |  |  |  |  |
| Malaga et al (2024) (25) | qPCR | + | 114 | 0 | 100 (97 – 100) | 100 (98 – 100) | 100 (97 – 100) | 100 (98 – 100) | 100 (99 – 100) |
|  |  | - | 0 | 186 |  |  |  |  |  |
| Kristanti et al. 2018 (26) | cPCR | + | 45 | 0 | 79 (66 – 89) | 100 (85 – 100) | 100 (92 – 100) | 66 (54 – 76) | 85 (75 – 92) |
|  |  | - | 12 | 23 |  |  |  |  |  |
| Amor et al. 2016 (27) | qPCR | + | 53 | 0 | 65 (54 – 76) | 100 (99 – 100) | 100 (93 – 100) | 92 (89 – 94) | 93 (90 – 95) |
|  |  | - | 28 | 315 |  |  |  |  |  |
| Fleitas et al. 2021(28) | qPCR | + | 110 | 0 | 97(92 – 99) | 100 (91 – 100) | 100 (96 – 100) | 93 (80 – 97) | 98 (94 – 100) |
|  |  | - | 3 | 38 |  |  |  |  |  |
| Amor et al. 2020 (29) | qPCR | + | 287 | 0 | 66 (61 – 71) | 100 (99 – 100) | 100 (99 – 100) | 71 (68 – 74) | 81 (79 – 84) |
|  |  | - | 147 | 358 |  |  |  |  |  |

qPCR; real-time polymerase chain reaction, cPCR; conventional polymerase chain reaction, BT; Baerman test, CI; confidence interval

**Table J. Diagnostic performance of the Baerman test compared to the composite reference for the detection of strongyloidiasis**

| Study details | Index test (BT) | | Reference test (NAAT+BT) | | Sensitivity %  (95% CI) | Specificity % (95% CI) | Positive predictive value % (95% CI) | Negative predictive value % (95% CI) | Accuracy %  (95% CI) |
| --- | --- | --- | --- | --- | --- | --- | --- | --- | --- |
|  |  |  | + n | - n |  |  |  |  |  |
| Chankongsin et al. 2020 (21) | BT | + | 21 | 0 | 72 (53 – 87) | 100 (95 – 100) | 100 (84 – 100) | 90 (84 – 94) | 92 (85 – 97) |
|  |  | - | 8 | 75 |  |  |  |  |  |
| Hailu et al. 2022 (22) | BT | + | 87 | 0 | 29 (24 – 35) | 100 (99 – 100) | 100 (96 – 100) | 72 (71 – 74) | 75 (72 – 78) |
|  |  | - | 209 | 548 |  |  |  |  |  |
| Becker et al. 2015 (23) | BT | + | 21 | 0 | 38 (25 – 51) | 100 (98 – 100) | 100 (84 – 100) | 85 (82 – 88) | 86 (82 – 90) |
|  |  | - | 35 | 200 |  |  |  |  |  |
| Barda et al. 2018 (24) | BT | + | 69 | 0 | 79 (69 – 87) | 100 (63 – 100) | 100 (95 – 100) | 31 (23 – 40) | 81 (72 – 88) |
|  |  | - | 18 | 8 |  |  |  |  |  |
| Knopp et al. 2014 (17) | BT | + | 46 | 0 | 84 (71 – 92) | 100 (97 – 100) | 100 (92 – 100) | 94 (89 – 97) | 95 (91 – 98) |
|  |  | - | 9 | 138 |  |  |  |  |  |
| Meurs et al .2017 (3) | BT | + | 70 | 0 | 50 (41 – 58) | 100 (98 – 100) | 100 (95 – 100) | 70 (66 – 73) | 77 (71 – 81) |
|  |  | - | 71 | 162 |  |  |  |  |  |
| Schär et al. 2013 (18) | BT | + | 24 | 0 | 77 (59 – 90) | 100 (98 – 100) | 100 (86 – 100) | 96 (93 – 98) | 97 (93 – 99) |
|  |  | - | 7 | 177 |  |  |  |  |  |
| Malaga et al. 2024 (25) | BT | + | 38 | 0 | 33( 25 – 43) | 100 (98 – 100) | 100 (91 – 100) | 71 (68 – 74) | 75 (69 – 80) |
|  |  | - | 76 | 186 |  |  |  |  |  |
| Kristanti et al. 2018 (26) | BT | + | 40 | 0 | 70 (57 – 82) | 100 (85 – 100) | 100 (91 – 100) | 58 (48 – 67) | 79 (68 – 87) |
|  |  | - | 17 | 23 |  |  |  |  |  |
| Amor et al. 2016 (27) | BT | + | 50 | 0 | 62 (50 – 72) | 100 (99 – 100) | 100 (93 – 100) | 91 (89 – 93) | 92 (89 – 95) |
|  |  | - | 31 | 315 |  |  |  |  |  |
| Fleitas et al. 2021(28) | BT | + | 54 | 0 | 48 (38 – 57) | 100 (91 – 100) | 100 (93 – 100) | 40 (35 – 43) | 61 (53 – 69) |
|  |  | - | 59 | 38 |  |  |  |  |  |
| Amor et al. 2020 (29) | BT | + | 262 | 0 | 60 (56 – 65) | 100 (99 – 100) | 100 (99 – 100) | 68 ( 65 – 70) | 78 (75 – 81) |
|  |  | - | 172 | 358 |  |  |  |  |  |

qPCR; real-time polymerase chain reaction, BT; Baerman test, CI; confidence interval

**Soil-transmitted helminths (*Ascaris*, *Trichuris* and hookworms)**

**Table K. Diagnostic performance of the nucleic acid amplification test compared to composite reference for the detection of soil-transmitted helminths**

| Study details | Index test (NAAT) | | Reference test  (RT-PCR + KK) | | | Sensitivity % (95% CI) | Specificity % (95% CI) | Positive predictive value % (95% CI) | Negative predictive value % (95% CI) | Accuracy %  (95% CI) |
| --- | --- | --- | --- | --- | --- | --- | --- | --- | --- | --- |
|  |  |  | + n | - n | Type |  |  |  |  |  |
| Azzopardi et al 2021 (1) | qPCR | + | 18 | 0 | qPCR  + D-KK | 69(48 – 86) | 100 (77 – 100) | 100 (82 – 100) | 64 (50 – 76) | 80 (64 – 91) |
|  |  | - | 8 | 14 |  |  |  |  |  |  |
| Mationg et al 2014 (4) | qPCR | + | 206 | 0 | qPCR  + T-KK | 94 (90 – 97) | 100 (92 – 100) | 100 (98 – 100) | 77 (67 – 85) | 95 (92 – 97) |
|  |  | - | 13 | 44 |  |  |  |  |  |  |
| Adisakwattana et al 2020 (7) | qPCR | + | 39 | 0 | qPCR  + D-KK | 76 (61 – 86) | 100 (99 – 100) | 100 (91 – 100) | 98 (96 – 98) | 98 (96 – 99) |
|  |  | - | 13 | 515 |  |  |  |  |  |  |
| Soultani et al 2024 (8) | qPCR | + | 1365 | 0 | qPCR  + D-KK | 96 (94 – 97) | 100 (100 – 100) | 100 (100 – 100) | 96 (95 – 97) | 98 (97 – 98) |
|  |  | - | 64 | 1545 |  |  |  |  |  |  |
| Gaidhane et al 2024 (10) | qPCR | + | 109 | 0 | qPCR  + T-KK | 91 (84 – 95) | 100 (99 – 100) | 100 (97 – 100) | 97 (96 – 98) | 98 (96 – 99) |
|  |  | - | 11 | 414 |  |  |  |  |  |  |
| Aung et al. 2022 (11) | T-KK | +  - | 208 | 0 | qPCR  + T-KK | 94 (90 – 97) | 100 (92 – 100) | 100 (98 – 100) | 75 (64 – 83) | 95 (91 – 97) |
|  |  |  | 14 | 42 |  |  |  |  |  |  |

qPCR; real-time polymerase chain reaction, KK, Kato-Katz, CI; confidence interval

**Table L. Diagnostic performance of the Kato-Katz test compared to composite reference for the detection of soil-transmitted helminths**

| Study details | Index test (KK) | | Reference test  (NAAT+ KK) | | | Sensitivity %  (95% CI) | Specificity % (95% CI) | Positive predictive value % (95% CI) | Negative predictive value % (95% CI) | Accuracy %  (95% CI) |
| --- | --- | --- | --- | --- | --- | --- | --- | --- | --- | --- |
|  |  |  | + n | - n | Type |  |  |  |  |  |
| Azzopardi et al. 2021 (1) | D-KK | + | 18 | 0 | qPCR +  D-KK | 69(48 – 86) | 100 (77 – 100) | 100 (82 – 100) | 64 (50 – 76) | 80 (64 – 91) |
|  |  | - | 8 | 14 |  |  |  |  |  |  |
| Mationg et al. 2014 (4) | T-KK | + | 89 | 0 | qPCR +  T-KK | 41 (34 – 48) | 100 (92 – 100) | 100 (96 – 100) | 25 (23 – 27) | 51 (44 – 57) |
|  |  | - | 130 | 44 |  |  |  |  |  |  |
| Adisakwattana et al. 2020 (7) | D-KK | + | 23 | 0 | qPCR +  D-KK | 44 (30 –59 ) | 100 (99 – 100) | 100 (85 – 100) | 95 (94 – 96) | 95 (93 – 97) |
|  |  | - | 29 | 545 |  |  |  |  |  |  |
| Soultani et al.  2024 (8) | D-KK | + | 1142 | 0 | qPCR +  D-KK | 80 (78 -92) | 100 (100 – 100) | 100 (100 – 100) | 84 (83 – 85) | 90 (89 – 91) |
|  |  | - | 287 | 1545 |  |  |  |  |  |  |
| Gaidhane et al. 2024 (10) | T-KK | + | 35 | 0 | qPCR +  T-KK | 29 (21 – 38) | 100 (99 – 100) | 100 (90 – 100) | 83 (81 – 85) | 84 (81 – 87) |
|  |  | - | 85 | 414 |  |  |  |  |  |  |
| Aung et al. 2022 (11) | T-KK | + | 88 | 0 | qPCR +  T-KK | 40 (33 – 46) | 100 (92 – 100) | 100 (96 – 100) | 24 (22 – 26) | 49 (43 – 55) |
|  |  | - | 134 | 42 |  |  |  |  |  |  |

qPCR; real-time polymerase chain reaction, KK, Kato-Katz, CI; confidence interval

**References**

1. Azzopardi KI, Hardy M, Baker C, Bonnici R, Llewellyn S, McCarthy JS, Traub RJ, Steer AC. Detection of six soil-transmitted helminths in human stool by qPCR- a systematic workflow. PLoS One. 2021 Sep 30;16(9):e0258039. doi: 10.1371/journal.pone.0258039.

2. Dunn JC, Papaiakovou M, Han KT, Chooneea D, Bettis AA, Wyine NY, et al. The increased sensitivity of qPCR in comparison to Kato-Katz is required for the accurate assessment of the prevalence of soil-transmitted helminth infection in settings that have received multiple rounds of mass drug administration. Parasites Vectors 13, 324 (2020). doi: org/10.1186/s13071-020-04197-w

3. Meurs L, Polderman AM, Vinkeles Melchers NV, Brienen EA, Verweij JJ, Groosjohan B, Mendes F, Mechendura M, Hepp DH, Langenberg MC, Edelenbosch R, Polman K, van Lieshout L. Diagnosing Polyparasitism in a High-Prevalence Setting in Beira, Mozambique: Detection of intestinal parasites in fecal samples by microscopy and real-time PCR. PLoS Negl Trop Dis. 2017 Jan 23;11(1):e0005310. doi: 10.1371/journal.pntd.0005310.

4. Mationg MLS, Gordon CA, Tallo VL, Olveda RM, Alday PP, Reñosa MDC, et al. Status of soil-transmitted helminth infections in schoolchildren in Laguna Province, the Philippines: Determined by parasitological and molecular diagnostic techniques. PLoS Negl Trop Dis. 2017 Nov 6;11(11):e0006022. doi: 10.1371/journal.pntd.0006022.

5. Easton A V., Oliveira RG, O’Connell EM, Kepha S, Mwandawiro CS, Njenga SM, et al. Multi-parallel qPCR provides increased sensitivity and diagnostic breadth for gastrointestinal parasites of humans: field-based inferences on the impact of mass deworming. Parasit Vectors. 2016 Jan 27;9:38. doi: 10.1186/s13071-016-1314-y.

6. Grau-Pujol B, Martí-Soler H, Escola V, Demontis M, Jamine JC, Gandasegui J, et al. Towards soil-transmitted helminths transmission interruption: The impact of diagnostic tools on infection prediction in a low intensity setting in Southern Mozambique. PLoS Negl Trop Dis. 2021 Oct 25;15(10):e0009803. doi: 10.1371/journal.pntd.0009803.

7. Adisakwattana P, Yoonuan T, Phuphisut O, Poodeepiyasawat A, Homsuwan N, Gordon CA, et al. Clinical helminthiases in Thailand border regions show elevated prevalence levels using qPCR diagnostics combined with traditional microscopic methods. Parasit Vectors. 2020 Aug 12;13(1):416. doi: 10.1186/s13071-020-04290-0.

8. Soultani M, Bartlett AW, Mendes EP, Hii SF, Traub R, Palmeirim MS, et al. Estimating prevalence and infection intensity of soil-transmitted helminths using quantitative polymerase chain reaction and Kato-Katz in school-age children in Angola. Am J Trop Med Hyg. 2024 Apr 30;110(6):1145-1151. doi: 10.4269/ajtmh.23-0821.

9. Keller L, Patel C, Welsche S, Schindler T, Hürlimann E, Keiser J. Performance of the Kato-Katz method and real time polymerase chain reaction for the diagnosis of soil-transmitted helminthiasis in the framework of a randomised controlled trial: treatment efficacy and day-to-day variation. Parasit Vectors. 2020 Oct 15;13(1):517. doi: 10.1186/s13071-020-04401-x.

10. Gaidhane S, Gaidhane A, Khatib MN, Telrandhe S, Patil M, Saxena D, et al. Estimation of the parasitic burden of soil-transmitted helminths among pregnant women in the Maharashtra state of India using qPCR: A community-based study. Indian J Community Med. 2024 Jan-Feb;49(1):157-164. doi: 10.4103/ijcm.ijcm_249_23.

11. Aung E, Han KT, Gordon CA, Hlaing NN, Aye MM, Htun MW, et al. High prevalence of soil-transmitted helminth infections in Myanmar schoolchildren. Infect Dis Poverty. 2022 Mar 10;11(1):28. doi: 10.1186/s40249-022-00952-6.

12. Mugo RM, Rausch S, Musimbi ZD, Strube C, Raulf M-K, Landt O, et al. Evaluation of copromicroscopy, multiplex-qPCR and antibody serology for monitoring of human ascariasis in endemic settings. PLoS Negl Trop Dis. 2024 Jun 18;18(6):e0012279. doi: 10.1371/journal.pntd.0012279.

13. Poole C, Barker T, Bradbury R, Capone D, Chatham AH, Handali S, et al. Cross-sectional study of soil-transmitted helminthiases in black belt region of Alabama, USA. Emerg Infect Dis. 2023 Dec;29(12):2461-2470. doi: 10.3201/eid2912.230751.

14. Noor Z, Hossain B, Khan SS, Kabir M, Bhuiyan ATMRH, Alam MS, et al. Prevalence of soil-transmitted helminths at baseline and after albendazole treatment in the school-age children of forcibly displaced Myanmar nationals in Bangladesh. Am J Trop Med Hyg. 2023 Aug 7;109(3):656-666. doi: 10.4269/ajtmh.23-0260.

15. Chung JB, Pilotte N, Ercumen A, Grant JR, Maasch JRMA, Gonzalez AM, Ester AC, Arnold BF, Rahman M, Haque R, Hubbard AE, Luby SP, Williams SA, Colford JM Jr. Comparison of multi-parallel qPCR and double-slide Kato-Katz for detection of soil-transmitted helminth infection among children in rural Bangladesh. PLoS Negl Trop Dis. 2020 Apr 24;14(4):e0008087. doi: 10.1371/journal.pntd.0008087.

16. Ngari MG, Mwangi IN, Njoroge MP, Kinyua J, Osuna FA, Kimeu BM, et al. Development and evaluation of a loop-mediated isothermal amplification (LAMP) diagnostic test for detection of whipworm, Trichuris trichiura, in faecal samples. J Helminthol. 2020 Apr 2;94:e142. doi: 10.1017/S0022149X2000022X.

17. Knopp S, Salim N, Schindler T, Voules DAK, Rothen J, Lweno O, et al. Diagnostic accuracy of Kato-Katz, FLOTAC, Baermann, and PCR methods for the detection of light-intensity hookworm and Strongyloides stercoralis infections in Tanzania. Am J Trop Med Hyg. 2014 Mar;90(3):535-545. doi: 10.4269/ajtmh.13-0268.

18. Schär F, Odermatt P, Khieu V, Panning M, Duong S, Muth S, Marti H, Kramme S. Evaluation of real-time PCR for *Strongyloides stercoralis* and hookworm as diagnostic tool in asymptomatic schoolchildren in Cambodia. Acta Trop. 2013 May;126(2):89-92. doi: 10.1016/j.actatropica.2012.12.012.

19. van Mens SP, Aryeetey Y, Yazdanbakhsh M, van Lieshout L, Boakye D, Verweij JJ. Comparison of real-time PCR and Kato smear microscopy for the detection of hookworm infections in three consecutive faecal samples from schoolchildren in Ghana. Trans R Soc Trop Med Hyg. 2013 Apr;107(4):269-71. doi: 10.1093/trstmh/trs094.

20. Inpankaew T, Schär F, Khieu V, Muth S, Dalsgaard A, Marti H, Traub RJ, Odermatt P. Simple fecal flotation is a superior alternative to quadruple Kato-Katz smear examination for the detection of hookworm eggs in human stool. PLoS Negl Trop Dis. 2014 Dec 18;8(12):e3313. doi: 10.1371/journal.pntd.0003313.

21. Chankongsin S, Wampfler R, Ruf MT, Odermatt P, Marti H, Nickel B, Keoluangkhot V, Neumayr A. *Strongyloides stercoralis* prevalence and diagnostics in Vientiane, Lao People's Democratic Republic. Infect Dis Poverty. 2020 Sep 21;9(1):133. doi: 10.1186/s40249-020-00750-y.

22. Hailu, T., Amor, A., Nibret, E. et al. Evaluation of five diagnostic methods for *Strongyloides stercoralis* infection in Amhara National Regional State, northwest Ethiopia. BMC Infect Dis 22, 297 (2022). doi: 10.1186/s12879-022-07299-1

23. Becker SL, Piraisoody N, Kramme S, Marti H, Silué KD, Panning M, Nickel B, Kern WV, Herrmann M, Hatz CF, N'Goran EK, Utzinger J, von Müller L. Real-time PCR for detection of *Strongyloides stercoralis* in human stool samples from Côte d'Ivoire: diagnostic accuracy, inter-laboratory comparison and patterns of hookworm co-infection. Acta Trop. 2015 Oct;150:210-7. doi: 10.1016/j.actatropica.2015.07.019.

24. Barda B, Wampfler R, Sayasone S, Phongluxa K, Xayavong S, Keoduangsy K, Schindler C, Keiser J. Evaluation of Two DNA Extraction Methods for Detection of *Strongyloides stercoralis* Infection. J Clin Microbiol. 2018 Mar 26;56(4):e01941-17. doi: 10.1128/JCM.01941-17.

25. Malaga JL, Fernandez-Baca M V, Castellanos-Gonzalez A, Tanabe MB, Tift C, Morales ML, et al. The Recombinase polymerase amplification test for *Strongyloides stercoralis* is more sensitive than microscopy and real-time PCR in high-risk communities of Cusco, Peru. Pathogens. 2024 Oct 3;13(10):869. doi: 10.3390/pathogens13100869.

26. Kristanti H, Meyanti F, Wijayanti MA, Mahendradhata Y, Polman K, Chappuis F, et al. Diagnostic comparison of Baermann funnel, Koga agar plate culture and polymerase chain reaction for detection of human Strongyloides stercoralis infection in Maluku, Indonesia. Parasitol Res. 2018 Oct;117(10):3229-3235. doi: 10.1007/s00436-018-6021-5.

27. Amor A, Rodriguez E, Saugar JM, Arroyo A, López-Quintana B, Abera B, et al. High prevalence of *Strongyloides stercoralis* in school-aged children in a rural highland of north-western Ethiopia: the role of intensive diagnostic work-up. Parasit Vectors. 2016 Dec 1;9(1):617. doi: 10.1186/s13071-016-1912-8.

28. Fleitas PE, Vargas PA, Caro N, Almazan MC, Echazú A, Juárez M, et al. Scope and limitations of a multiplex conventional PCR for the diagnosis of *S. stercoralis* and hookworms. Braz J Infect Dis. 2021 Nov-Dec;25(6):101649. doi: 10.1016/j.bjid.2021.101649.

29. Aramendia AA, Anegagrie M, Zewdie D, Dacal E, Saugar JM, Herrador Z, et al. Epidemiology of intestinal helminthiases in a rural community of Ethiopia: Is it time to expand control programs to include *Strongyloides stercoralis* and the entire community? PLoS Negl Trop Dis. 2020 Jun 4;14(6):e0008315. doi: 10.1371/journal.pntd.0008315.
