# Supplementary material for: Diagnostic accuracy of nucleic acid amplification tests for human intestinal nematode infections: A systematic review and meta-analysis
Source: PLoS Negl Trop Dis. 2026 Feb 11;20(2):e0013974. doi: 10.1371/journal.pntd.0013974 (PMC12916058; doi:10.1371/journal.pntd.0013974)
Supplement: S2 Appendix — (DOCX) [file pntd.0013974.s005.docx]

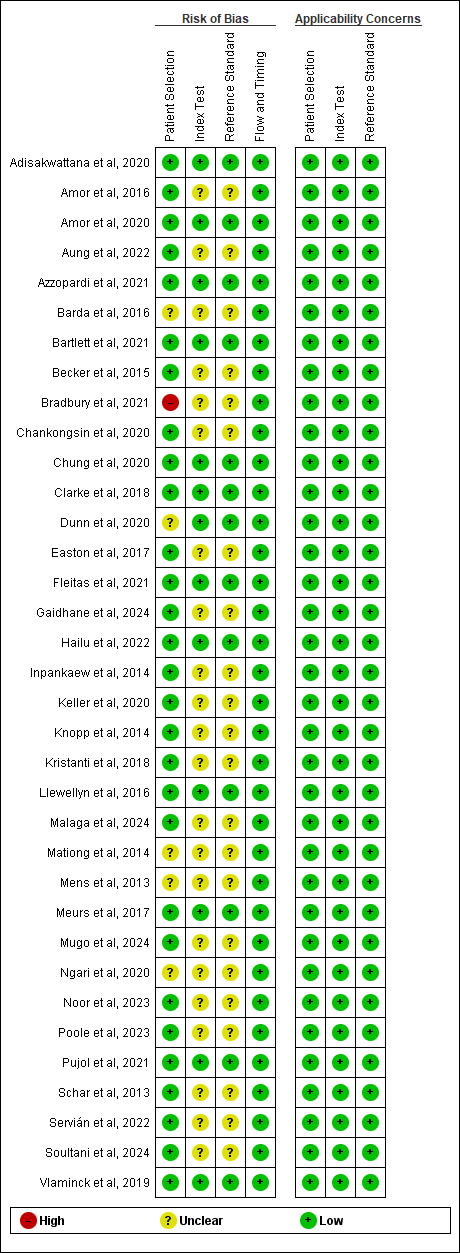


**Fig A. Risk of bias and applicability concerns for included studies for each separate domain based on Quality Assessment of Diagnostic Accuracy Studies 2 (QUADAS 2) tool.**


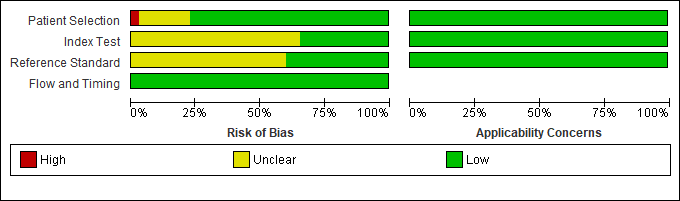


**Fig B. Summary of risk of bias and applicability concerns across the included studies based on Quality Assessment of Diagnostic Accuracy Studies 2 (QUADAS 2) tool.**


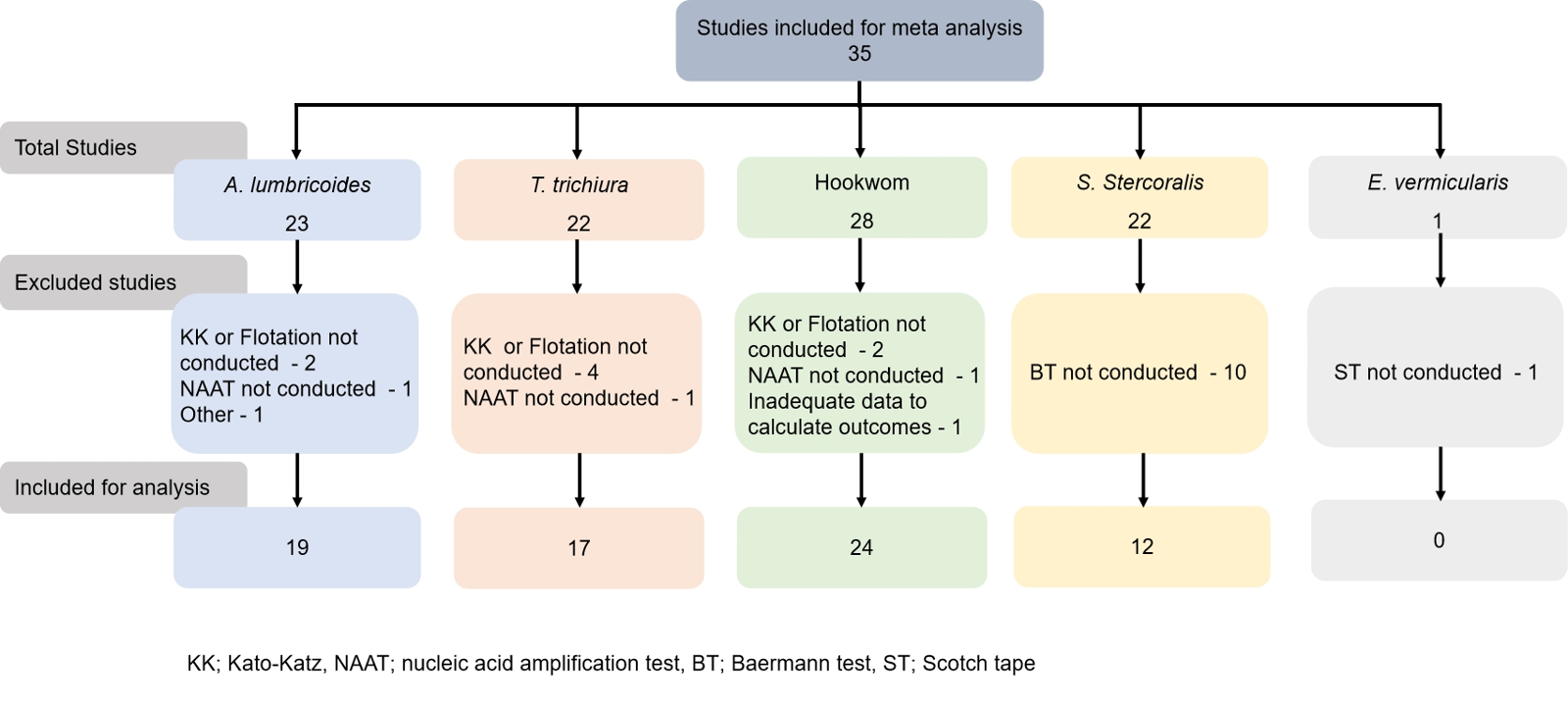


**Fig C. Details of the included studies**

KK; Kato-Katz, NAATs; nucleic acid amplification tests, BT; Baermann test, ST; Scotch tape


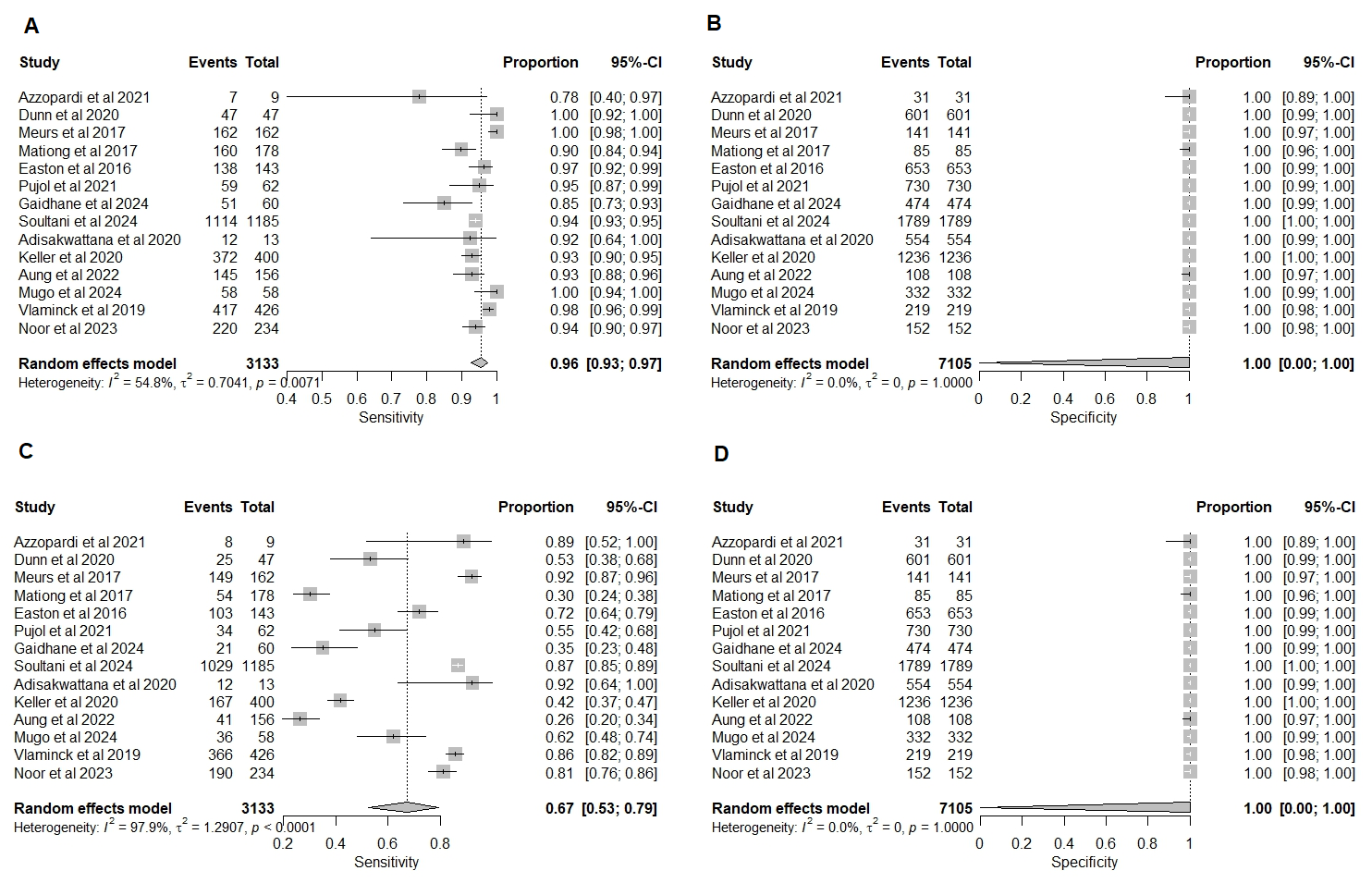


**Fig D. Forest plots for the diagnostic accuracy meta-analysis for *Ascaris lumbricoides*.** (A) Forest plot of sensitivity of nucleic acid amplification test (NAAT) compared to composite reference standard (CRS). (B) Forest plot of specificity of NAAT compared to CRS. (C) Forest plot of sensitivity of Kato-Katz (KK) compared to CRS. (D) Forest plot of specificity of KK compared to CRS.

CRS comprises the combination of NAATs and KK

The plots are generated using the Reitsma bivariate model in the mada package in R. Each study’s point estimate and 95% confidence interval are represented by squares and horizontal lines, respectively.


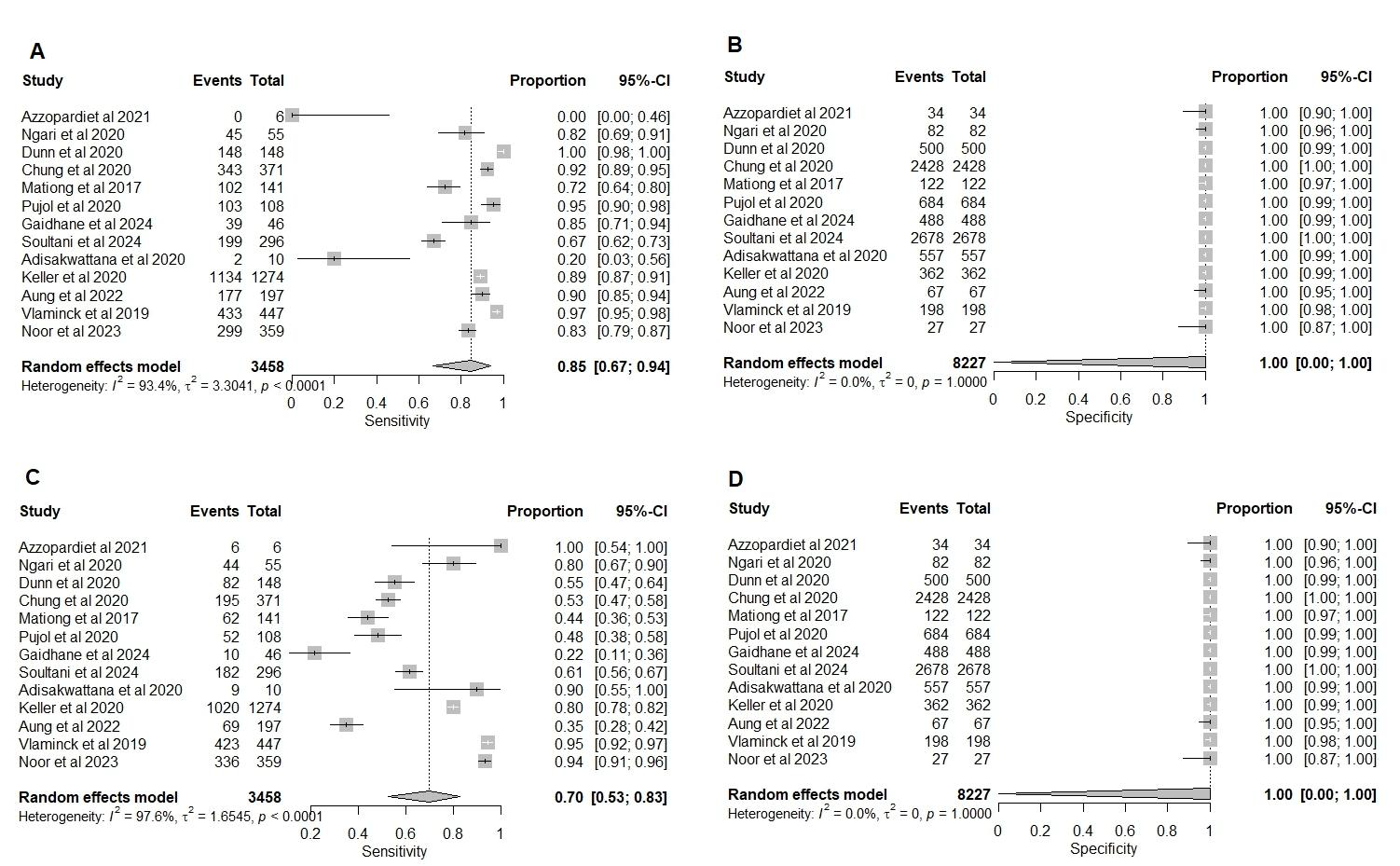


**Fig E. Forest plots for the diagnostic accuracy meta-analysis for *Trichuris trichiura*. (**A) Forest plot of sensitivity of nucleic acid amplification tests (NAATs) compared to composite reference standard (CRS). (B) Forest plot of specificity of NAATs compared to CRS. (C) Forest plot of sensitivity of Kato-Katz (KK) compared to CRS. (D) Forest plot of specificity of KK compared to CRS.

CRS comprises the combination of NAATs and KK.

The plots are generated using the Reitsma bivariate model in the mada package in R. Each study’s point estimate and 95% confidence interval are represented by squares and horizontal lines, respectively.


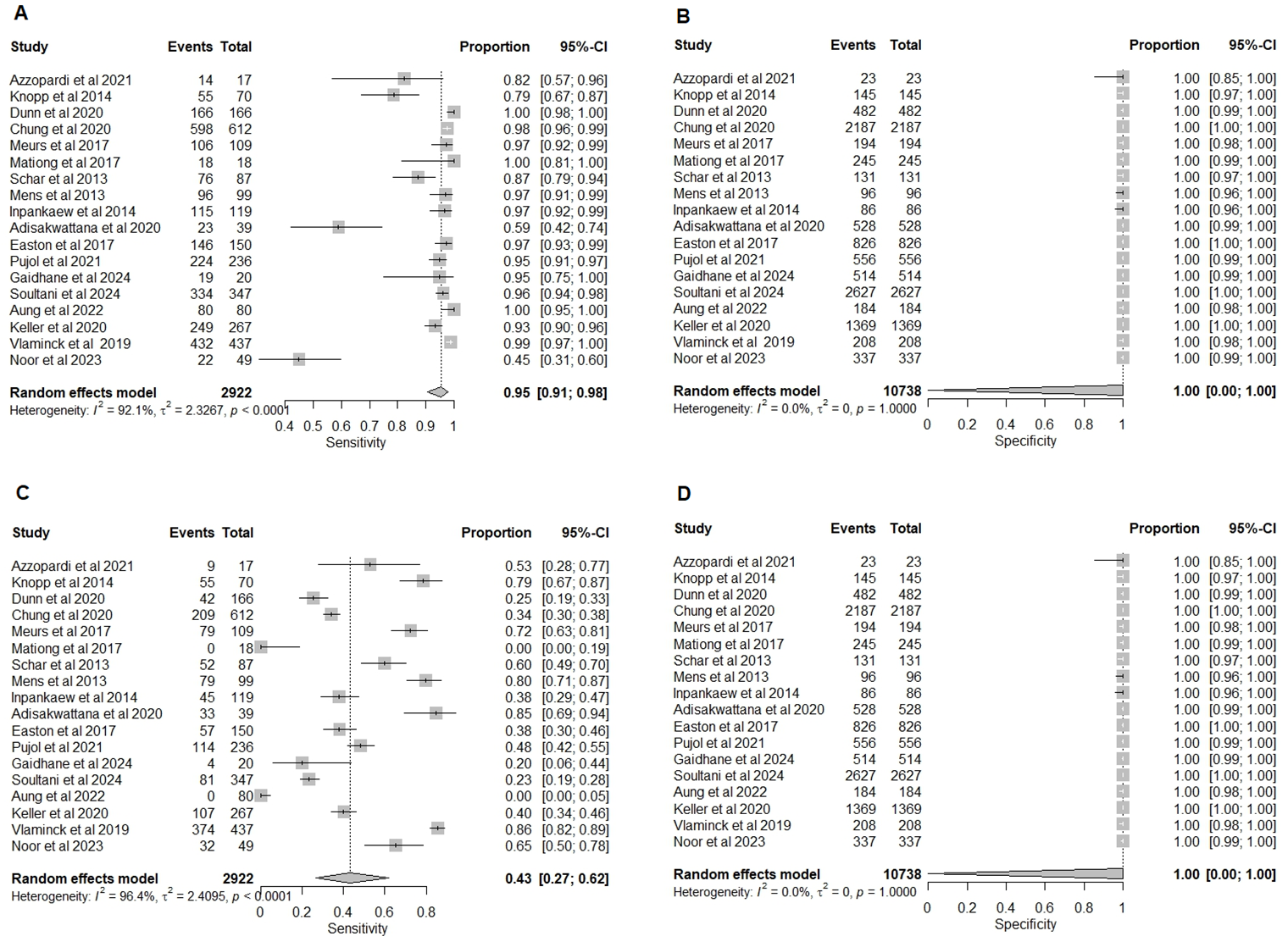


**Fig F. Forest plots for the diagnostic accuracy meta-analysis for hookworms. (**A) Forest plot of sensitivity of nucleic acid amplification tests (NAATs) compared to composite reference standard (CRS) (B) Forest plot of specificity of NAATs compared to CRS (C) Forest plot of sensitivity of Kato-Katz (KK) compared to CRS (D) Forest plot of specificity of KK compared to CRS.

CRS comprises the combination of NAATs and KK.

The plots are generated using the Reitsma bivariate model in the mada package in R. Each study’s point estimate and 95% confidence interval are represented by squares and horizontal lines, respectively.


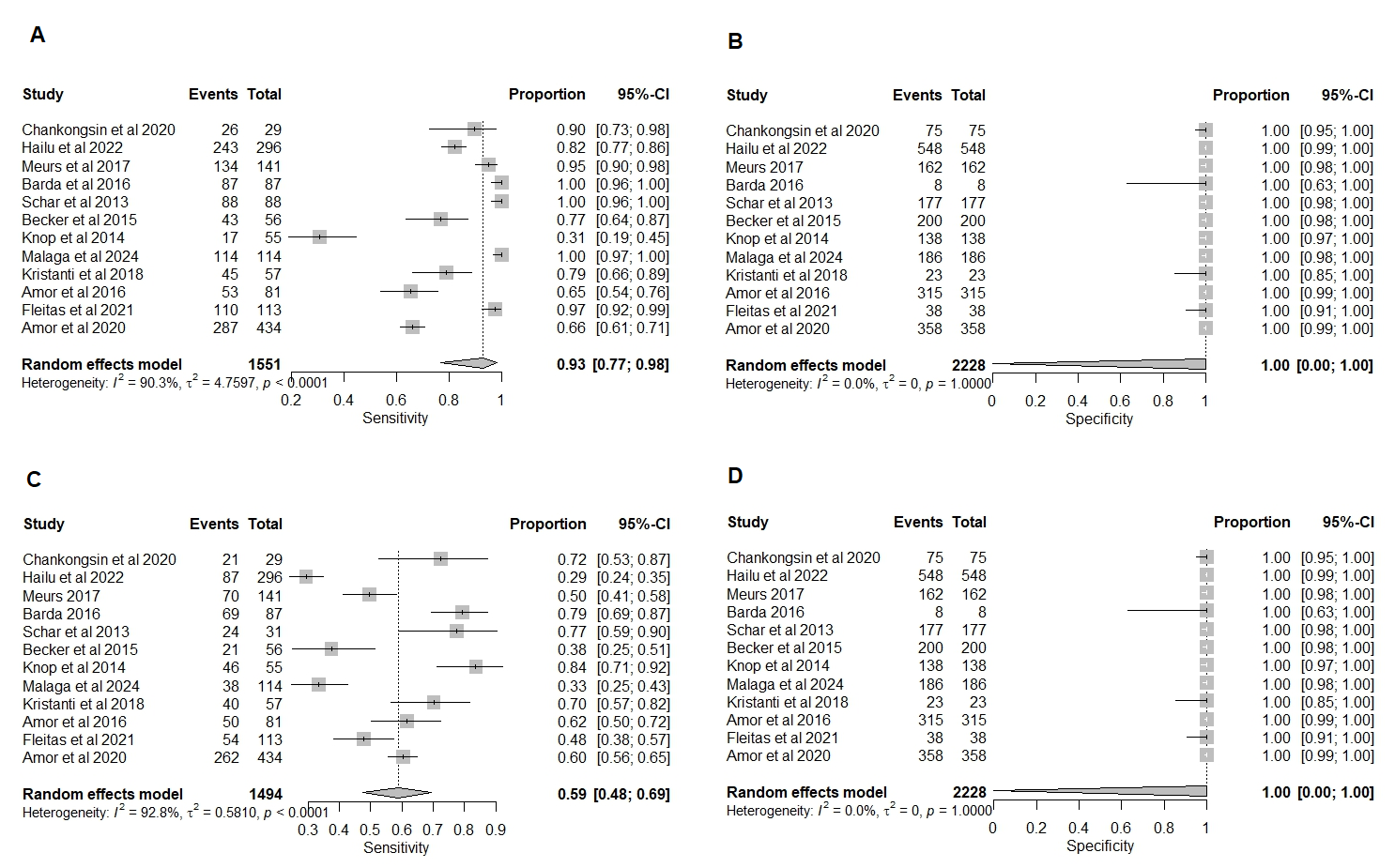


**Fig G. Forest plots for the diagnostic accuracy meta-analysis for *Strongyloides stercoralis*. (**A) Forest plot of sensitivity of nucleic acid amplification tests (NAATs) compared to composite reference standard (CRS). (B) Forest plot of specificity of NAATs compared to CRS. (C) Forest plot of sensitivity of Baermann test (BT) compared to CRS. (D) Forest plot of specificity of BT compared to CRS. CRS comprises the combination of NAATs and BT.

The plots are generated using the Reitsma bivariate model in the mada package in R. Each study’s point estimate and 95% confidence interval are represented by squares and horizontal lines, respectively.


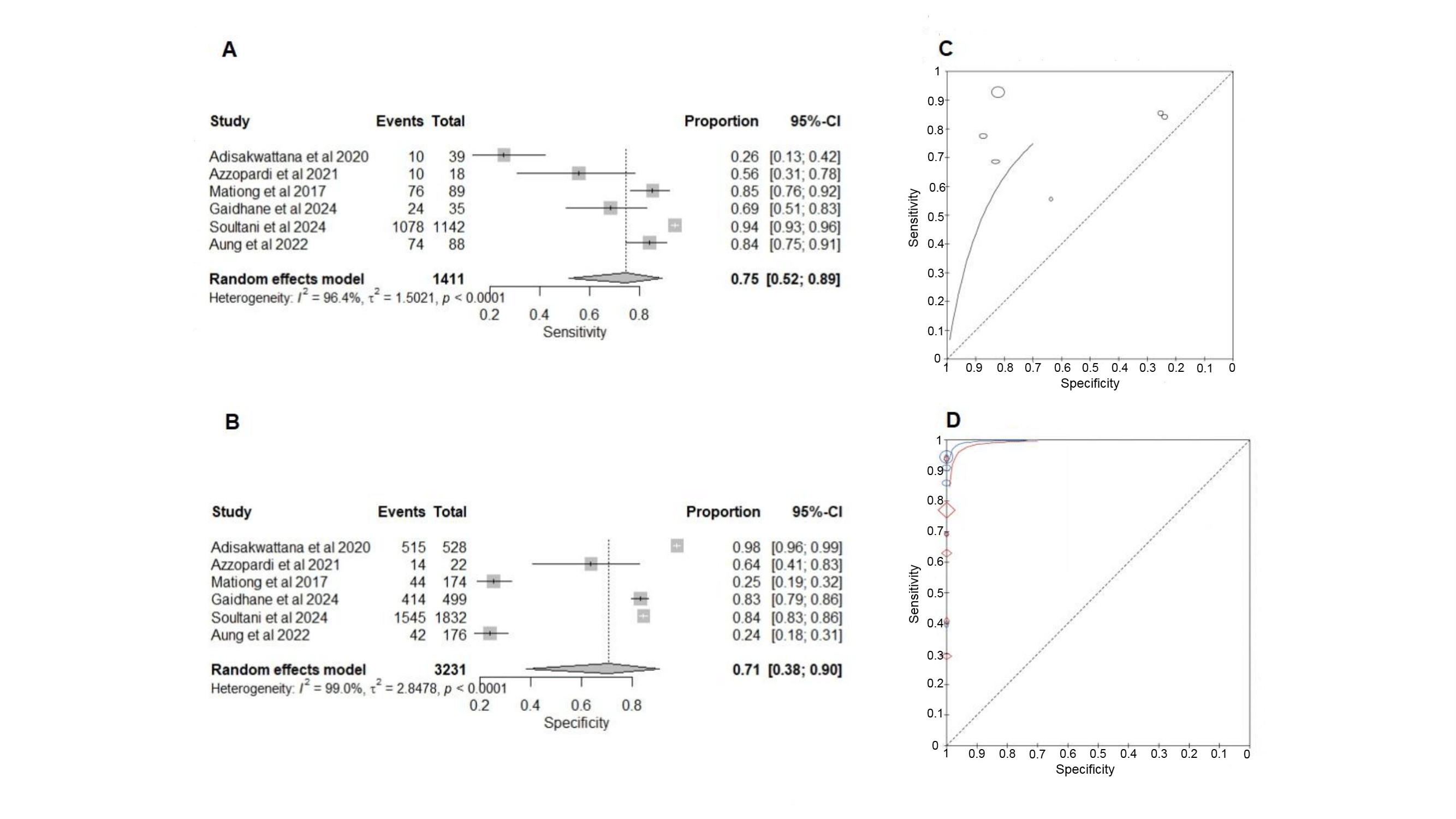


**Fig H. Forest plots and summary receiver operating curves for the diagnostic accuracy meta-analysis for soil-transmitted helminthes (*Ascaris*, *Trichuris* and hookworm). (**A) Forest plot of sensitivity of nucleic acid amplification tests (NAATs) compared to Kato-Katz (KK). (B) Forest plot of specificity of NAAT compared to KK. (C) sROC of NAATs and KK. (D) NAAT and Composite and KK and Composite. CRS comprises the combination of NAATs and KK.

The plots are generated using the Reitsma bivariate model in the mada package in R. Each study’s point estimate and 95% confidence interval are represented by squares and horizontal lines, respectively. The curves are generated using the Review Manager RevMan 5.4.1. The x-axis represents Specificity (False positive rate), and the y-axis represents Sensitivity (True positive rate).


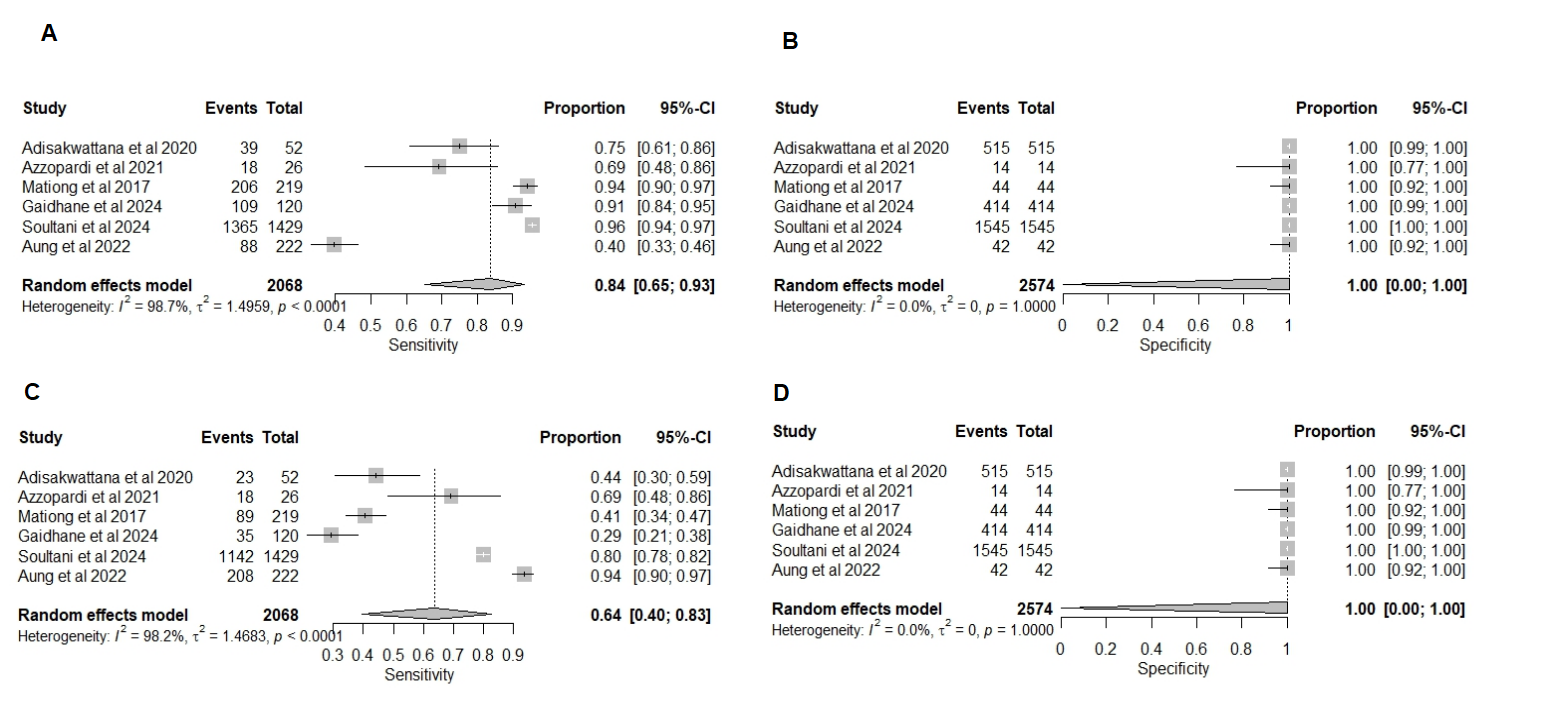


**Fig I. Forest plots for the diagnostic accuracy meta-analysis for soil-transmitted helminths. (**A) Forest plot of sensitivity of nucleic acid amplification tests (NAATs) compared to composite reference standard (CRS). (B) Forest plot of specificity of NAATs compared to CRS. (C) Forest plot of sensitivity of Kato-Katz (KK) compared to CRS. (D) Forest plot of specificity of KK compared to CRS.

CRS comprises the combination of NAATs and KK.

The plots are generated using the Reitsma bivariate model in the mada package in R. Each study’s point estimate and 95% confidence interval are represented by squares and horizontal lines, respectively.


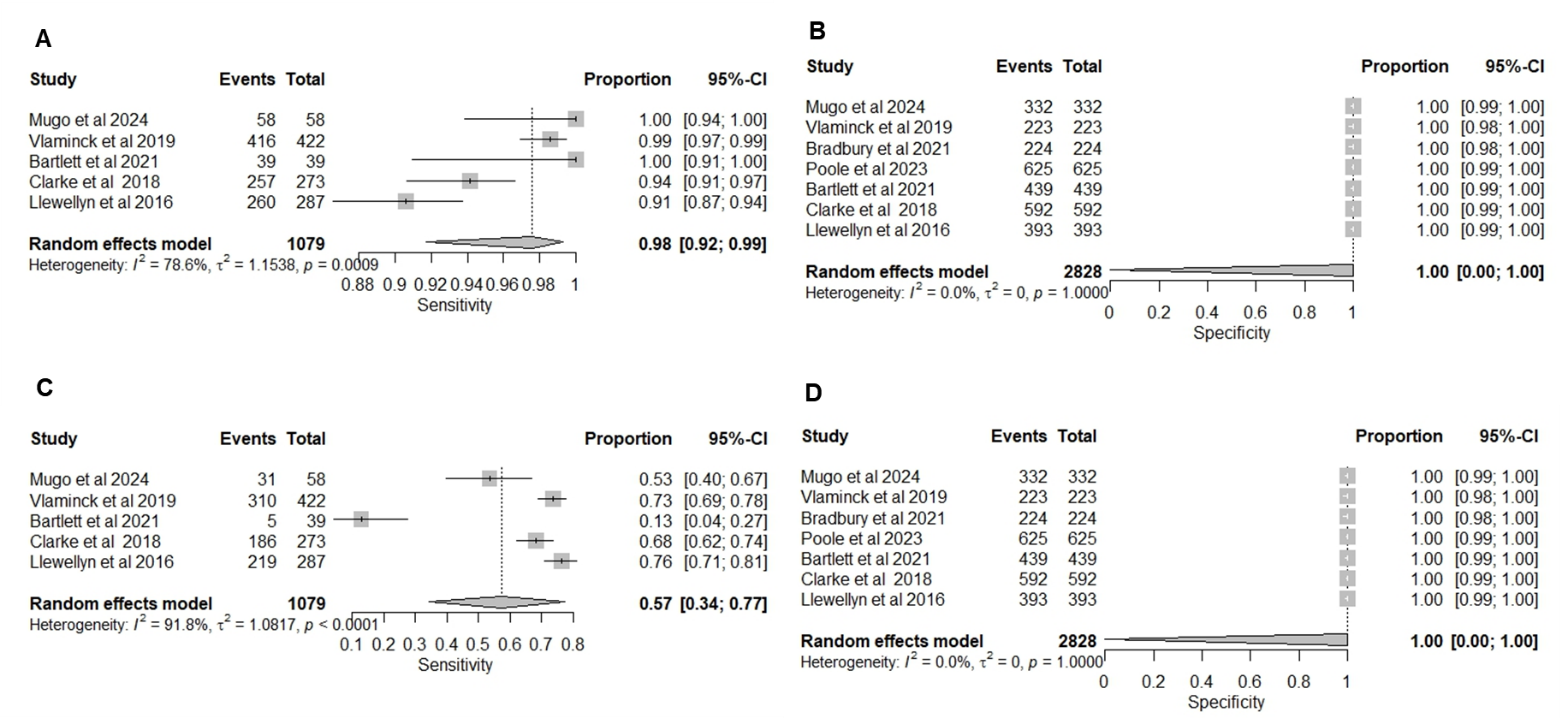


**Fig J. Forest plots for the diagnostic accuracy meta-analysis for *Ascaris lumbricoides*. (**A) Forest plot of sensitivity of nucleic acid amplification tests (NAATs) compared to composite reference standard (CRS). (B) Forest plot of specificity of NAATs compared to CRS. C: Forest plot of sensitivity of flotation methods compared to CRS, B: Forest plot of specificity of flotation methods compared to CRS. CRS comprises the combination of NAATs and flotation methods.

The plots are generated using the Reitsma bivariate model in the mada package in R. Each study’s point estimate and 95% confidence interval are represented by squares and horizontal lines, respectively.


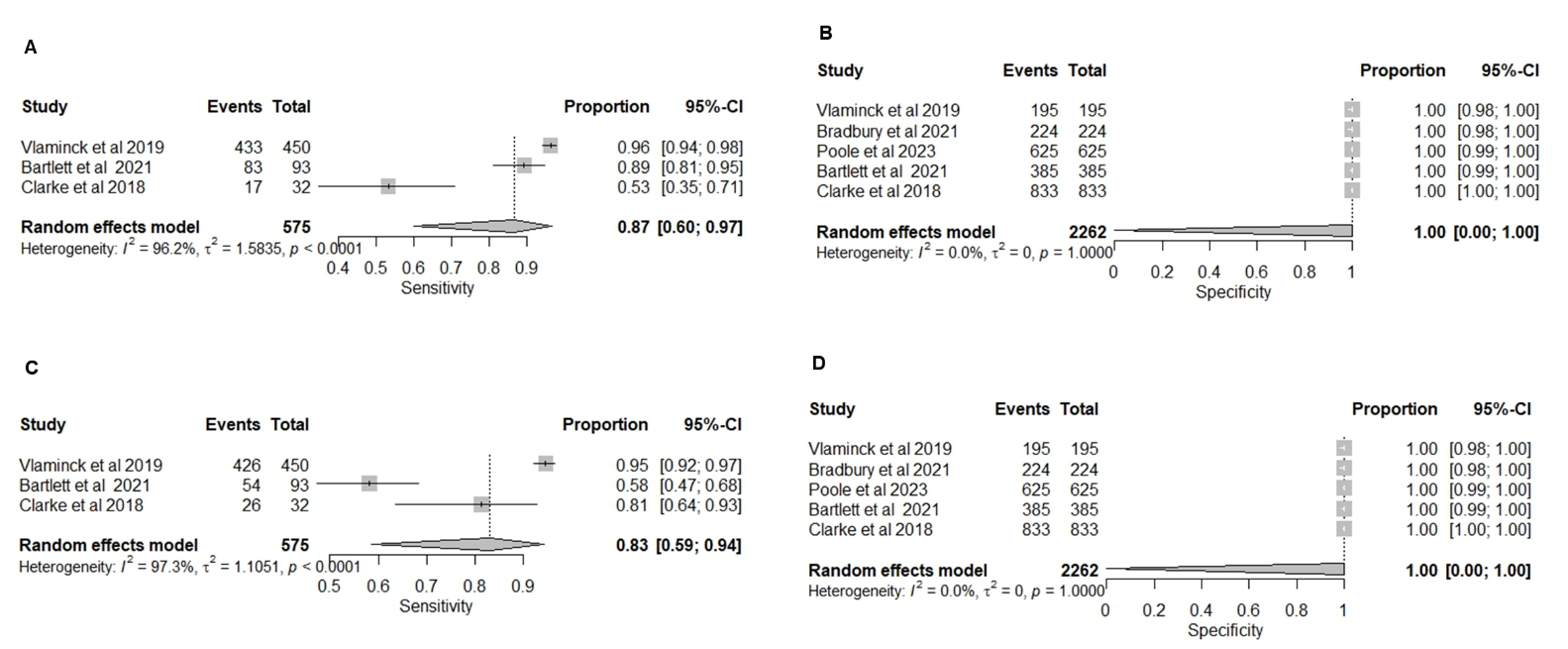


**Fig K. Forest plots for the diagnostic accuracy meta-analysis for *Trichuris trichiura*. (**A) Forest plot of sensitivity of nucleic acid amplification tests (NAATs) compared to composite reference standard (CRS). (B) Forest plot of specificity of NAATs compared to CRS. (C) Forest plot of sensitivity of flotation methods compared to CRS (D) Forest plot of specificity of flotation methods compared to CRS. CRS comprises the combination of NAATs and flotation methods.

The plots are generated using the Reitsma bivariate model in the mada package in R. Each study’s point estimate and 95% confidence interval are represented by squares and horizontal lines, respectively.

**
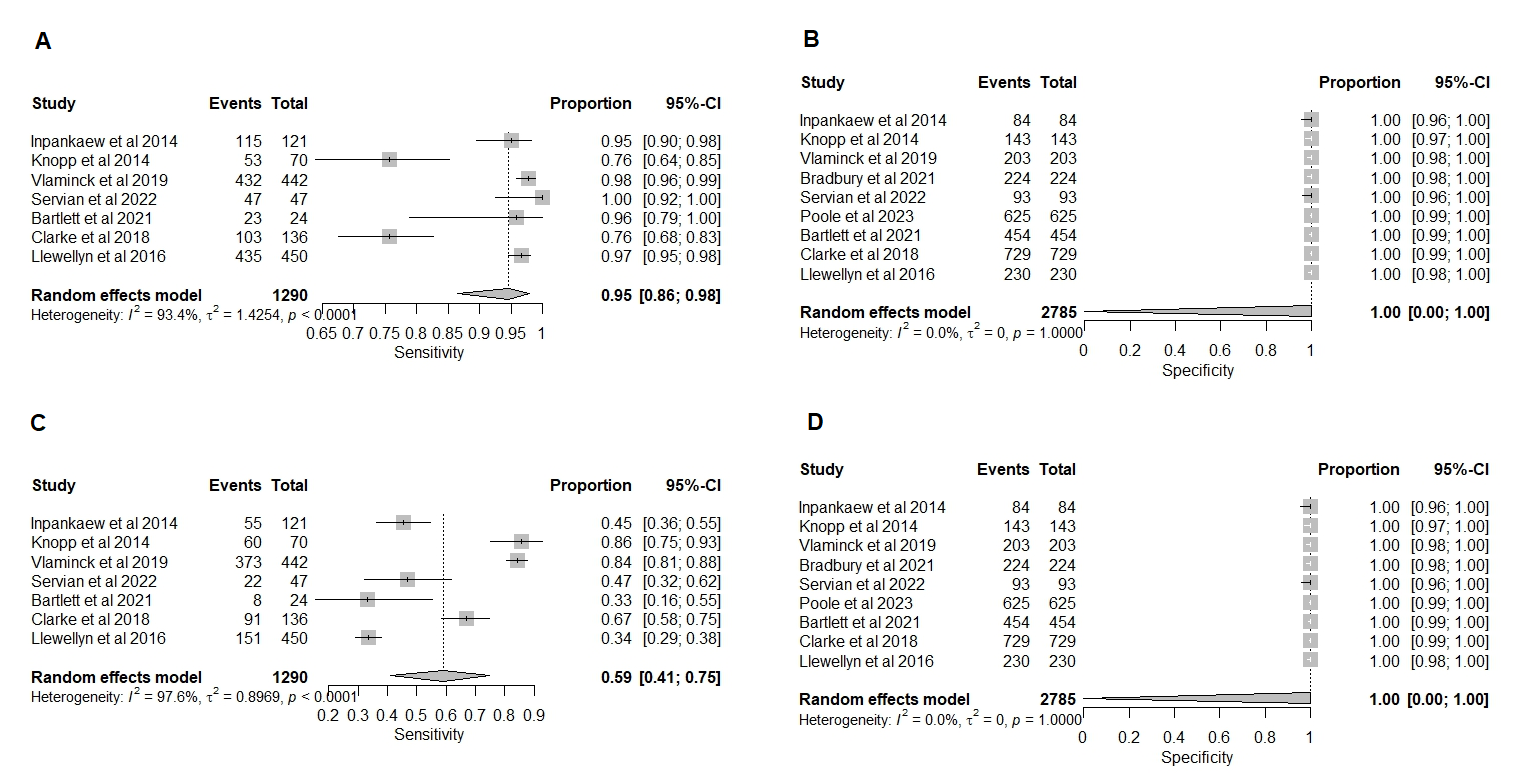
**

**Fig L. Forest plots for the diagnostic accuracy meta-analysis for hookworms. (**A) Forest plot of sensitivity of nucleic acid amplification tests (NAATs) compared to composite reference standard (CRS). (B) Forest plot of specificity of NAATs compared to CRS. (C) Forest plot of sensitivity of flotation methods compared to CRS. (D) Forest plot of specificity of flotation methods compared to CRS. CRS comprises the combination of NAATs and flotation methods.

The plots are generated using the Reitsma bivariate model in the mada package in R. Each study’s point estimate and 95% confidence interval are represented by squares and horizontal lines, respectively.


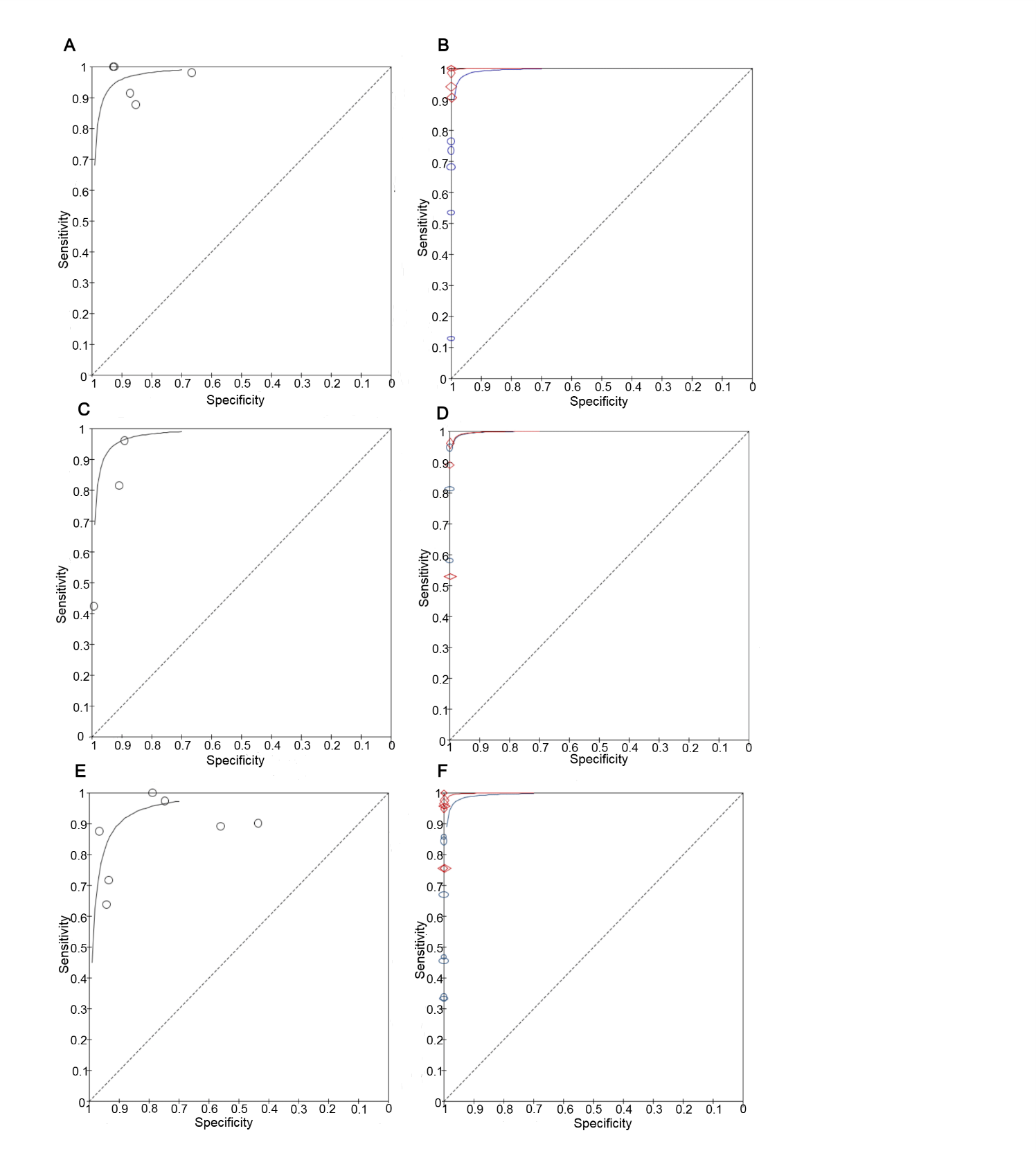


**Fig M. Summary receiver operating curves for the diagnostic accuracy meta-analysis for soil-transmitted helminthes (*Ascaris*, *Trichuris* and hookworm).** A: nucleic acid amplification tests (NAATs) compared to flotation methods for *Ascaris lumbricoides*, B: NAATs compared to CRS (blue) and flotation methods compared to CRS (red) for *Ascaris lumbricoides*, C: NAATs compared to flotation methods for *Trichuris trichiura*, D: NAATs compared to CRS (blue) and flotation methods and CRS (red) *Trichuris trichiura*. E: NAATs compared to flotation methods for hookworms, D: NAATs compared to CRS (blue) and flotation methods and CRS (red) for hookworms. CRS comprises the combination of NAATs and KK.

The curves are generated using the Review Manager RevMan 5.4.1. The x-axis represents Specificity (False positive rate), and the y-axis represents Sensitivity (True positive rate).

**References**

Studies included in the forest plots and QUADAS 2 assessments are cited using author and year labels, as generated by the analysis software, with full details provided below as numbered references starting from reference 1.

1. Adisakwattana P, Yoonuan T, Phuphisut O, Poodeepiyasawat A, Homsuwan N, Gordon CA, et al. Clinical helminthiases in Thailand border regions show elevated prevalence levels using qPCR diagnostics combined with traditional microscopic methods. Parasit Vectors. 2020 Aug 12;13(1):416. doi: 10.1186/s13071-020-04290-0.
2. Amor A, Rodriguez E, Saugar JM, Arroyo A, López-Quintana B, Abera B, et al. High prevalence of *Strongyloides stercoralis* in school-aged children in a rural highland of north-western Ethiopia: the role of intensive diagnostic work-up. Parasit Vectors. 2016 Dec 1;9(1):617. doi: 10.1186/s13071-016-1912-8.
3. Amor A, Anegagrie M, Zewdie D, Dacal E, Saugar JM, Herrador Z, et al. Epidemiology of intestinal helminthiases in a rural community of Ethiopia: Is it time to expand control programs to include *Strongyloides stercoralis* and the entire community? PLoS Negl Trop Dis. 2020 Jun 4;14(6):e0008315. doi: 10.1371/journal.pntd.0008315.
4. Aung E, Han KT, Gordon CA, Hlaing NN, Aye MM, Htun MW, et al. High prevalence of soil-transmitted helminth infections in Myanmar schoolchildren. Infect Dis Poverty. 2022 Mar 10;11(1):28. doi: 10.1186/s40249-022-00952-6.
5. Azzopardi KI, Hardy M, Baker C, Bonnici R, Llewellyn S, McCarthy JS, Traub RJ, Steer AC. Detection of six soil-transmitted helminths in human stool by qPCR- a systematic workflow. PLoS One. 2021 Sep 30;16(9):e0258039. doi: 10.1371/journal.pone.0258039.
6. Barda B, Wampfler R, Sayasone S, Phongluxa K, Xayavong S, Keoduangsy K, Schindler C, Keiser J. Evaluation of Two DNA Extraction Methods for Detection of *Strongyloides stercoralis* Infection. J Clin Microbiol. 2016 Mar 26;56(4):e01941-17. doi: 10.1128/JCM.01941-17.
7. Bartlett AW, Traub R, Amaral S, Hii SF, Clarke NE, Matthews A, et al. Comparison between Quantitative Polymerase Chain Reaction and Sodium Nitrate Flotation Microscopy in Diagnosing Soil-Transmitted Helminth Infections. Am J Trop Med Hyg . 2021;105(5):1210–3. doi: 10.4269/ajtmh.21-0227
8. Becker SL, Piraisoody N, Kramme S, Marti H, Silué KD, Panning M, et al. Real-time PCR for detection of *Strongyloides stercoralis* in human stool samples from Côte d’Ivoire: Diagnostic accuracy, inter-laboratory comparison and patterns of hookworm co-infection. Acta Trop. 2015;150:210–7. doi: 10.1016/j.actatropica.2015.07.019
9. Bradbury RS, Lane M, Arguello I, Handali S, Cooley G, Pilotte N, et al. Parasitic Disease Surveillance, Mississippi, USA. Emerg Infect Dis. 2021;27(8):2201–4. doi: 10.3201/eid2708.204318
10. Chankongsin S, Wampfler R, Ruf M-T, Odermatt P, Marti H, Nickel B, et al. *Strongyloides stercoralis* prevalence and diagnostics in Vientiane, Lao People’s Democratic Republic. Infect Dis Poverty. 2020;9(1):133. doi: 10.1186/s40249-020-00750-y
11. Benjamin-Chung J, Pilotte N, Ercumen A, Grant JR, Maasch JRMA, Gonzalez AM, et al. Comparison of multi-parallel qPCR and double-slide Kato-Katz for detection of soil-transmitted helminth infection among children in rural Bangladesh. PLoS Negl Trop Dis . 2020;14(4):e0008087. doi: 10.1371/journal.pntd.0008087
12. Clarke NE, Llewellyn S, Traub RJ, McCarthy J, Richardson A, Nery S V et al. Quantitative Polymerase Chain Reaction for diagnosis of soil-transmitted helminth infections: A comparison with a flotation-based technique and an investigation of variability in DNA detection. Am J Trop Med Hyg. 2018 ;99(4):1033–40. doi: 10.4269/ajtmh.18-0356
13. Dunn JC, Papaiakovou M, Han KT, Chooneea D, Bettis AA, Wyine NY, et al. The increased sensitivity of qPCR in comparison to Kato-Katz is required for the accurate assessment of the prevalence of soil-transmitted helminth infection in settings that have received multiple rounds of mass drug administration. Parasit Vectors. 2020;13(1):324. doi: 10.1186/s13071-020-04197-w
14. Easton A V., Oliveira RG, O’Connell EM, Kepha S, Mwandawiro CS, Njenga SM, et al. Multi-parallel qPCR provides increased sensitivity and diagnostic breadth for gastrointestinal parasites of humans: Field-based inferences on the impact of mass deworming. Parasites and Vectors. 2016 27;9(1):38. doi: 10.1186/s13071-016-1314-y
15. Fleitas PE, Vargas PA, Caro N, Almazan MC, Echazú A, Juárez M, et al. Scope and limitations of a multiplex conventional PCR for the diagnosis of *S. stercoralis* and hookworms. Brazilian J Infect Dis . 2021;25(6):101649. doi: 10.1016/j.bjid.2021.101649
16. Gaidhane S, Gaidhane A, Khatib MN, Telrandhe S, Patil M, Saxena D, et al. Estimation of the parasitic burden of soil-transmitted helminths among pregnant women in the Maharashtra State of India Using qPCR: A Community-Based Study. Indian J Community Med . 2024;49(1) doi: 10.4103/ijcm.ijcm_249_23
17. Hailu T, Amor A, Nibret E, Munshea A, Anegagrie M, Flores-Chavez MD, et al. Evaluation of five diagnostic methods for *Strongyloides stercoralis* infection in Amhara National Regional State, northwest Ethiopia. BMC Infect Dis . 2022;22(1):297. doi:10.1186/s12879-022-07299-1
18. Inpankaew T, Schär F, Khieu V, Muth S, Dalsgaard A, Marti H, et al. Simple fecal flotation is a superior alternative to guadruple kato katz smear examination for the detection of hookworm eggs in human stool. PLoS Negl Trop Dis . 2014;8(12):e3313. doi: 10.1371/journal.pntd.0003313
19. Keller L, Patel C, Welsche S, Schindler T, Hürlimann E, Keiser J et al. Performance of the Kato-Katz method and real-time polymerase chain reaction for the diagnosis of soil-transmitted helminthiasis in the framework of a randomised controlled trial: treatment efficacy and day-to-day variation. Parasit Vectors. 2020;13(1):517. doi: 10.1186/s13071-020-04401-x
20. Knopp S, Salim N, Schindler T, Voules DAK, Rothen J, Lweno O, et al. Diagnostic accuracy of Kato-Katz, FLOTAC, Baermann, and PCR methods for the detection of light-intensity hookworm and *Strongyloides stercoralis* infections in Tanzania. 2014;90(3):535–45. doi: 10.4269/ajtmh.13-0268
21. Kristanti H, Meyanti F, Wijayanti MA, Mahendradhata Y, Polman K, Chappuis F, et al. Diagnostic comparison of Baermann funnel, Koga agar plate culture and polymerase chain reaction for detection of human *Strongyloides stercoralis* infection in Maluku, Indonesia. Parasitol Res. 2018 ;117(10):3229–35. doi: 10.1007/s00436-018-6021-5
22. Llewellyn S, Inpankaew T, Nery SV, Gray DJ, Verweij JJ, Clements ACA, et al. Application of a multiplex quantitative PCR to assess prevalence and intensity of intestinal parasite infections in a controlled clinical trial. PLoS Negl Trop Dis . 2016 Jan ;10(1):e0004380. doi: 10.1371/journal.pntd.0004380
23. Malaga JL, Fernandez-Baca M V, Castellanos-Gonzalez A, Tanabe MB, Tift C, Morales ML, et al. The recombinase polymerase amplification test for *Strongyloides stercoralis i*s more sensitive than microscopy and real-time pcr in high-risk communities of Cusco, Peru. 2024;13(10):869. doi: 10.3390/pathogens13100869
24. Mationg MLS, Gordon CA, Tallo VL, Olveda RM, Alday PP, Reñosa MDC, et al. Status of soil-transmitted helminth infections in schoolchildren in Laguna Province, the Philippines: Determined by parasitological and molecular diagnostic techniques. PLoS Negl Trop Dis . 2017 ;11(11):e0006022. doi: 10.1371/journal.pntd.0006022
25. van Mens SP, Aryeetey Y, Yazdanbakhsh M, van Lieshout L, Boakye D, Verweij JJ. Comparison of real-time PCR and Kato smear microscopy for the detection of hookworm infections in three consecutive faecal samples from schoolchildren in Ghana. Trans R Soc Trop Med Hyg. 2013;107(4):269–71. doi: 10.1093/trstmh/trs094
26. Meurs L, Polderman AM, Vinkeles Melchers NVS, Brienen EAT, Verweij JJ, Groosjohan B, et al. Diagnosing polyparasitism in a high-prevalence setting in Beira, Mozambique: detection of intestinal parasites in fecal samples by microscopy and real-time PCR. PLoS Negl Trop Dis . 2017;11(1):e0005310. doi.org/10.1371/journal.pntd.0005310
27. Mugo RM, Rausch S, Musimbi ZD, Strube C, Raulf M-K, Landt O, et al. Evaluation of copromicroscopy, multiplex-qPCR and antibody serology for monitoring of human ascariasis in endemic settings. PLoS Negl Trop Dis . 2024 ;18(6):e0012279. doi.org/10.1371/journal.pntd.0012279
28. Ngari MG, Mwangi IN, Njoroge MP, Kinyua J, Osuna FA, Kimeu BM, et al. Development and evaluation of a loop-mediated isothermal amplification (LAMP) diagnostic test for detection of whipworm, *Trichuris trichiura,* in faecal samples. J Helminthol . 2020;94:e142. doi: 10.1017/S0022149X2000022X
29. Noor Z, Hossain B, Khan SS, Kabir M, Bhuiyan ATMRH, Alam MS, et al. Prevalence of Soil-Transmitted Helminths at Baseline and after Albendazole Treatment in the school-age children of forcibly displaced Myanmar nationals in Bangladesh. Am J Trop Med Hyg. 2023;109(3):656–66. doi: 10.4269/ajtmh.23-0260
30. Poole C, Barker T, Bradbury R, Capone D, Chatham AH, Handali S, et al. Cross-sectional study of soil-transmitted helminthiases in black belt region of Alabama, USA. Emerg Infect Dis. 2023;29(12):2461–70. doi: 10.3201/eid2912.230751
31. Grau-Pujol B, Martí-Soler H, Escola V, Demontis M, Jamine JC, Gandasegui J, et al. Towards soil-transmitted helminths transmission interruption: The impact of diagnostic tools on infection prediction in a low intensity setting in Southern Mozambique. PLoS Negl Trop Dis . 2021;15(10):e0009803. doi: 10.1371/journal.pntd.0009803
32. Schär F, Odermatt P, Khieu V, Panning M, Duong S, Muth S, et al. Evaluation of real-time PCR for S*trongyloides stercoralis* and hookworm as diagnostic tool in asymptomatic schoolchildren in Cambodia. 2013 May;126(2):89–92. doi: 10.1016/j.actatropica.2012.12.012
33. Servián A, Repetto SA, Lorena Zonta M, Navone GT. Human hookworms from Argentina: Differential diagnosis of *Necator americanus* and *Ancylostoma duodenale* in endemic populations from Buenos Aires and Misiones. Rev Argent Microbiol. 2022;54(4):268–81. doi: 10.1016/j.ram.2022.05.005
34. Soultani M, Bartlett AW, Mendes EP, Hii SF, Traub R, Palmeirim MS, et al. Estimating Prevalence and infection intensity of soil-transmitted helminths using quantitative polymerase chain reaction and Kato-Katz in school-age children in Angola. Am J Trop Med Hyg. 2024;110(6):1145–51. doi: 10.4269/ajtmh.23-0821
35. Vlaminck J, Cools P, Albonico M, Ame S, Ayana M, Cringoli G, et al. Therapeutic efficacy of albendazole against soil-transmitted helminthiasis in children measured by five diagnostic methods. PLoS Negl Trop Dis . 2019 ;13(8):e0007471. doi: 10.1371/journal.pntd.0007471
